# Approximate search for known gene clusters in new genomes using PQ-trees

**DOI:** 10.1186/s13015-021-00190-9

**Published:** 2021-07-09

**Authors:** Galia R. Zimerman, Dina Svetlitsky, Meirav Zehavi, Michal Ziv-Ukelson

**Affiliations:** Department of Computer Science, Ben Gurion University of the Negev, Be’er Sheva, Israel

**Keywords:** PQ-tree, Gene cluster, Efflux pump

## Abstract

Gene clusters are groups of genes that are co-locally conserved across various genomes, not necessarily in the same order. Their discovery and analysis is valuable in tasks such as gene annotation and prediction of gene interactions, and in the study of genome organization and evolution. The discovery of conserved gene clusters in a given set of genomes is a well studied problem, but with the rapid sequencing of prokaryotic genomes a new problem is inspired. Namely, given an already known gene cluster that was discovered and studied in one genomic dataset, to identify all the instances of the gene cluster in a given new genomic sequence. Thus, we define a new problem in comparative genomics, denoted PQ-Tree Search that takes as input a PQ-tree *T* representing the known gene orders of a gene cluster of interest, a gene-to-gene substitution scoring function *h*, integer arguments $d_T$ and $d_S$, and a new sequence of genes *S*. The objective is to identify in *S* approximate new instances of the gene cluster; These instances could vary from the known gene orders by genome rearrangements that are constrained by *T*, by gene substitutions that are governed by *h*, and by gene deletions and insertions that are bounded from above by $d_T$ and $d_S$, respectively. We prove that PQ-Tree Search is NP-hard and propose a parameterized algorithm that solves the optimization variant of PQ-Tree Search in $O^*(2^{\gamma })$ time, where $\gamma$ is the maximum degree of a node in *T* and $O^*$ is used to hide factors polynomial in the input size. The algorithm is implemented as a search tool, denoted PQFinder, and applied to search for instances of chromosomal gene clusters in plasmids, within a dataset of 1,487 prokaryotic genomes. We report on 29 chromosomal gene clusters that are rearranged in plasmids, where the rearrangements are guided by the corresponding PQ-trees. One of these results, coding for a heavy metal efflux pump, is further analysed to exemplify how PQFinder can be harnessed to reveal interesting new structural variants of known gene clusters.

**Supplementary Information:**

The online version contains supplementary material available at 10.1186/s13015-021-00190-9.

## Introduction

Recent advances in pyrosequencing techniques, combined with global efforts to study infectious diseases, yield huge and rapidly-growing databases of microbial genomes [3, 4]. These big new data statistically empower genomic-context based approaches to functional analysis: the biological principle underlying such analysis is that groups of genes that are located close to each other across many genomes often code for proteins that interact with one another, suggesting a common functional association. Thus, if the functional association and annotation of the clustered genes is already known in one (or more) of the genomes, this information can be used to infer functional characterization of homologous genes that are clustered together in another genome.

Groups of genes that are co-locally conserved across many genomes are denoted *gene clusters*. The locations of the group of genes comprising a gene cluster in the distinct genomes are denoted *instances*. Gene clusters in prokaryotic genomes often correspond to (one or several) operons; those are neighbouring genes that constitute a single unit of transcription and translation. However, the order of the genes in the distinct instances of a gene cluster may not be the same.

The discovery (i.e. data-mining) of conserved gene clusters in a given set of genomes is a well studied problem [5–7]. However, with the rapid sequencing of prokaryotic genomes a new problem is inspired. Namely, given an already known gene cluster that was discovered and studied in one genomic dataset, to identify all the instances of the gene cluster in a given new genomic sequence.

One exemplary application for this problem is the search for chromosomal gene clusters in plasmids. Plasmids are circular genetic elements that are harbored by prokaryotic cells where they replicate independently from the chromosome. They can be transferred horizontally and vertically, and are considered a major driving force in prokaryotic evolution, providing mutation supply and constructing new operons with novel functions [8], for example antibiotic resistance [9]. This motivates biologists to search for chromosomal gene clusters in plasmids, and to study structural variations between the instances of the found gene clusters across the two distinct replicons. However, in addition to the fact that plasmids evolve independently from chromosomes and in a more rapid pace [10], their sequencing, assembly and annotation involves a more noisy process [11].

To accommodate all this, the proposed search approach should be an approximate one, sensitive enough to tolerate some amount of genome rearrangements: transpositions and inversions, missing and intruding genes, and classification of genes with similar function to distinct orthology groups due to sequence divergence or convergent evolution. Yet, for the sake of specificity and search efficiency, we consider confining the allowed variations by two types of biological knowledge: (1) bounding the allowed rearrangement events considered by the search, based on some grammatical model trained specifically from the known gene orders of the gene cluster, and (2) governing the gene-to-gene substitutions considered by the search by combining sequence homology with functional-annotation based semantic similarity.

**Bounding the allowed rearrangement events.** The PQ-tree [12] is a combinatorial data structure classically used to represent gene clusters [13]. A PQ-tree of a gene cluster describes its hierarchical inner structure and the relations between instances of the cluster succinctly, aids in filtering meaningful from apparently meaningless clusters, and also gives a natural and meaningful way of visualizing complex clusters. A PQ-tree is a rooted tree with three types of nodes: *P-nodes*, *Q-nodes* and leaves. The children of a P-node can appear in any order, while the children of a Q-node must appear in either left-to-right or right-to-left order. (In the special case when a node has exactly two children, it does not matter whether it is labeled as a P-node or a Q-node.) Booth and Lueker [12], who introduced this data structure, were interested in representing a set of permutations over a set *U*, i.e. every member of *U* appears exactly once as a label of a leaf in the PQ-tree. We, on the other hand, allow each member of *U* to appear as a label of a leaf in the tree any non-negative number of times. Therefore, we will henceforth use the term *string* rather than *permutation* when describing the gene orders derived from a given PQ-tree.

**Fig. 1:**
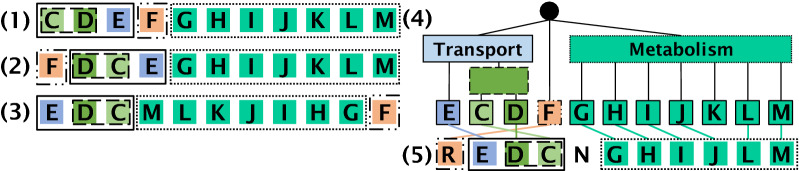
A gene cluster containing most of the genes of the *PhnCDEFGHIJKLMNOP* operon [14] and the corresponding PQ-tree. The *Phn* operon encodes proteins that utilize phosphonate as a nutritional source of phosphorus in prokaryotes. The genes *PhnCDE* encode a phosphonate transporter, the genes *PhnGHIJKLM* encode proteins responsible for the conversion of phosphonates to phosphate, and the gene *PhnF* encodes a regulator. (1)–(3). The three distinct gene orders found among 47 chromosomal instances of the *Phn* gene cluster. (4) A PQ-tree representing the *Phn* gene cluster, constructed from its three known gene orders shown in (1)–(3). (5) An example of a *Phn* gene cluster instance identified by the PQ-tree shown in (4), and the one-to-one mapping between the leaves of the PQ-tree and the genes comprising the instance (indicated by the colored lines). The instance genes are rearranged differently from the gene orders shown in (1)-(3) and yet can be derived from the PQ-tree. In this mapping, gene *F* is substituted by gene *R*, gene *N* is an intruding gene (i.e., deleted from the instance string), and gene *K* is a missing gene (i.e., deleted from the PQ-tree)

An example of a PQ-tree is given in Fig. 1. It represents a *Phn* gene cluster that encodes proteins that utilize phosphonate as a nutritional source of phosphorus in prokaryotes [14]. The biological assumptions underlying the representation of gene clusters as PQ-trees is that operons evolve via progressive merging of sub-operons, where the most basic units in this recursive operon assembly are colinearly conserved sub-operons [15]. In the case where an operon is assembled from sub-operons that are colinearly dependent, the conserved gene order could correspond, e.g., to the order in which the transcripts of these genes interact in the metabolic pathway in which they are functionally associated [16]. Thus, transposition events shuffling the order of the genes within this sub-operon could reduce its fitness. On the other hand, inversion events, in which the genes participating in this sub-operon remain colinearly ordered are accepted. This case is represented in the PQ-tree by a Q-node (marked with a rectangle). In the case where an operon is assembled from sub-operons that are not colinearly co-dependent, convergent evolution could yield various orders of the assembled components [15]. This case is represented in the PQ-tree by a P-node (marked with a circle). Learning the internal topology properties of a gene cluster from its corresponding gene orders and constructing a query PQ-tree accordingly, could empower the search to confine the allowed rearrangement operations so that colinear dependencies among genes and between sub-operons are preserved.

**Governing the gene-to-gene substitutions.** A prerequisite for gene cluster discovery is to determine how genes relate to each other across all the genomes in the dataset. In our experiment, genes are represented by their membership in Clusters of Orthologous Groups (COGs) [17], where the sequence similarity of two genes belonging to the same COG serves as a proxy for homology. Despite low sequence similarity, genes belonging to two different COGs could have a similar function, which would be reflected in the functional description of the respective COGs. Using methods from natural language processing [18], we compute for each pair of functional descriptions a score reflecting their semantic similarity. Combining sequence and functional similarity could increase the sensitivity of the search and promote the discovery of systems with related functions.

**Our contribution and roadmap.** We define two new problems in comparative genomics, denoted PQ-Tree Search and PQ-Tree Alignment (in "Preliminaries" section), where the second is a sub-problem of the first. Both problems take as input a PQ-tree *T* (the query) representing the known gene orders of a gene cluster of interest, a gene-to-gene substitution scoring function *h*, integer arguments $d_T$ and $d_S$, and a sequence of genes *S* (the target). The objective in PQ-Tree Search is to identify an approximate instance $S'$ of the gene cluster, such that $S'$ is a substring of *S*. The objective of PQ-Tree Alignment is to determine whether $S'$ is an approximate instance of the gene cluster; An approximate instance could vary from the known gene orders by genome rearrangements that are constrained by *T*, by gene substitutions that are governed by *h*, and by gene deletions and insertions that are bounded from above by $d_T$ and $d_S$, respectively. We prove that both PQ-Tree Search and PQ-Tree Alignment are NP-hard (Theorems 2, 3 in "PQ-tree search is NP-hard" section).

We define optimization variants of PQ-Tree Search and PQ-Tree Alignment (in " Preliminaries" section) and propose an algorithm (in "A parameterized algorithm" section) that solves PQ-Tree Search in $O(n \gamma {d_T}^2 {d_S}^2 (m_p \cdot 2^{\gamma } + m_q))$ time, where *n* is the length of *S*, $m_p$ and $m_q$ denote the number of P-nodes and Q-nodes in *T*, respectively, and $\gamma$ denotes the maximum degree of a node in *T*. The proposed algorithm for PQ-Tree Search solves PQ-Tree Alignment for every substring of *S*. Thus, in the same time and space complexities, we can also report all approximate instances of *T* in *S* and not only the optimal one.

The algorithm is implemented as a search tool, denoted PQFinder. The code for the tool as well as all the data needed to reconstruct the results are publicly available on GitHub [2]. The tool is applied to search for instances of chromosomal gene clusters in plasmids, within a dataset of 1,487 prokaryotic genomes; methods are given in "Methods and datasets" section. In our preliminary results (in "Results" section), we report on 29 chromosomal gene clusters that are rearranged in plasmids, where the rearrangements are guided by the corresponding PQ-tree. One of these results, coding for a heavy metal efflux pump, is further analysed to exemplify how PQFinder can be harnessed to reveal interesting new structural variants of known gene clusters.

**Previous related works.** Permutations on strings representing gene clusters have been studied earlier by [19–23]. PQ-trees were previously applied in physical mapping [24, 25], as well as to other comparative genomics problems [26–28].

In Landau et al. [28] an algorithm was proposed for representation and detection of gene clusters in multiple genomes, using PQ-trees: the proposed algorithm computes a PQ-tree of *k* permutations of length *n* in *O*(*kn*) time, and it is proven that the computed PQ-tree is the one with a minimum number of possible rearrangements of its nodes while still representing all *k* permutations. In the same paper, the authors also present a general scheme to handle gene multiplicity and missing genes in permutations. For every character that appears *a* times in each of the *k* strings, the time complexity for the construction of the PQ-tree, according to the scheme in that paper, is multiplied by an $O((a!)^k)$ factor.

Additional applications of PQ-trees to genomics were studied in [29–31], where PQ-trees were considered to represent and reconstruct ancestral genomes.

However, as far as we know, searching for approximate instances of a gene cluster that is represented as a PQ-tree, in a given new string, is a new computational problem.

Semantic similarity measures between Gene Ontology (GO) terms [32] have been previously used in tasks such as protein function prediction [33, 34], functional enrichment analysis of gene expression datasets [35, 36], and protein-protein interaction inference [37, 38]. In the context of gene cluster analysis, a recent study mined gene clusters that have common functional associations among seven amniote genomes, by measuring the GO term similarity of the respective genes [39]. However, the Gene Ontology Consortium provides annotations for only 41 prokaryotic genomes, while the dataset used in this study consists of 1487 prokaryotic genomes. Transferring GO annotations from the annotated genomes to the other genomes in our dataset using gene sequence similarity would lead to a limited gene coverage. Therefore, in this study we use COG functional descriptions to measure semantic similarity between genes.

## Preliminaries

Let $\Pi$ be an NP-hard problem. In the framework of Parameterized Complexity, each instance of $\Pi$ is associated with a *parameter* *k*, and the goal is to confine the combinatorial explosion in the running time of an algorithm for $\Pi$ to depend only on *k*. Formally, $\Pi$ is *fixed-parameter tractable (*FPT*)* if any instance (*I*, *k*) of $\Pi$ is solvable in time $f(k)\cdot |I|^{{\mathcal {O}}(1)}$, where *f* is an arbitrary computable function of *k*. Nowadays, Parameterized Complexity supplies a rich toolkit to design or refute the existence of FPT algorithms [40–42].

### PQ-tree: representing the pattern.

The possible reordering of nodes in a PQ-tree may create many equivalent PQ-trees. In [12] two PQ-trees *T* and $T'$ are defined as *equivalent* (denoted $T \equiv T'$) if one tree can be obtained by legally reordering the nodes of the other; namely, randomly permuting the children of a P-node, and reversing the children of a Q-node. To allow for deletions in the PQ-trees, a generalization of that definition is given in Definition 1 below. Here, *smoothing* is a recursive process in which if by deleting leaves from a tree *T*, some internal node *x* of *T* is left without children, then *x* is also deleted, but its deletion is not counted (i.e. only leaf deletions are counted).

#### Definition 1

(Quasi-Equivalence Between PQ-Trees) For any two PQ-trees, *T* and $T'$, the PQ-tree *T* is *quasi-equivalent to* $T'$ *with a bound* *d*, denoted $T\succeq _d T'$, if $T'$ can be obtained from *T* by (a) randomly permuting the children of some of the P-nodes of *T*, (b) reversing the children of some of the Q-nodes of *T*, and (c) deleting up to *d* leaves from *T* and applying the corresponding smoothing. (The order of the operations does not matter.)

**Fig. 2:**
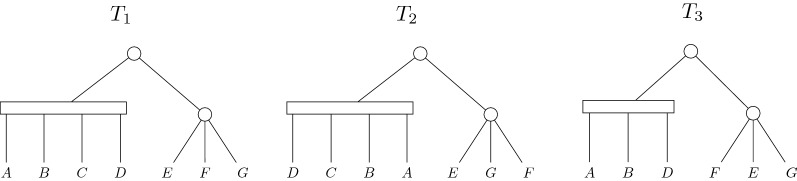
Exemplification of three different PQ-trees. $T_2$ can be obtained from $T_1$ by reversing the children of a Q-node (the left child of the root) and by reordering the children of a P-node (the right child of the root), so $T_2 \equiv T_1$. The PQ-tree $T_3$ can be obtained from $T_1$ by deleting one leaf and permuting the children of the right child of the root, so $T_1 \succeq _1 T_3$. Now, $T_2 \succeq _1 T_3$ can be inferred because the $\equiv$ is an equivalence relation. By the definition of frontier, $F(T_1)=ABCDEFG$; $F(T_2)=DCBAEGF$; $F(T_3)=ABDFEG$

Figure 2 shows two equivalent PQ-trees ($T_1$ and $T_2$) that are each quasi-equivalent with $d=1$ to the third PQ-tree ($T_3$). The *frontier* of a PQ-tree *T*, denoted *F*(*T*), is the sequence of labels on the leaves of *T* read from left to right. For example, the frontier of the PQ-tree $T_1$ in Fig. 2 is *ABCDEFG*. It is interesting to consider the set of frontiers of all the equivalent PQ-trees, defined in [12] as a *consistent set* and denoted by $C(T)=\{F(T'):T\equiv T'\}$. Intuitively, *C*(*T*) is the set of all leaf label sequences defined by the PQ-tree structure and obtained by legally reordering its nodes. Here, we generalize the consistent set definition to allow a bounded number of deletions from *T*, using quasi-equivalence. Thus, the set of *d*-*Bounded Quasi-Consistent trees* is denoted by $C_{d}(T)=\{F(T'):T\succeq _{d} T'\}$.

Clearly $C(T)=C_0(T)$, and so in a setting where $d=0$ the former notation is used. For a node *x* of a PQ-tree *T*, the subtree of *T* rooted in *x* is denoted by *T*(*x*), the set of leaves in *T*(*x*) is denoted by $\mathsf {leaves}(x)$, and the *span* of *x* is denoted by $\mathsf {span}(x)$ and defined as $|\mathsf {leaves}(x)|$.

### Defining the problems

As a preface for the new problems defined ahead, consider the PQ-Tree Membership problem defined in Problem 1 below, which stems from the definition of consistent set.

#### Problem 1

(PQ-Tree Membership) Given a PQ-tree *T* and a string *S*, decide if $S\in C(T)$.

When considering applications of PQ-trees to comparative genomics, it is important to allow for insertion, deletion and substitution operations. Thus, a new problem named PQ-Tree Alignment is defined. In what follows we give a decision variant of this problem (in Problem 2), and an optimization variant of this problem (in Problem 3). PQ-Tree Alignment can be thought of as an extension of the PQ-Tree Membership problem that allows insertions, deletions and substitutions of genes. Then, intuitively, given a PQ-tree *T* and a string $S'$, the objective is to find a string $S''$ such that $S''\in C(T)$ and $S''$ is the most similar to $S'$, where similarity is measured as a sequence alignment score. To avoid confusion, the term *insertion* is not used, and instead two types of deletions are used: deletions form the PQ-tree and deletions form the string. In addition, in the rest of this paper, the term *substitution* is used to encompass both matches and mismatches between aligned genes.

Formally, an instance of the PQ-Tree Alignment problem is a tuple of the form $$(T, S', h, d_T, d_S)$$, where *T* is a PQ-tree with *m* leaves, $$m_p$$ P-nodes, $$m_q$$ Q-nodes, and every leaf *x* in *T* has a label $$\mathsf {label}(x)$$$$\in \Sigma _T$$; $$S'=\sigma _1 \cdots \sigma _n \in {\Sigma _S^n}$$ is a string of length *n* representing a sequence of genes; $$d_T\in {\mathbb {N}}$$ specifies the number of allowed deletions from *T*; $$d_S\in {\mathbb {N}}$$ specifies the number of allowed deletions from $$S'$$; and *h* is a *boolean substitution function*, describing the possible substitutions between the leaf labels of *T* and the characters of the given string, $$S'$$. The function *h* receives a pair $$(\sigma _t, \sigma _s)$$, where $$\sigma _t \in \Sigma _T$$ is one of the labels of the leaves of *T*, and $$\sigma _s \in \Sigma _S$$ is one of the characters of the given string $$S'$$, and returns *True* if $$\sigma _t$$ can be replaced with $$\sigma _s$$, and *False*, otherwise. Considering the biological problem at hand, $$\Sigma _T$$ and $$\Sigma _S$$ are both sets of genes.

The objective of PQ-Tree Alignment is to find a one-to-one mapping ${\mathcal {M}}{}$ between the leaves of *T* and the characters of $S'$, which comprises a set of pairs each having one of three forms: the substitution form, $(x,\sigma _s(\ell ))$, where *x* is a leaf in *T*, $\sigma _s \in \Sigma _S$, $h(\mathsf {label}(x),\sigma _s) = True$ and $\ell \in \{1,\cdots {,}n\}$ is the index of the occurrence of $\sigma _s$ in $S'$ that is mapped to the leaf *x*; the character deletion form, $(\varepsilon , \sigma _s(\ell ))$, which marks the deletion of the character $\sigma _s\in \Sigma _S$ at index $\ell$ of $S'$; the leaf deletion form, $(x, \varepsilon )$, which marks the deletion of *x*, a leaf node of *T*.

**Fig. 3:**
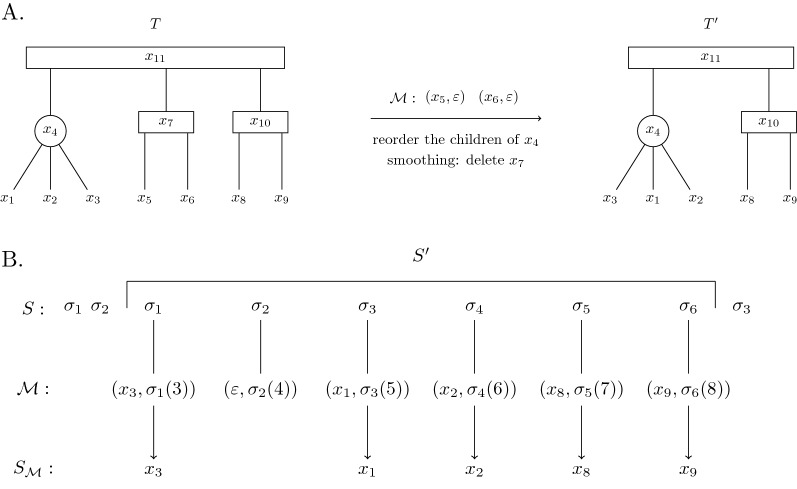
An illustration of the derivation $\mu$ from the PQ-tree *T* to the substring $S'$ of *S*, where $S'=S[3:8]$, under the one-to-one mapping ${\mathcal {M}}{}$ ($\mu .o$) with $\mu .del_T=2$ deletions from the tree and $\mu .del_S=1$ deletions from the string. The start point of the derivation ($\mu .s$) is 3. The end point of the derivation ($\mu .e$) is 8. Notice that $S_{\mathcal {M}}{}=F(T')$ and $T \succeq _2 T'$, which means that $S_{\mathcal {M}}{}\in C_2(T)$. **A** The derivation $\mu$ applied to *T* resulting in $T'$: reorder the children of $x_4$, delete leaves according to ${\mathcal {M}}{}$ (delete $x_5$ and $x_6$) and perform smoothing (delete $x_7$, the parent node of $x_5$ and $x_6$). The root of *T* ($x_{11}$) is the node that $\mu$ derives, denoted $\mu .v$. Also, we say that $\mu$ is a derivation of $x_{11}$. The nodes $x_5$, $x_6$ and $x_7$ are deleted under $\mu$. The leaves $x_1,x_2,x_3,x_8$ and $x_9$ are mapped under $\mu$. The nodes $x_4, x_{10}$ and $x_{11}$ are kept under $\mu$. **B** The derivation $\mu$ applied to $S'$ resulting in $S_{\mathcal {M}}{}$: apply substitutions and deletions according to ${\mathcal {M}}{}$. The substring $S'=S[3:8]$ is the string that $\mu$ derives. The character $S[4]=S'[2]$ is deleted under $\mu$. The characters *S*[3], *S*[5], *S*[6], *S*[7] and *S*[8] are mapped under $\mu$

Applying the substitutions defined in ${\mathcal {M}}{}$ to $S'$, resulting in the string $S_{{\mathcal {M}}{}}$, is the process in which for every $(x,\sigma _s(\ell ))\in {\mathcal {M}}{}$, the character $\sigma _s$ at index $\ell$ of $S'$ is deleted if $x=\varepsilon$, and otherwise substituted by $\mathsf {label}(x)$. This process is demonstrated in Fig. 3B. We say that $S'$ is *derived* from *T* *under* ${\mathcal {M}}{}$ with $d_T$ deletions from the tree and $d_S$ deletions from the string, if $d_T$ is equal to the number of pairs in ${\mathcal {M}}{}$ of the leaf deletion form $(x,\varepsilon )$, $d_S$ is equal to the number of pairs in ${\mathcal {M}}{}$ of the character deletion form $(\varepsilon ,\sigma )$, and $S_{{\mathcal {M}}{}}\in C_{d_T}(T)$. Thus, by definition, there is a PQ-tree $T'$ such that $F(T')=S_{{\mathcal {M}}{}}$ and $T \succeq _{d_T} T'$. Note that the deletions of the nodes in *T* to obtain the nodes in $T'$ are determined by ${\mathcal {M}}{}$. The conversion of *T* to $T'$ as defined by the derivation is illustrated in Fig. 3A. The set of permutations and node deletions performed to obtain $T'$ from *T* together with the substitutions and deletions from $S'$ specified by ${\mathcal {M}}{}$ is named the *derivation* $\mu$ of *T* to $S'$. We also say that ${\mathcal {M}}{}$ *yields* the derivation $\mu$.

#### Problem 2

(Decision PQ-Tree Alignment) Given a string $S'$ of length *n*, a PQ-tree *T* with *m* leaves, deletion bounds $d_T,d_S \in {\mathbb {N}}{}$, and a boolean substitution function *h* between $\Sigma _S$ and $\Sigma _T$, decide if there is a one-to-one mapping ${\mathcal {M}}{}$ that yields a derivation of *T* to $S'$ with up to $d_T$ and up to $d_S$ deletions from *T* and $S'$, respectively.

Notice that by setting both deletion bounds ($d_T$ and $d_S$) to zero and defining $h(\sigma _t, \sigma _s)=True$ if and only if $\sigma _t =\sigma _s$, the PQ-Tree Membership problem is obtained from PQ-Tree Alignment. Also, if $n < m-d_T$ or $n>m+d_S$, then PQ-Tree Alignment will return false.

To define an optimization version of the PQ-Tree Alignment problem it is necessary to have a score for every possible substitution between the characters in $\Sigma _T$ and the characters in $\Sigma _S$. Hence, for this problem variant assume that *h* is a *substitution scoring function*, that is, $h(\sigma _t,\sigma _s)$ for $\sigma _t\in \Sigma _T, \sigma _s\in \Sigma _S$ is the score for substituting $\sigma _s$ by $\sigma _t$ in the derivation, and if $\sigma _t$ cannot be substituted by $\sigma _s$, then $h(\sigma _t,\sigma _s) = -\infty$. In addition, we need a cost function, denoted by $\delta$, for the deletion of a character of $S'$ and for the deletion of a leaf of *T* according to the label of the leaf. So, formally, an instance of the optimization variant of PQ-Tree Alignment is $(T, S', h, \delta , d_T, d_S)$. The score of a derivation $\mu$, denoted by $\mu .score$, is the sum of scores of all operations (deletions from the tree, deletions from the string and substitutions) in $\mu$. Now, instead of deciding whether there exists a one-to-one mapping that yields a derivation of *T* to $S'$, we can search for the one-to-one mapping that yields the best derivation (if there exists such a derivation), i.e. a one-to-one mapping for which $\mu .score$ is the highest.

#### Problem 3

(Optimization PQ-Tree Alignment) Given a string $S'$ of length *n*, a PQ-tree *T* with *m* leaves, deletion bounds $d_T,d_S\in {\mathbb {N}}{}$, a substitution scoring function *h* between $\Sigma _S$ and $\Sigma _T$, and a deletion cost function $\delta$, return the one-to-one mapping ${\mathcal {M}}{}$ that yields the highest scoring derivation of *T* to $S'$ with up to $d_T$ deletions from *T* and up to $d_S$ deletions from $S'$ (if such a mapping exists).

More generally, in our application the string represents a genome, which is a lot longer than the strings that can be derived from the given PQ-tree *T*. Thus, a new problem named PQ-Tree Search is defined (in Problems 4, 5 below). Intuitively, in PQ-Tree Search the objective is to find a substring of a given string *S* for which PQ-Tree Alignment returns true (or returns the best score, in the optimization variant).

Formally, an instance of the PQ-Tree Search problem is a tuple $(T, S, h, d_T, d_S)$, where $T, h, d_T$ and $d_S$ are defined as in PQ-Tree Alignment, and *S* is defined as $S'$ with the exception that the string *S* representing the input genome, can be of any length *n* (and not bounded by $m-d_T$ and $m+d_S$). The objective of PQ-Tree Search is to find a one-to-one mapping ${\mathcal {M}}{}$ between the leaves of *T* and the characters of a substring $S'$ of *S* that yields a derivation with up to $d_T$ and up to $d_S$ deletions from *T* and $S'$, respectively. For $1\le i\le j \le n$, $S'=S[i:j] = \sigma _i...\sigma _j$ is a substring of *S* beginning at index *i* and ending at index *j* (inclusive). The substring $S'$ is a *prefix* of *S* if $S'=S[1:j]$ and it is a *suffix* of *S* if $S'=S[i:n]$. In addition, we denote $\sigma _i$, the $i^{\text {th}}$ character of *S*, by *S*[*i*].

Now, we would like to acknowledge in the definition of a derivation that a derivation can be to a substring of the target string (as it is in the PQ-Tree Search problem), rather than requiring that the full target string is derived, and add some related terms and notations. So, for a derivation $\mu$ of *T* to $S'=S[s:e]$, the following terms and notations (illustrated in Fig. 3) are given. The root of *T* (denoted $root_T$) is *the node that *$\mu$ *derives* or *the root of the derivation* and it is denoted by $\mu .v$. For abbreviation, we say that $\mu$ *is a derivation of* $\mu .v$. The substring $S'$ is *the string that* $\mu$ *derives* . We name *s* and *e* the start and end points of the derivation and denote them by $\mu .s$ and $\mu .e$, respectively. The one-to-one mapping that yields $\mu$ is denoted by $\mu .o$. The number of deletions from the tree is denoted by $\mu .del_T$. The number of deletions from the string is denoted by $\mu .del_S$. In addition, if *x* is a leaf node in *T* and $(x,\sigma _s(\ell ))\in \mu .o$, then *x* is *mapped to* $S[\ell ]$ *under* $\mu$. The character $S[\ell ]$ is said to be *deleted under* $\mu$ if $(\varepsilon ,\sigma _s(\ell ))\in \mu .o$. If $x \in T(\mu .v)$ is a leaf for which $(x,\varepsilon )\in \mu .o$, then *x* is *deleted under* $\mu$. For an internal node *x* of *T*, if every leaf in *T*(*x*) is deleted under $\mu$, then *x* is *deleted under* $\mu$, and otherwise *x* is *kept under* $\mu$. Notice that in PQ-Tree Alignment all the derivations have the start point 1 ($s=1$) and the end point *m* ($e=m$). Given a node *x* and the numbers of deletions $k_T$ and $k_S$ of a derivation, the length of the derived string $S'$ can be calculated using the following length function: $L(x,k_T,k_S) \doteq \mathsf {span}(x) - k_T + k_S$.

We define two versions of the PQ-Tree Search problem: a decision version (Problem 4) and an optimisation version (Problem 5).

#### Problem 4

(Decision PQ-Tree Search) Given a string *S* of length *n*, a PQ-tree *T* with *m* leaves, deletion bounds $d_T,d_S \in {\mathbb {N}}{}$, and a boolean substitution function *h* between $\Sigma _S$ and $\Sigma _T$, decide if there is a one-to-one mapping ${\mathcal {M}}{}$ that yields a derivation of *T* to a substring $S'$ of *S* with up to $d_T$ and up to $d_S$ deletions from *T* and $S'$, respectively.

#### Problem 5

Given a string *S* of length *n*, a PQ-tree *T* with *m* leaves, deletion bounds $d_T,d_S\in {\mathbb {N}}{}$, a substitution scoring function *h* between $\Sigma _S$ and $\Sigma _T$, and a deletion cost function $\delta$, return the one-to-one mapping ${\mathcal {M}}{}$ that yields the highest scoring derivation of *T* to a substring $S'$ of *S* with up to $d_T$ deletions from *T* and up to $d_S$ deletions from $S'$ (if such a mapping exists).

## A parameterized algorithm

In this section we develop a dynamic programming (DP) algorithm to solve the optimization variant of PQ-Tree Search (Problem 5). Our algorithm receives as input an instance of PQ-Tree Search $(T, S, h, d_T, d_S)$, where *h* is a substitution scoring function. Our default assumption is that deletions are not penalized, and therefore $\delta$ (the deletion cost function) is not given as input. The case where deletions are penalized is described in Sect. 2 of Additional file 1. The output of the algorithm is a one-to-one mapping, ${\mathcal {M}}{}$, that yields the best (highest scoring) derivation of *T* to a substring of *S* with up to $d_T$ deletions from *T* and up to $d_S$ deletions from the substring, and the score of that derivation. With a minor modification, the output can be extended to include a one-to-one mapping for every substring of *S* and the derivations that they yield.

### Brief overview

**Fig. 4:**
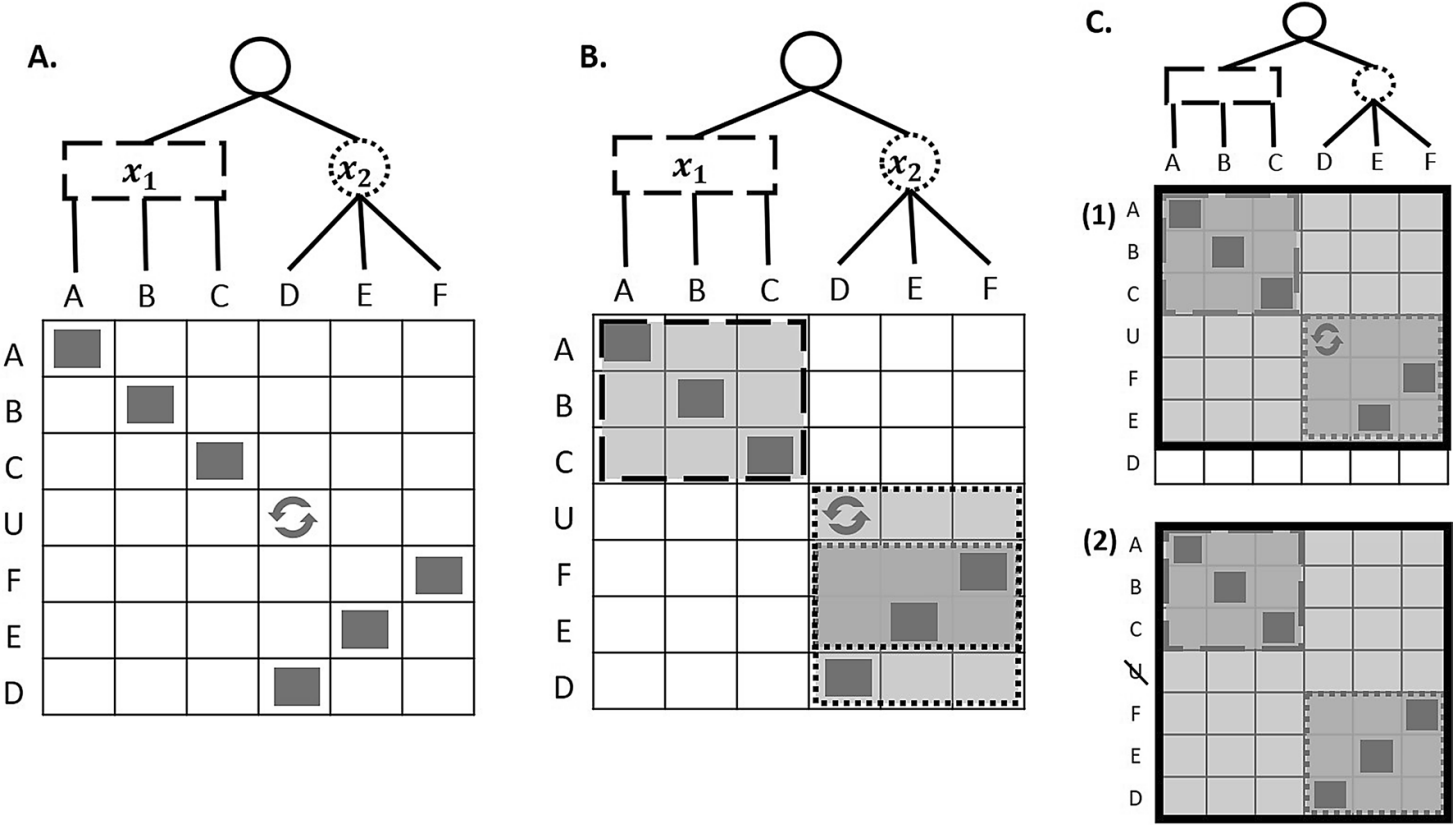
The outline of the algorithm that solves PQ-Tree Search. **A** During initialization the best derivations of the leaves of *T* are computed. The cell with two arrows marks the substitution between D and U. **B** PQ-Tree Alignment is solved for each substring of the target string *S* and the subtrees rooted in each internal node of *T*. **C** In this example, there are two derivations from the root of the PQ-tree; One is to the substring from index 1 to 6 and the other is to the entire string (if the character U is deleted from the string)

On a high level, our algorithm consists of three components: the main algorithm, and two other algorithms that are used as procedures by the main algorithm. Apart from an initialization phase, the crux of the main algorithm is a loop that traverses the given PQ-tree, *T*. For each internal node *x*, it calls one of the two other algorithms: P-mapping (given in " P-node mapping: the algorithm" section) and Q-mapping. These algorithms find and return the best derivations from the subtree of *T* rooted in *x*, *T*(*x*), to substrings of *S*, based on the type of *x* (P-node or Q-node). So, the main algorithm solves PQ-Tree Alignment for all substrings of *S* that start at a specific index. Then, the scores of the derivations are stored in the DP table. The outline of the algorithm is exemplified in Fig. 4.

We now give a brief informal description of the main ideas behind our P-mapping and Q-mapping algorithms. Our P-mapping algorithm is inspired by an algorithm described by van Bevern et al. [43] to solve the Job Interval Selection problem. Our problem differs from theirs mainly in its control of deletions. Intuitively, in the P-mapping algorithm we consider the task at hand as a packing problem, where every child of *x* is a set of intervals, each corresponding to a different substring. The objective is to pack non-overlapping intervals such that for every child of *x* at most one interval is packed. Then, the algorithm greedily selects a child $x'$ of *x* and decides either to pack one of its intervals (and which one) or to pack none (in which case $x'$ is deleted). The Q-mapping algorithm is similar to the classical problem of sequence alignment with bounded gaps and therefore will not be elaborated in the paper. It is deferred to the supplementary material (see Sect. 1, Additional file 1).

In the following sections, we describe the main algorithm ("The main algorithm" section) and the P-mapping algorithm ("P-node mapping" section). Afterwards, the time complexity of the algorithm is analyzed and compared to that of a naïve algorithm ("Complexity analysis of the PQ-tree search aalgorithm" section). The modifications necessary for penalizing deletions are deferred to the supplementary material (see Sect. 2, Additional file 1).

### The main algorithm

We now delve into more technical details. The algorithm (whose pseudocode is given in 1) constructs a 4-dimensional DP table ${\mathcal {A}}{}$ of size $m' \times n \times d_T+1 \times d_S+1$, where $m'=m+m_p+m_q$ is the number of nodes in *T*. The purpose of an entry of the DP table, ${\mathcal {A}}[x,i,k_T,k_S]$, is to hold the highest score of a derivation of the subtree *T*(*x*) to a substring $S'$ of *S* starting at index *i* with $k_T$ deletions from *T*(*x*) and $k_S$ deletions from $S'$. Note that we abuse notation and use a node *x* of *T* also as an index for the DP table entries that refer to *x*. If no such derivation exists, ${\mathcal {A}}[x,i,k_T,k_S] = -\infty$. Addressing ${\mathcal {A}}{}$ with some of its indices given as dots, e.g. ${\mathcal {A}}[x, i,\cdot ,\cdot ]$, refers to the subtable of ${\mathcal {A}}{}$ that is comprised of all entries of ${\mathcal {A}}{}$ whose first two indices are *x* and *i*. Some entries of the DP table define illegal derivations, namely, derivations for which the number of deletions are inconsistent with the start index *i*, the derived node and *S*. For example, such are derivations that have more deletions from the string than there are characters in the derived string. These entries are called *invalid entries* and their value is defined as $-\infty$ throughout the algorithm. Formally, an entry ${\mathcal {A}}[x,i,k_T,k_S]$ is invalid if one of the following is true: $k_T > \mathsf {span}(x)$, $k_S > L(x,k_T,k_S)$, $E(x,i,k_S,k_T) > n$, or $L(x,k_T,k_S) < 0$.

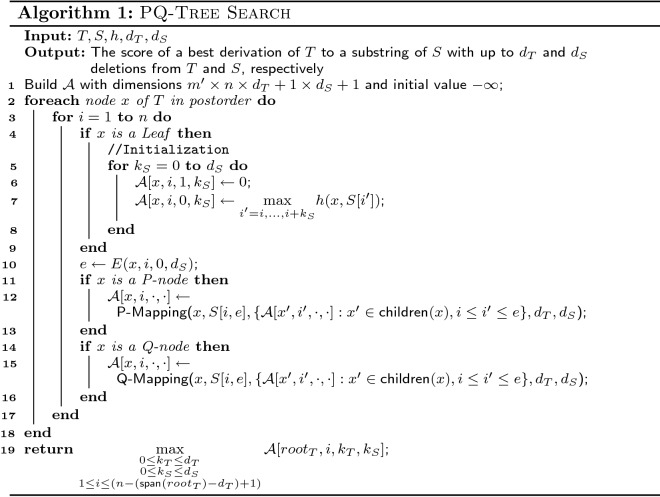



The algorithm first initializes the entries of ${\mathcal {A}}{}$ that are meant to hold scores of derivations of the leaves of *T* to every possible substring of *S* using the following rule. For every $0\le k_S\le d_S$ and every $x\in \mathsf {leaves}(root_T)$, do:

1. $${\mathcal {A}}[x,i,1,k_S] = 0$$
2. $${\mathcal {A}}[x,i,0,k_S] = \displaystyle {\max _{\begin{array}{c} i'=i,...,i+k_S \end{array}}}h(x,S[i'])$$

We remark that this initialization rule can be replaced by initializing ${\mathcal {A}}[x,i,0,0]$ with *h*(*x*, *S*[*i*]) and for every $k_T\ne 0$ and $k_S\ne 0$ initializing ${\mathcal {A}}[x,i,k_T,k_S]$ with $-\infty$. Nonetheless, we use the former initialization rule because it does not change the time complexity of the algorithm while helping keep notations and proofs simpler.

After the initialization, all other entries of ${\mathcal {A}}{}$ are filled as follows. Go over the internal nodes of *T* in postorder. For every internal node *x*, go in ascending order over every index *i*, that can be a start index for the substring of *S* derived from *T*(*x*) (the possible values of *i* are explained in the next paragraph). For every *x* and *i*, use the algorithm for Q-mapping or P-mapping according to the type of *x*. Both algorithms receive the same input: a substring $S'$ of *S*, the node *x*, its children $x_{1},\dots ,x_{\gamma }$, the collection of possible derivations of the children (denoted by ${\mathcal {D}}{}$), which have already been computed and stored in ${\mathcal {A}}{}$ (as will be explained ahead) and the deletion arguments $d_T,d_S$. Intuitively, the substring $S'$ is the longest substring of *S* starting at index *i* that can be derived from *T*(*x*) given $d_T$ and $d_S$. After being called, both algorithms return a set of derivations of *T*(*x*) to a prefix of $S'=S[i:e]$ and their scores. The set holds the highest scoring derivation for every $E(x,i,d_T,0) \le e \le E(x,i,0,d_S)$ and for every legal deletion combination $0\le k_T\le d_T$, $0\le k_S \le d_S$.

Next, we explain the possible values of *i* and the definition of $S'$ more formally. To this end, recall the length function given in "Preliminaries" section, $L(x,k_T,k_S) \doteq \mathsf {span}(x) - k_T + k_S$. Thus, on the one hand, a substring of maximum length is obtained when there are no deletions from the tree and $d_S$ deletions from the string. Hence, $S'=S[i:E(x,i,0,d_S)]$. On the other hand, a shortest substring is obtained when there are $d_T$ deletions from the tree and none from the string. Then, the length of the substring is $L(x,d_T,0) = \mathsf {span}(x)-d_T$. Hence, the index *i* runs between 1 and $n-(\mathsf {span}(x)-d_T)+1$.

We now turn to address the aforementioned input collection ${\mathcal {D}}{}$ in more detail. Formally, it contains the best scoring derivations of every child $x'$ of *x* to every substring of $S'$ with up to $d_T$ and $d_S$ deletions from the tree and string, respectively. It is produced from the entries ${\mathcal {A}}[x', i', k_T, k_S]$ (where each entry gives one derivation) for all $k_T$ and $k_S$, and all $i'$ between *i* and the end index of $S'$, i.e. $i\le i' \le E(x,i,0,d_S)$. For the efficiency of the Q-mapping and P-mapping algorithms, the derivations in ${\mathcal {D}}{}$ are grouped by their root ($\mu .v$) and arranged in descending order with respect to their end point ($\mu .e$). This does not increase the time complexity of the algorithm, as this ordering is received by previous calls to the Q-mapping and P-mapping algorithms.

In the final stage of the main algorithm, when the DP table is full, the score of a best derivation is the maximum of $\{{\mathcal {A}}[root_T,i,k_T,k_S] : k_T\le d_T$, $k_S \le d_S$, $1\le i\le n-(\mathsf {span}(root_T)-k_T)+1\}$. We remark that by tracing back through ${\mathcal {A}}{}$ the one-to-one mapping that yielded this derivation can be found.

### P-node mapping

Before describing the P-mapping algorithm, we set up some useful terminology.

#### P-node mapping: terminology

We first define the notion of a *partial derivation*. In the P-mapping algorithm, the derivation of the input node *x* is built by considering subsets *U* of its children. With respect to such a subset *U*, a derivation $\mu$ of *x* is built as if *x* had only the children in *U*, and is called a *partial derivation*. Formally, $\mu$ is a partial derivation of a node *x* if $\mu .v=x$ and there is a subset of children $U'\subseteq \mathsf {children}(x)$ such that the two following conditions are true. First, for every $u\in U'$ all the leaves in *T*(*u*) are neither mapped nor deleted under $\mu$ - that is, there is no mapping pair $(\ell ,y) \in \mu .o$ such that $\ell \in \mathsf {leaves}(u)$. Second, for every $v \in \mathsf {children}(x)\setminus U'$ the leaves in *T*(*v*) are either mapped or deleted under $\mu$. For every $u \in U'$, we say that *u* is *ignored under* $\mu$. Notice that any derivation is a partial derivation, where the set of ignored nodes ($U'$ above) is empty. Since all derivations that are computed in a single call to the P-mapping algorithm have the same start point *i*, it can be omitted (for brevity) from the end point function: thus, we denote $E_{I}(x,k_T,k_S)\doteq L(x,k_T,k_S)$. Also, for a set *U* of nodes, we define $L(U,k_T,k_S) \doteq \sum _{x\in U}\mathsf {span}(x)+k_S-k_T$ and accordingly $E_{I}(U,k_T,k_S)\doteq L(U,k_T,k_S)$.

We now define certain collections of derivations with common properties (such as having the same numbers of deletions and end point).

##### Definition 2

The collection of all the derivations of every node $u \in U$ to suffixes of $S'[1:E_{I}(U,k_T,k_S)]$ with *exactly* $k_T$ deletions from the tree and *exactly* $k_S$ deletions from the string is denoted by ${\mathcal {D}}{(U,k_T,k_S)}$.

##### Definition 3

The collection of all the best derivations from the nodes in *U* to suffixes of $S'[1:E_{I}(U,k_T,k_S)]$ with *up to* $k_T$ deletions from the tree and *up to* $k_S$ deletions from the string is denoted by ${\mathcal {D}}_\le {(U,k_T,k_S)}$. Specifically, for every node $u \in U$, $k'_T\le k_T$ and $k'_S\le k_S$, the set ${\mathcal {D}}_\le {(U,k_T,k_S)}$ holds only one highest scoring derivation of *u* to a suffix of $S'[1:E_{I}(U,k_T,k_S)]$ with $k'_T$ and $k'_S$ deletions from the tree and string, respectively.

It is important to distinguish between these two definitions. First, the derivations in ${\mathcal {D}}{(U,k_T,k_S)}$ have *exactly* $k_T$ and $k_S$ deletions, while the derivations in ${\mathcal {D}}_\le {(U,k_T,k_S)}$ have *up to* $k_T$ and $k_S$ deletions. Second, in ${\mathcal {D}}{(U,k_T,k_S)}$ there can be several derivations that differ only in their score and in the one-to-one mapping that yields them, while in ${\mathcal {D}}_\le {(U,k_T,k_S)}$, there is only one derivation for every node $u\in U$ and deletion combination pair $(k'_T,k'_S)$. Note that the end points of all of the derivations are equal.

Definition 2 is used for describing the content of an entry of the DP table, where the focus is on the collection of all the derivations of *x* to $S'$ with exactly $k_T$ and $k_S$ deletions, ${\mathcal {D}}{(\{x\},k_T,k_S)}$. For simplicity, the abbreviation ${\mathcal {D}}{(u,k_T,k_S)} = {\mathcal {D}}{(\{u\},k_T,k_S)}$ is used. In every step of the P-mapping algorithm, a different set of derivations of the children of *x* is examined, thus, Definition 3 is used for $U \subseteq \mathsf {children}(x)$. In addition, the set of derivations ${\mathcal {D}}{}$ that is received as input to the algorithms can be described using Definition 3 as can be seen in Eq. 1 below. In this equation, the union is over all $U\subseteq \mathsf {children}(x)$ because in this way the derivations of all the children of *x* with *every possible end point* are obtained (in contrast to having only $U=\mathsf {children}(x)$, which results in the derivations of all the children of *x* with the end point $E_{I}(\mathsf {children}(x),k_T,k_S)$).

1 $$\begin{aligned} {\mathcal {D}}{} = \bigcup _{U \subseteq \mathsf {children}(x)}\bigcup _{k_T\le d_T}\bigcup _{k_S\le d_S} {\mathcal {D}}_\le {(U,k_T,k_S)} \end{aligned}$$

In the P-mapping algorithm for $C \subseteq \mathsf {children}(x)$, the notation $x^{(C )}$ is used to indicate that the node *x* is considered as if its only children are the nodes in *C* (the nodes in $\mathsf {children}(x)\setminus C$ are ignored). Consequentially, the span of $x^{(C )}$ is defined as $\mathsf {span}(x^{(C )}) \doteq \sum _{c\in C}\mathsf {span}(c)$, and the set ${\mathcal {D}}(x^{(C )},k_T,k_S){}$ (in Definition 2 where $U=\{x^{(C )}\}$) now refers to a set of *partial* derivations. To use $x^{(C )}$ to describe the base cases of the algorithm, let us define $x^{(\emptyset )}$ ($x^{(C )}$ for $C=\emptyset$) as a tree with no labeled leaves to map.

#### P-node mapping: the algorithm

Recall that the input consists of an internal P-node *x*, a string $S'$, bounds on the number of deletions from the tree *T* and the string $S'$, $d_T$ and $d_S$, respectively, and a set of derivations ${\mathcal {D}}{}$ (see Eq. 1). The output of the algorithm is $\bigcup _{0\le k_T \le d_T}\bigcup _{0\le k_S \le d_S}{{\,\mathrm{arg\max }\,}}_{\mu \in {\mathcal {D}}(x,k_T,k_S){}}\mu .score$, which is the collection of the best scoring derivations of *x* to every possible prefix of $S'$ having up to $d_T$ and $d_S$ deletions from the tree and string, respectively. Thus, there are $O(d_T d_S)$ derivations in the output.

The algorithm (whose pseudocode is given in 2) constructs a 3-dimensional DP table ${\mathcal {P}}{}$, which has an entry for every $0\le k_T \le d_T$, $0\le k_S \le d_S$ and subset $C\subseteq \mathsf {children}(x)$. The purpose of an entry ${\mathcal {P}}[C,k_T,k_S]$ is to hold the best score of a partial derivation in ${\mathcal {D}}(x^{(C )},k_T,k_S){}$, i.e. a partial derivation rooted in $x^{(C )}$ to a prefix of $S'$ with exactly $k_T$ deletions from the tree and $k_S$ deletions from the string. The children of *x* that are not in *C* are *ignored* (as defined in "P-node mapping: terminology" section) under the partial derivation stored by the DP table entry ${\mathcal {P}}[C,k_T,k_S]$, thus they are neither deleted nor counted in the number of deletions from the tree, $k_T$. (They will be accounted for in the computation of other entries of ${\mathcal {P}}{}$.) Similarly to the main algorithm, some of the entries of ${\mathcal {P}}{}$ are invalid, and their value is defined as $-\infty$. Formally, an entry ${\mathcal {P}}[C,k_T,k_S]$ is invalid if one of the following is true: $k_T > \sum _{c \in C}\mathsf {span}(c)$, $k_S > L(x^{(C )},k_T,k_S)$, $L(x^{(C )},k_T,k_S) > \mathsf {len}(S')$, or $L(x^{(C )},k_T,k_S) < 0$. Every entry ${\mathcal {P}}[C,k_T,k_S]$ for which $L(x^{(C )},k_T,k_S)=0$ and $k_S=0$ or for which $C=\emptyset$ and $k_T=0$ is initialized with 0. The first set of entries captures the case in which the derived substring is the empty string and thus no character can be deleted from it, i.e. $k_S$ must equal 0. The second set of entries captures the case in which all the children of *x* are ignored, thus the value of $k_T$ must be 0.

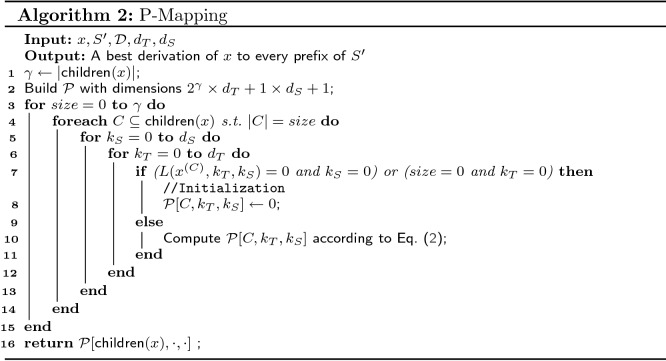



After the initialization, the remaining entries of ${\mathcal {P}}{}$ are calculated using the recursion rule in Eq. 2 below. The order of computation is ascending with respect to the size of the subsets *C* of the children of *x*, and for a given $C\subseteq \mathsf {children}(x)$, the order is ascending with respect to the number of deletions from both tree and string.

2 $$\begin{aligned} {\mathcal {P}}[C,k_T,k_S] = \max {\left\{ \begin{array}{ll} {\mathcal {P}}[C,k_T,k_S-1] \\ \displaystyle \max _{\mu \in {\mathcal {D}}_\le (C,k_T,k_S)} {\mathcal {P}}[C\setminus \{\mu .v\},k_T-\mu .del_T,k_S-\mu .del_S] + \mu .score \end{array}\right. } \end{aligned}$$

Intuitively, every entry ${\mathcal {P}}[C,k_T,k_S]$ defines some index $e'$ of $S'$ that is the end point of every partial derivation in ${\mathcal {D}}(x^{(C )},k_T,k_S){}$. Thus, $S'[e']$ must be a part of any partial derivation $\mu \in {\mathcal {D}}(x^{(C )},k_T,k_S){}$, so, either $S'[e']$ is deleted under $\mu$ or it is mapped under $\mu$. The former option is captured by the first case of the recursion rule. If $S'[e']$ is mapped under $\mu$, then due to the hierarchical structure of *T*(*x*), it must be mapped under some derivation $\mu '$ of one of the children of *x* that are in *C*. Thus, we receive the second case of the recursion rule.

We remark that the case of a node deletion is captured by the initialization and that adding the option of deleting a node in the recursion rule is therefore redundant.

Once the entire DP table is filled, a derivation of maximum score for every end point and deletion numbers combination can be found in ${\mathcal {P}}[\mathsf {children}(x), \cdot , \cdot ]$. Traversing the said subtable in a specific order guarantees the output derivations are ordered with respect to their end point without further calculations.

### Complexity analysis of the PQ-Tree Search algorithm

In this section we compare the time complexity of the main algorithm (in "The main algorithm" section) to the naïve solution for PQ-Tree Search. The complexities of the two algorithms described before as well as the complexity of the Q-mapping algorithm are given in the followings lemmas. Lemma 1 and Lemma 2 are proven in "Time and space complexity of the PQ-tree search algorithm" section, and Lemma 3 is proven in Sect. 1.3 of Additional file 1.

#### Lemma 1

The algorithm in "The main algorithm" section takes $O(n \gamma {d_T}^2 {d_S}^2 (m_p 2^{\gamma } + m_q))$ time and $O(d_T d_S (m n + 2^\gamma ))$ space, where $\gamma$ is the maximum degree of a node in *T*.

#### Lemma 2

The P-mapping algorithm takes $O({d_T}^2 {d_S}^2 \gamma 2^\gamma )$ time and $O(d_T d_S 2^\gamma )$ space.

#### Lemma 3

The Q-mapping algorithm takes $O({d_T}^2 {d_S}^2 \gamma )$ time and $O(d_T d_S \gamma )$ space.

From Lemma 1 it is proven that PQ-Tree Search has an FPT solution with the parameter $\gamma$ (Theorem 1).

#### Theorem 1

PQ-Tree Search with parameter $\gamma$ is FPT. Particularly, it has an FPT algorithm that runs in $O^*(2^{\gamma })$ time^1^.

The naïve solution for PQ-Tree Search is to solve sequence alignment with bounded gaps for every substring of *S* versus every string $S'\in C(T)$, so the naïve solution takes $O(2^{m_q}{(\gamma !)}^{m_p} n m(d_T+d_S)d_T d_S)$ time. (A full description of the naïve algorithm and its complexity is given in Sect. 4 of Additional file 1.) Therefore, we conclude that the time complexity of our algorithm is substantially better, as exemplified by considering two complementary cases. One, when there are only P-nodes in *T* (i.e. $m_p=O(m)$), the naïve algorithm is super-exponential in $\gamma$, and even worse, exponential in *m*, while ours is exponential only in $\gamma$, and hence polynomial for any $\gamma$ that is constant (or even logarithmic in the input size). Second, when there are only Q-nodes in *T* (i.e. $m_q=O(m)$), the naïve algorithm is exponential while ours is polynomial.

## Methods and datasets

**Dataset and gene cluster generation.** 1487 fully sequenced prokaryotic strains with COG ID annotations (see Additional file 2) were downloaded from GenBank (NCBI; ver 10/2012). Among these strains, 471 genomes included a total of 933 plasmids.

The gene clusters were generated using the tool CSBFinder-S [44]. CSBFinder-S was applied to all the genomes in the dataset after removing their plasmids, using parameters $q=1$ (a colinear gene cluster is required to appear in at least one genome) and $k=0$ (no insertions are allowed in a colinear gene cluster), resulting in 595,708 colinear gene clusters. Next, ignoring strand and gene order information, colinear gene clusters that contain the exact same COGs were united to form the generalized set of gene clusters. The resulting gene clusters were then filtered to 26,270 gene clusters that appear in more than 30 genomes.

**Generation of PQ-Trees.** The generation of PQ-trees was performed using a program [45] that implements the algorithm described in [28] for the construction of a PQ-tree from a list of strings comprised from the same set of characters. In the case where a character appeared more than once in a training string, the PQ-tree with a minimum sized consistent set was chosen. The generated PQ-trees varied in size and complexity. The length of their frontier ranged between 4 and 31, and the size of their consistent set ranged between 4 and 362, 880.

**Implementation and performance.** PQFinder is implemented in Java 1.8. The runs were performed on an Intel Xeon X5680 machine with 192 GB RAM. The time it took to run all plasmid genomes against one PQ-tree ranged between 5.85 seconds (for a PQ-tree with a consistent set of size 4) and 181.5 seconds (for a PQ-tree with a consistent set of size 362, 880). In total it took an hour and 47 minutes to run each one of the 779 PQ-trees against each one of the 933 plasmids.

**Substitution scoring function.** The substitution scoring function reflects the distance between each pair of COGs, that is computed based on sentences describing the functional annotation of the COGs (e.g., “ABC-type sugar transport system, ATPase component”). The “Bag of Words model” was employed, where the functional description of each COG is represented by a sparse vector that is normalized to have a unit Euclidean norm. First, each COG description was tokenized and the occurrences of tokens in each description was counted and normalized using tf-idf term weighting. Then, the cosine similarity between each two vectors was computed, resulting in similarity scores ranging between 0 and 1. The sentences describing COGs are short, therefore each word largely influences the score, even after the tf-idf term weighting. Therefore, words that do not describe protein functions that were found in the top 30 most common words in the description of all COGs were used as stop-words. Two COGs with the same COG IDs were set to have a score of 1.1, and the substitution score between a gene with no COG annotation to any other COG was set to be − 0.1. Two COGs with a zero score were penalized to have a score of -0.2 and the deletion of a COG from the query PQ-tree or the target string was set to have a cost of zero.

**Fig. 5:**
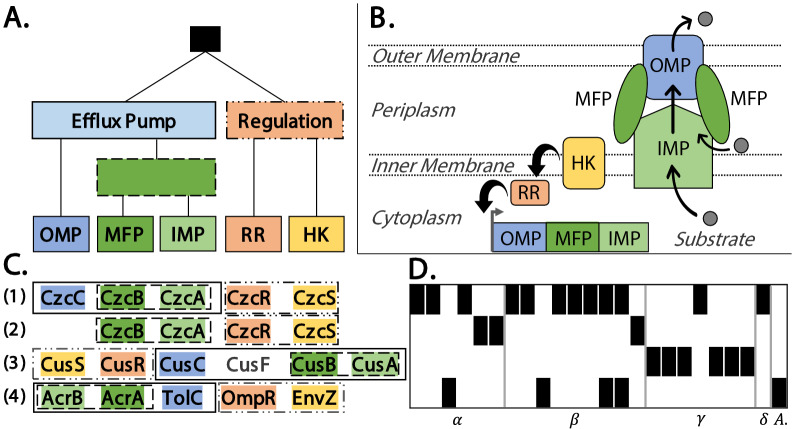
**A** A PQ-tree of a heavy metal RND efflux pump, corresponding to the third top scoring result in Table 1. **B** An illustration of an RND efflux pump consisting of an outer-membrane protein (OMP), an inner membrane protein (IMP), and a periplasmic membrane fusion protein (MFP) that connects the other two proteins. In addition, a two-component regulatory system consisting of a sensor/histidine kinase (HK) and response regulator (RR) enhances the transcription of the efflux pump genes. **C** Representative gene sequences of the three different RND efflux pumps found in plasmids (additional gene insertions and deletions may be present among the various instances of these pumps.) (1) A Czc-like heavy metal efflux pump, (2) A Czc-like heavy metal efflux pump with a missing OMP gene, (3) A Cus-like heavy metal efflux pump, exemplifying an inserted gene (CusF), (4) An Acr-like multidrug efflux pump. Additional details can be found in the text. **D** The presence-absence map of the three types of efflux pumps found in the plasmids of different genomes. The rows correspond to the rows in **C**, the columns correspond to the genomes in which instances were found, organized according to their taxonomic classes. A black cell indicates that the corresponding efflux pump is present in the plasmids of the genome. The labels below the map indicate the classes $\alpha ,\beta ,\gamma ,\delta$-Proteobacteria and Acidobacteriia

**Enrichment analysis.** For each of the four variants in Fig. 5C, a hypergeometric test was performed to measure the enrichment of the corresponding variant in one of the classes in which it appears. A total of 10 p-values were computed and adjusted using the Bonferroni correction; two p-values were found significant ($<0.05$), reported in "Results" section.

**Specificity scor﻿e.** We define a specificity score for a PQ-tree *T* of a gene cluster named S-score, where a more specific tree yields a higher S-score. Let ${\tilde{T}}$ be the least specific PQ-tree that could have been generated for the genes of the gene cluster based on which *T* was constructed. Namely, a PQ-tree that allows all permutations of said genes, has height 1 and is rooted in a P-node whose children (being the leaves of ${\tilde{T}}$) are the leaves of *T*. The S-score of *T* is defined as $\frac{|C({\tilde{T}})|}{|C(T)|}$. For a gene cluster of permutations (i.e. there are no duplications), the computation of |*C*(*T*)| is as described in Eq. 3, where the set of P-nodes in *T* is denoted by *T*.*p*.

3 $$\begin{aligned} |C(T)| = 2^{m_q}\cdot \prod _{x\in T.p}{|\mathsf {children}(x)|!} \end{aligned}$$

For a gene cluster that has duplications, the set *C*(*T*) is generated to learn its size. Let ${\mathsf {a}}(\ell ,T)$ denote the number of appearances of the label $\ell$ in the leaves of *T* and let $\mathsf {labels}(T)$ denote the set of all the distinct labels of the leaves of *T*. So, the formula for $|C({\tilde{T}})|$ is as in Eq. 4. Clearly, for *T* with no duplications $|C({\tilde{T}})| = |F(T)|!$.

4 $$\begin{aligned} |C({\tilde{T}})| = \frac{|F(T)|!}{\prod _{\ell \in \mathsf {labels}(T)}{{\mathsf {a}}(\ell ,T)!}} \end{aligned}$$

## Results

### Chromosomal gene orders rearranged in plasmids

The labeling of each internal node of a PQ-tree as P or Q, is learned during the construction of the tree, based on some interrogation of the gene orders from which the PQ-tree is trained [28]. As a result, the set of strings that can be derived from a PQ-tree *T*, consists of two parts: (1) all the strings representing the known gene orders from which *T* was constructed, and (2) additional strings, denoted *tree-guided rearrangements*, that do not appear in the set of gene orders constructing *T*, but can be obtained via rearrangement operations that are constrained by *T*. Thus, the tree-guided rearrangements conserve the internal topology properties of the gene cluster, as learned from the corresponding gene orders during the construction of *T*, such that colinear dependencies among genes and between sub-operons are preserved in the inferred gene orders.

In this section, we used the PQ-trees constructed from chromosomal gene clusters, to examine whether tree-guided rearrangements can be found in plasmids. The objective was to discover gene orders in plasmids that abide by a PQ-tree representing a chromosomal gene cluster, and differ from all the gene orders participating in the construction of the PQ-tree. PQ-trees that are constructed from gene clusters that have only one gene order or gene clusters with less than four COGs cannot generate gene orders that differ from the ones participating in their construction. Therefore, only 779 out of 26,270 chromosomal gene clusters were used for the construction of query PQ-trees (the generation of the chromosomal gene clusters is detailed in Sect. Methods and datasets). Using our tool PQFinder that implements the algorithm proposed for solving the PQ-Tree Search problem, the query PQ-trees were run against all plasmid genomes. This benchmark was run conservatively without allowing substitutions or deletions from the PQ-tree or from the target string. 380 of the query gene clusters were found in at least one plasmid. The instances of these gene clusters in plasmids are provided in the Supplementary Materials in [2] as a session file that can be viewed using the tool CSBFinder-S [44].

Tree-guided rearrangements were found among instances of 29 chromosomal gene clusters. The PQ-trees corresponding to these gene clusters were sorted by a decreasing S-score, where higher scores are given to a more specific tree (details in "Methods and datasets" section). In this setting, the higher the S-score, the smaller the number of possible gene orders that can be derived from the respective PQ-tree. Interestingly, 21 out of these 29 gene clusters code for transporters, namely 20 importers (ABC-type transport systems) and one exporter (efflux pump). The 10 top ranking results are presented in Table 1.

We selected the third top-ranking PQ-tree in Table 1 for further analysis. This PQ-tree was constructed from seven gene orders of a gene cluster that encodes a heavy metal efflux pump. This gene cluster was found in the chromosomes of 79 genomes (represented by the seven distinct gene orders mentioned above) and in the plasmids of seven genomes. The instance of the chromosomal gene cluster identified as a tree-guided rearrangement in plasmids was found in the strain *Cupriavidus metallidurans CH34*, isolated from an environment polluted with high concentrations of several heavy metals. This strain contains two large plasmids that confer resistance to a large number of heavy metals such as zinc, cadmium, copper, cobalt, lead, mercury, nickel and chromium. We hypothesize that the rearrangement event could have been caused by a heavy metal stress [46]. In the following section we will focus on this PQ-tree to further study its different variants in plasmids.

**Table 1:** Top ranking PQ-trees for which tree-guided rearrangements were found in plasmids

	PQ-Tree^a^	S-score	# Genomes^b^	Functional Category
1	[[0683 [[0411 0410] [0559 4177]]] 0583]	22.5	5 (2)	Amino acid transport
2	(1609 [1653 1175 0395] 3839)	10.0	10 (2)	Carbohydrate transport
3	[[1538 [3696 0845]] [0642 0745]]	7.5	7 (1)	Heavy metal efflux
4	[[2115 1070] [4213 [1129 4214]]]	7.5	1 (1)	Carbohydrate transport
5	[1960 [[2011 1135] [2141 1464]]]	7.5	3 (1)	Amino acid transport
6	[[0596 0599] [[3485 3485] 0015]]	7.5	9 (1)	Metabolism
7	[[[1129 1172 1172] 1879] 3254]	7.5	6 (1)	Carbohydrate transport
8	(1609 1869 [[1129 1172] 1879] 0524)	7.5	1 (1)	Carbohydrate transport
9	(0683 [0559 4177] [0411 0410] 0318)	7.5	1 (1)	Amino acid transport
10	(3839 0673 [[0395 1175] 1653])	5.0	10 (1)	Carbohydrate transport

^a^Square brackets represent a Q-node; round brackets represent a P-node. Numbers indicate the respective COG IDs

^b^This column indicates the number of genomes harboring plasmid instances of the respective PQ-tree. The number in brackets indicates the number of genomes harboring a tree-guided gene rearrangement of the corresponding gene cluster. The full table can be found in the supplementary material (see Additional file 1: Table S1)

### Finding approximate instances of an RND efflux pump

**Fig. 6:**
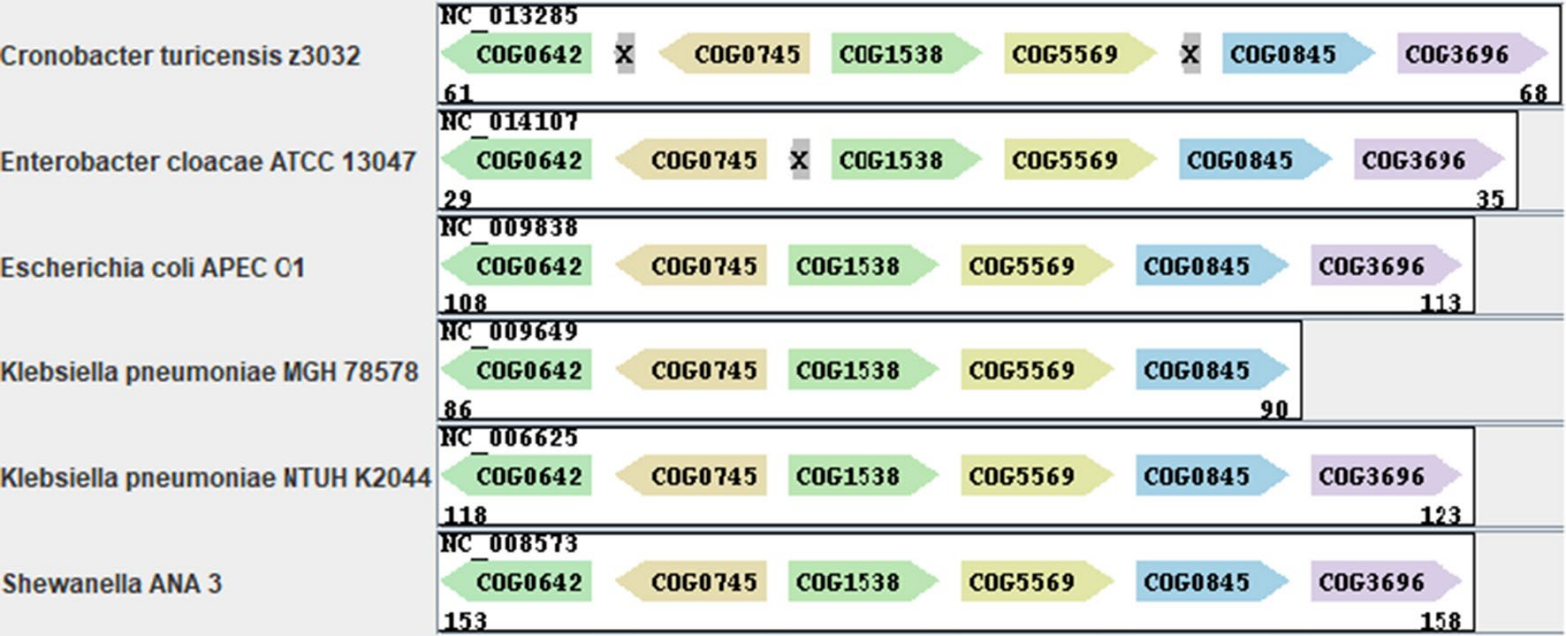
Approximate plasmid instances of the query PQ-tree in Fig. 5A that include an insertion of the gene CusF (COG5569), as detected by PQFinder. These instances correspond to representative (3) from Fig. 5C (CusS-CusR-CusC-CusF-CusB-CusA). The instances are displayed using the graphical interface of the tool CSBFinder-S [44]. COG-to-gene mapping: COG0642:CusS, COG0745:CusR, COG1538:CusC, COG5569:CusF, COG0845:CusB, COG3969:CusA. “X” indicates a gene with no COG annotation. Note that the DNA sequence of *Klebsiella pneumoniae* MGH 78578 was updated by the NCBI on Oct 9, 2019, and since then the gene CusA (COG3696) is identified in the corresponding plasmid (Accession ID NC_009649). Instances of additional variants of this gene cluster query can be found in Additional file 1: Figure S1

The heavy metal efflux pump examined in the previous section (corresponding to the third top-ranking PQ-tree in Table 1), was used as a PQFinder query and re-run against all the plasmids in our dataset in order to discover approximate instances of this gene cluster, possibly encoding remotely related variations of the efflux pump it encodes. This time, in order to increase sensitivity, a semantic substitution scoring function (described in Sect. Methods and datasets) was used, and the arguments were set to $d_T=1$ (up to one deletion from the tree, representing missing genes) and $d_S=3$ (up to three deletions from the plasmid, representing intruding genes). An instance of a gene cluster is accepted if it was derived from the corresponding PQ-tree with a score that is higher than 0.75 of the highest possible score attainable by the query. This search resulted in the detection of approximate instances of the query gene cluster in the plasmids of 24 genomes; These results are displayed in Figs. 5, 6, and Additional file 1: Figure S1.

Heavy metal efflux pumps are involved in the resistance of bacteria to a wide range of toxic metal ions [47] and they belong to the resistance-nodulation-cell division (RND) family. In Gram-negative bacteria, RND pumps exist in a tripartite form, comprised from an outer-membrane protein (OMP), an inner membrane protein (IMP), and a periplasmic membrane fusion protein (MFP) that connects the other two proteins. In some cases, the genes of the RND pump are flanked with two regulatory genes that encode the factors of a two-component regulatory system comprising a sensor/histidine kinase (HK) and response regulator (RR) (Fig. 5B). This regulatory system responds to the presence of a substrate, and consequently enhances the expression of the efflux pump genes.

The PQ-tree of this gene cluster (Fig. 5A) shows that the COGs encoding the IMP and MFP proteins always appear as an adjacent pair, the OMP COG is always adjacent to this IMP-MFP pair, and the HK and RR COGs appear as a pair downstream or upstream to the other COGs. COG3696, which encodes the IMP protein, is annotated as a heavy metal efflux pump protein, while the other COGs are common to all RND efflux pumps. Therefore, it is very likely that the respective gene cluster corresponds to a heavy metal RND pump. The absence of an additional periplasmic protein likely indicates that this gene cluster encodes a Czc-like efflux pump that exports divalent metals such as the cobalt, zinc and cadmium exporter in *Cupriavidus metallidurans* [47] (Fig. 5C(1)).

PQFinder discovered instances of this gene cluster in the plasmids of 12 genomes (Fig. 5C(1) and D), and it is significantly enriched in the $\beta$-proteobacteria class (hypergeometric p-value= $1.09\times 10^{-5}$, Bonferroni corrected p-value = $1.09\times 10^{-4}$). In addition, three other variants of RND pumps were found as instances of the query gene cluster (Fig. 5C(2–4)). The plasmids of three genomes contained instances that were missing the COG corresponding to the OMP gene CzcC (Fig. 5C(2)). This could be caused by a low quality sequencing or assembly of these plasmids. An alternative possible explanation is that a Czc-like efflux pump can still be functional without CzcC; a previous study showed that the deletion of CzcC resulted in the loss of cadmium and cobalt resistance, but most of the zinc resistance was retained [47].

Some instances identified by the query, found in the plasmids of six genomes, seem to encode a different heavy metal efflux pump (Figs. 5C(3), 6). This variant includes all COGs from the query, in addition to an intruding COG that encodes a periplasmic protein (CusF). This protein is a predicted copper usher that facilitates access of periplasmic copper towards the heavy metal efflux pump. Indeed, the genomic region of Cus-like efflux pumps that export monovalent metals, such as the silver and copper exporter in *Escherichia coli*, include this periplasmic protein, in contrast to the Czc-like efflux pump [47]. This variant was found in the plasmids of six bacterial genomes belonging to the class $\gamma$-proteobacteria (Fig. 5D). This gene cluster is significantly enriched in the $\gamma$-proteobacteria class (hypergeometric p-value= $2.13\times 10^{-4}$, Bonferroni corrected p-value = $2.13\times 10^{-3}$). Surprisingly, all of these strains, except for one, are annotated as human or animal pathogens. Interestingly, previous studies suggest that the host immune system exploits excess copper to poison invading pathogens [48], which can explain why these pathogens evolved copper efflux pumps.

Another variant of the pump, appearing in five genomes (Fig. 5C(4) and D), resulted from a substitution of the query IMP gene (COG3696) by a different IMP gene (COG0841) belonging to the multidrug efflux pump AcrAB-TolC. The AcrAB-TolC system, mainly studied in *Escherichia coli*, transports a diverse array of compounds with little chemical similarity [49]. AcrAB-TolC is an example of an intrinsic non-specific efflux pump, which is widespread in the chromosomes of Gram-negative bacteria, and likely evolved as a general response to environmental toxins [50]. In this case, the query gene cluster and the identified variant share all COGs, except for the COGs encoding the IMP genes. The differing COGs are responsible for substrate recognition, which naturally differs between the two pumps, as one pump exports heavy metal while the other exports multiple drugs. When considering the functional annotation of these two COGs, we see that the query metal efflux pump COG encoding the IMP gene is annotated as “Cu/Ag efflux pump CusA”, while in the multidrug efflux pump the COG encoding the IMP gene is annotated as “Multidrug efflux pump subunit AcrB”. Thus, in spite of the difference in substrate specificity, the semantic similarity measure employed by PQFinder was able to reflect their functional similarity and allowed the substitution between them, while conferring to the structure of the PQ-tree.

## PQ-tree search is NP-hard

In this section we prove Theorem 2 by describing a reduction from the Job Interval Selection problem (JISP) to PQ-Tree Search. This reduction also proves that PQ-Tree Alignment is NP-hard (Theorem 3).

### Theorem 2

PQ-Tree Search is NP-hard.

### Theorem 3

PQ-Tree Alignment is NP-hard.

Since its initial definition by Nakajima and Hakimi [51], JISP has seen several equivalent definitions [43, 52–54]. We use the following formulation for JISP *k* based on colors. Given $\gamma$ *k*-tuples of intervals on the real line, where the intervals of every *k*-tuple have a different color *i* ($1\le i \le \gamma$), select exactly one interval of each color (*k*-tuple) such that no two intervals intersect. The notation $I_j^i$ is used to denote the interval that starts at $s_{ij}$, ends at $f_{ij}$ (i.e. the interval $[s_{ij},f_{ij}]$) and has the color *i* (i.e. it is a part of the $i^{\text {th}}$ *k*-tuple).

JISP3 was shown to be NP-complete by Keil [52]. Crama et al. [54] showed that JISP3 is NP-complete even if all intervals are of length 2. We use these results to show that PQ-Tree Search is NP-hard.

**Fig. 7:**
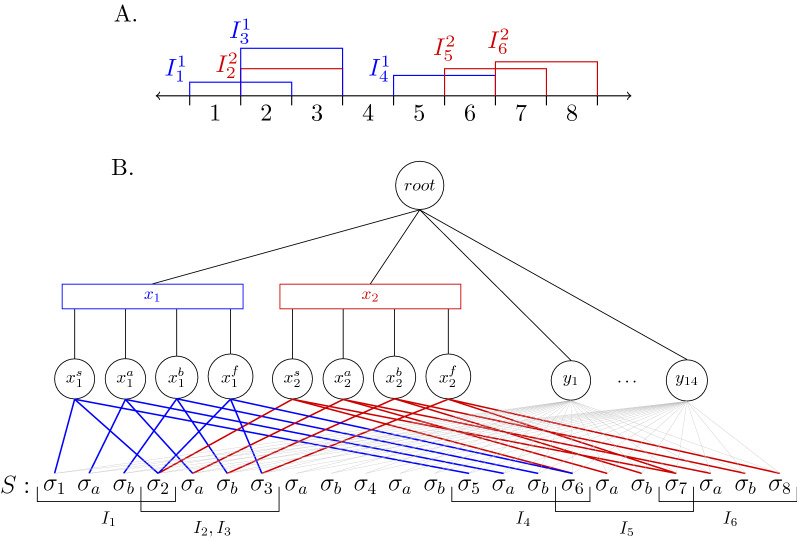
**A** The input of the reduction—a JISP3 instance *J* with intervals of length 2. **B** The output of the reduction—a PQ-Tree Search instance $(T,S,h,d_T,d_S)$

*The reduction*. Let *J* be an instance of JISP3 where all intervals have a length of two. It is easy to see that shifting all intervals by some constant does not change the problem. Hence, assume that the leftmost starting interval starts at 1. Let *L* be the rightmost ending point of an interval, so the focus can be only on the segment [1, *L*] of the real line. Now, an instance of PQ-Tree Search $(T,S,h,d_T,d_S)$ is constructed (an illustrated example is given in Fig. 7 below):

- **The PQ-tree** *T* **:** The root node, $root_T$, is a P-node with $3L-2-3\gamma$ children: $x_1, \cdots {,} x_{\gamma },y_1, \cdots {,} y_{3L-2-4\gamma }$. The children of $root_T$ are defined as follows: for every color $1 \le i \le \gamma$, create a Q-node $x_i$ with four children $x_i^s, \ x_i^a, \ x_i^b, \ x_i^f$; for every index $1\le i\le 3L-2-3\gamma$, create a leaf $y_i$.
- **The string** *S* **:** Define $S=\sigma _1 \sigma _a \sigma _b \sigma _2 \sigma _a \sigma _b \cdots \sigma _a \sigma _b \sigma _L$.
- **The substitution function** *h* **:** For every interval of the color *i*, $I_j^i=[s_{ij},f_{ij}]$, the function *h* returns *True* for the following pairs: $(x_i^s, \sigma _{s_{ij}})$, $(x_i^f, \sigma _{f_{ij}})$, $(x_i^a, \sigma _a)$ and $(x_i^b, \sigma _b)$. In addition, every leaf $y_r$ can be substituted by every character of *S*, namely for every index $1\le r\le 3L-2-3\gamma$ and for every $s \in \{a,b,1,\cdots ,L\}$ the function *h* returns *True* for the pair $(y_r, \sigma _s)$. For every other pair *h* returns *False*. For the optimization version of the problem, define a substitution scoring function $h'$, such that $h'(u,v) = 1$ if $h(u,v) = True$ and $h'(u,v) = -\infty$ if $h(u,v) = False$.
- **Number of deletions:** Define $d_T=0$ and $d_S=0$, i.e. deletions are forbidden from both tree and string.

An example of the reduction is shown in Fig. 7. The JISP3 instance *J* is a collection of two 3-tuples (one blue and one red) where each interval is of length 2 (Fig. 7A). Running the reduction algorithm on *J* yields the PQ-Tree Search instance in Fig. 7B. The pairs that can be substituted (i.e. the pairs for which *h* returns *True*) are given by the lines connecting the leaves of the PQ-tree and the characters of the string *S*. The nodes and substitutable pairs created due to the blue and red intervals in the JISP3 instance are marked in blue and red, respectively. The substitutable pairs containing a *y* node are marked in gray. Note that the colors given in Fig. 7B are not a part of the PQ-Tree Search instance, and are given for convenience.

Notice that in the reduction, the number of deletions is zero and the height of the tree is 2. Thus, these parameters cannot be used to design an FPT algorithm. In addition, notice that though the output of the reduction is referred as an instance of PQ-Tree Search, it is also an instance of PQ-Tree Alignment. Ahead the reduction is proven for PQ-Tree Search, but the proof for PQ-Tree Alignment is the same.

### *Proof*

*Correctness* Let *J* be an instance of JISP3, and let $(T,S,h,d_T,d_S)$ be the output of the reduction on this instance. We prove that there exists a collection of intervals that is a solution for *J* if and only if there exists a one-to-one mapping that is a solution to $(T,S,h,d_T,d_S)$.

*One direction*. Suppose that there exists a solution to the output instance of PQ-Tree Search of the reduction, $(T,S,h,d_T,d_S)$. This solution is a one-to-one mapping ${\mathcal {M}}{}$: for every $1\le i\le \gamma$, a set of pairs of the form $(x_i^j,\sigma _k(\ell ))$ for $j\in \{s,f,a,b\}$, and for every $1\le r\le 3L-2-3\gamma$, pairs of the form $(y_r,\sigma _k(\ell ))$ where $k\in \{1,\cdots ,L,a,b\}$ and $1\le \ell \le 3L-2$. By the definition of PQ-Tree Search, each $x_i^j$, $y_r$ and $\sigma _k(\ell )$ appear in exactly one pair. Considering the mappings of the children of a node $x_i$, they must be the following: $(x_i^s,\sigma _k(\ell ))$, $(x_i^a,\sigma _a(\ell +1))$, $(x_i^b,\sigma _b(\ell +2))$ and $(x_i^f,\sigma _{k+1}(\ell +3))$. To see this, observe that a node $x_i^a$ must be mapped to $\sigma _a$, because it is the only character by which it can be substituted under *h*. In the same way, a node $x_i^b$ must be mapped to $\sigma _b$. Because $d_T=0$, $d_S=0$ and due to the properties of a Q-node, once $x_i^s$ is mapped to the character in index $\ell$ (i.e. $(x_i^s,\sigma (\ell ))\in {\mathcal {M}}{}$), $x_i^a$ must be mapped to the character in index $\ell +1$ or in index $\ell -1$ (i.e. the adjacent character to the one to which $x_i^s$ is mapped), then $x_i^b$ must be mapped to the character in index $\ell +2$ or $\ell -2$, respectively, and $x_i^f$ to $\ell + 3$ or $\ell -3$, respectively. Since $\sigma _a$ is always the character preceding $\sigma _b$ in *S*, $x_i^b$ must be mapped to an index larger by one than the index mapped to $x_i^a$. Hence, the children of the Q-node $x_i$ are mapped from left to right.

Now, let us derive a solution for the original JISP3 instance from the solution to PQ-Tree Search. For every 3-tuple of color $1\le i\le \gamma$, where $(x_i^s,\sigma _k(\ell ))\in$ ${\mathcal {M}}{}$, choose the interval $I_k^i=[k,k+1]$ from the 3-tuple of color *i*. For example, if a part of the solution for the PQ-Tree Search instance in Fig. 7B is $\{(x_1^s,\sigma _1(1)),$ $(x_1^a,\sigma _a(2)),$ $(x_1^b,\sigma _b(3)),$ $(x_1^f,\sigma _2(4))\} \subset$ ${\mathcal {M}}{}$, then $I_1^1$ is the interval chosen for the first color (blue) in the derived solution for the JISP3 instance in Fig. 7.A. Note that $I_k$ is indeed one of the intervals of color *i*, due to the definition of *h*, $h(x_i^s,\sigma _k) = True$ and $h(x_i^f,\sigma _{k+1})=True$ if and only if there is an interval of color *i* starting at *k* and ending at $k+1$. Thanks to ${\mathcal {M}}{}$ being a one-to-one mapping, the intervals do not intersect, and for every color there is only one interval chosen.

*Second Direction.* Let us prove that if there is a solution for the original instance of JISP3 *J*, then there is a solution for $(T,S,h,d_T,d_S)$. Let ${\mathcal {I}}=\{I_{j_1}^1,...,I_{j_\gamma }^\gamma \}$ be a solution of *J* such that $I_{j_i}^i=[s_{ij_i},f_{ij_i}]$ is the interval chosen for the 3-tuple of color *i*. First, the solution for the PQ-Tree Search instance $(T,S,h,d_T,d_S)$ is constructed. For every $1\le i\le \gamma$, insert the following pairs into ${\mathcal {M}}{}$: $(x_i^s,\sigma _{s_{ij_i}}(3s_{ij_i}-2))$, $(x_i^a,\sigma _{a}(3s_{ij_i}-1))$, $(x_i^b,\sigma _{b}(3s_{ij_i}))$, and $(x_i^f,\sigma _{f_{ij_i}}(3f_{ij_i}-2))$. For example, if $I_2^2$ is the interval chosen from the second (red) 3-tuple in the solution of the JISP3 instance in Fig. 7.A, then the solution for the PQ-Tree Search instance in Fig. 7B includes the pairs $\{(x_2^s,\sigma _2(4)), (x_2^a,\sigma _a(5)), (x_2^b,\sigma _b(6)), (x_2^f,\sigma _3(7))\}$. Observe that only one pair was inserted for every leaf of *T*, and since no two intervals intersect, every index of *S* appears in only one pair in ${\mathcal {M}}{}$. Hence, a one-to-one mapping between $4\gamma$ leaves of *T* and $4\gamma$ indices of *S* was defined, and $3L-4\gamma -2$ additional pairs need to be inserted to ${\mathcal {M}}{}$ in order to construct a solution for the PQ-Tree Search instance. According to *h*, every node $y_r$ ($1\le r\le 3L-2-3\gamma$) can be mapped to every character $\sigma _k$, so arbitrarily insert the pairs $(y_r,\sigma _{k_r}(\ell _r))$ to ${\mathcal {M}}{}$, such that no index or node appear in more than one pair. (It can be done because there are $3L-4\gamma -2$ *y* nodes and after mapping the 4 children of every one of the $\gamma$ $x_i$ nodes, $3L-4\gamma -2$ characters of *S* are left without a mapping). Thus, a one-to-one mapping ${\mathcal {M}}{}$ between all the leaves of *T* and all the indices of *S* (i.e. no deletions from *S* and *T*) was defined, and it is left to prove that *S* can be derived from *T* under ${\mathcal {M}}{}$.

The children of a Q-node $x_i$ from left to right are: $x_i^s,x_i^a,x_i^b,x_i^f$, and so, because $d_T=0$ and $d_S=0$ (no deletions from both tree and string), they have to be mapped to consecutive indices of *S*; this is indeed the case according to our definition of ${\mathcal {M}}{}$. The mapping of every $y_r$ is obviously also legal. Finally, $root_T$ is a P-node, so its children can be arranged in any order, and they are. This completes the proof of correctness of the reduction. $\square$

This concludes the proof of Theorem 2.

## Correctness of our algorithms

In this section we prove the correctness of the PQ-Tree Search algorithm ("Correctness of the main algorithm" section) and the P-mapping algorithm ("Correctness of the P-node mapping algorithm" section). First, some definitions that are used in the proofs are given.

**Addition and removal of a derivation.** Given a partial derivation $\mu$, which derives an internal node *x*, let us define the removal and addition of another derivation $\eta$: $\mathsf {remove}(\mu ,\eta )$ and $\mathsf {add}(\mu ,\eta )$. To this end, we say that $\eta$ is *the derivation of* $x'$ *under* $\mu$ if $x'=\eta .v\in \mathsf {children}(\mu .v)$ and $\eta .o \subseteq \mu .o$, i.e. the one-to-one mapping that yields $\eta$ is a subset of the one-to-one mapping that yields $\mu$.

### Operation 1

The operation $\mathsf {remove}(\mu ,\eta )$ is defined only if $\eta$ is the derivation of $\eta .v$ under $\mu$ and if either $\eta .e = \mu .e$ or $\eta .s=\mu .s$ is true. The operation returns a new partial derivation $\mu '$ of $\mu .v$ that ignores the subtree $T(\eta .v)$. If $\eta .e = \mu .e$, then $\mu '$ derives the string $S[\mu .s:\eta .s-1]$, and if $\eta .s=\mu .s$, then $\mu '$ derives the string $S[\eta .e+1:\mu .e]$. In any case the number of deletions from the tree is $\mu '.del_T= \mu .del_T- \eta .del_T$ and from the string it is $\mu '.del_S= \mu .del_S- \eta .del_S$. Furthermore, $\mu .o \setminus \eta .o$ is the one-to-one mapping that yields $\mu '$.

### Operation 2

The operation $\mathsf {add}(\mu ,\eta )$ is defined only if either $\eta .s = \mu .e+1$ or $\eta .e=\mu .s-1$ is true and if $\eta .v\in \mathsf {children}(x)$ and it is ignored under $\mu$. The operation returns a new partial derivation $\mu '$ of $\mu .v$. The derivation of $\eta .v$ under $\mu '$ is $\eta$, and the mapping or deletion of every other leaf or character in the string is defined the same as it was in $\mu$. Consequentially, if $\eta .s = \mu .e+1$, then $\mu '$ derives the string $S[\mu .s:\eta .e]$, and if $\eta .e=\mu .s-1$, then $\mu '$ derives the string $S[\eta .s:\mu .e]$. In any case, $\mu '.del_T= \mu .del_T+ \eta .del_T$, $\mu '.del_S= \mu .del_S+ \eta .del_S$ and the one-to-one mapping that yields $\mu '$ is $\mu .o \cup \eta .o$.

**Addition and removal of a deleted character.** Given a partial derivation $\mu$, which derives a string *S*, and an index *i* of *S* let us define the removal and addition of a deleted character: $\mathsf {removeDel}(\mu , i)$ and $\mathsf {addDel}(\mu ,i)$.

### Operation 3

The operation $\mathsf {removeDel}(\mu , i)$ is defined only if $i=\mu .e$ or $i=\mu .s$, and if *S*[*i*] is deleted under $\mu$. The operation returns a partial derivation $\mu '$ with $\mu .del_S-1$ deletions from the string. If $i=\mu .e$, then $\mu '$ derives the string $S[\mu .s,\mu .e-1]$, and if $i=\mu .s$, then $\mu '$ derives the string $S[\mu .s+1,\mu .e]$. The one-to-one mapping that yields $\mu '$ is $\mu .o \setminus \{(\varepsilon , S[i](i))\}$.

### Operation 4

The operation $\mathsf {addDel}(\mu ,i)$ is defined only if $i=\mu .e+1$ or $i=\mu .s-1$. The operation returns a partial derivation $\mu '$ with $\mu .del_S+1$ deletions from the string. If $i=\mu .e+1$, then $\mu '$ derives the string $S[\mu .s,\mu .e+1]$, and if $i=\mu .s-1$, then $\mu '$ derives the string $S[\mu .s-1,\mu .e]$. The one-to-one mapping that yields $\mu '$ is $\mu .o \cup \{(\varepsilon , S[i](i))\}$.

### Correctness of the main algorithm

In this section we prove the correctness of the PQ-Tree Search algorithm presented in "The main algorithm" section by proving Lemma 4. In this proof, the correctness of the Q-mapping algorithm (described in Sect. 1 of Additional file 1) and of the P-mapping algorithm (described in "P-node mapping: the algorithm" section) is assumed. In addition, the set of all derivations to $S[i,E(x,i,k_T,k_S)]$ rooted in *x* that have exactly $k_T$ deletions from the tree and exactly $k_S$ deletions from the string is denoted by ${\mathcal {D}}_M(x,i,k_T,k_S){}$. Similarly to the notation in Definition 2, the ${\mathcal {D}}_M(x,i,k_T,k_S){}$ notation is used to represent the set of derivations whose score might be in ${\mathcal {A}}[x,i,k_T,k_S]$.

#### Lemma 4

At the end of the algorithm every entry ${\mathcal {A}}[x,i,k_T,k_S]$ of the DP table ${\mathcal {A}}{}$ holds the highest score of a derivation of $S[i,E(x,i,k_T,k_S)]$ rooted in *x* that has $k_S$ deletions from the string and $k_T$ deletions from the tree, i.e. ${\mathcal {A}}[x,i,k_T,k_S] = \max _{\mu \in {\mathcal {D}}_M(x,i,k_T,k_S){}}\mu .score$

#### *Proof*

We prove Lemma 4 by induction on the entries of ${\mathcal {A}}{}$ in the order described in the algorithm. Namely, for two entries ${\mathcal {A}}[x_1,i_1, k_{T_1},k_{S_1}]$ and ${\mathcal {A}}[x_2,i_2,k_{T_2},k_{S_2}]$, ${\mathcal {A}}[x_1,i_1, k_{T_1},k_{S_1}] < {\mathcal {A}}[x_2,i_2,k_{T_2},k_{S_2}]$ if and only if $x_1$ appears before $x_2$ in the postorder of *T* or both $x_1=x_2$ and $i_1<i_2$. If $x_1=x_2$ and $i_1=i_2$, then the order between the entries is chosen arbitrarily.

*Base Case.* The base case of the algorithm is the initialization of the DP table, where the entries ${\mathcal {A}}[x,i,k_T,k_S]$ for $x\in \mathsf {leaves}(root)$ and $k_T \in \{0,1\}$ are computed. When $k_T=0$, there are no deletions from the tree. So, *x* must be mapped to some character $S[\ell ]$ ($i\le \ell \le E(x,i,0,k_S)$). In this version of the algorithm the deletion of a character does not change the score of the derivation, so the maximal score of a derivation in ${\mathcal {D}}_M(x,i,0,k_S)$ is the maximum score of a mapping of *x* to some character $S[\ell ]$ ($i\le \ell \le E(x,i,0,k_S)$), which is the initialization value of the entry ${\mathcal {A}}[x,i,0,k_S]$. When $k_T=1$, there is one deletion from the tree. The derived subtree *T*(*x*) has one leaf, *x*, and so it must be the deleted leaf. All characters in the derived string, $S[i:E(x,i,1,k_S)]$, must also be deleted. Deletions do not add to the score of the derivation, and so all the derivations in ${\mathcal {D}}_M(x,i,1,k_S)$ have a score of 0, which is the initialization value of ${\mathcal {A}}[x,i,1,k_S]$.

*Induction Assumption.* Assume that every entry ${\mathcal {A}}[x',i',k'_T,k'_S]$ such that ${\mathcal {A}}[x',i',k'_T,k'_S]$ $< {\mathcal {A}}[x,i,k_T,k_S]$ holds the best score of a derivation from the set ${\mathcal {D}}_M(x',i',k'_T,k'_S)$. Namely, ${\mathcal {A}}[x',i',k'_T,k'_S] = \max _{\mu \in {\mathcal {D}}_M(x',i',k'_T,k'_S)}{\mu .score} = OPT(x',i',k'_T,k'_S)$.

*Induction Step.* For every internal node *x* and possible start index *i*, the algorithm fills the DP table entry ${\mathcal {A}}[x,i,k_T,k_S]$ according to the values returned from the Q-mapping and P-mapping algorithms based on the type of *x*. The correctness of the P-mapping algorithm is proven in Section Correctness of the P-node mapping algorithm, and the correctness of the Q-mapping algorithm is proven in Section 1.2 of Additional file 1. Hence, it is only necessary to prove that the input the algorithms expect to receive is sent correctly from the main algorithm.

Both the Q-mapping and P-mapping algorithms expect to receive the internal node which should be the root of all the output derivations, a substring $S'$ of *S*, the deletion bounds $d_T$ and $d_S$, and a collection of the best scoring derivations of every child of *x* to every substring of $S'$ with up to $d_T$ and $d_S$ deletions from the tree and string, respectively. By definition an entry in ${\mathcal {A}}[x,i,\cdot ,\cdot ]$ concerns the derivations of *x* with a start point *i*. The end point of the longest derivation of those derivations is $E(x,i,0,d_S)$. Hence, the internal node sent to the Q-mapping or P-mapping algorithm is *x* and the substring $S'$ equals $S[i,E(x,i,0,d_S)]$. The deletion bounds $d_T$ and $d_S$ are given as input to the main algorithm. Lastly, the best derivations of the children of *x* are stored in ${\mathcal {A}}{}$. Because a node $x' \in \mathsf {children}(x)$ appears before *x* in the postorder of *T*, then for every $i', k'_T, k'_S$, it holds that ${\mathcal {A}}[x',i',k'_T,k'_S] < {\mathcal {A}}[x,i,k_T,k_S]$, and from the induction assumption ${\mathcal {A}}[x',i',k'_T,k'_S]= OPT(x',i',k'_T,k'_S)$. So, indeed the expected input to the Q-mapping and P-mapping algorithms is correct. This completes the proof. $\square$

### Correctness of the P-node mapping algorithm

In this section we prove the correctness of the P-mapping algorithm presented in "P-node mapping: the algorithm" section by proving Lemma 5.

#### Lemma 5

At the end of the algorithm every entry of the DP table, ${\mathcal {P}}[C,k_T,k_S]$, holds the best score for a partial derivation of $x^{(C )}$ to a prefix of $S'$ with $k_T$ deletions from the tree and $k_S$ deletions from the string, i.e. ${\mathcal {P}}[C,k_T,k_S] = \max _{\mu \in {\mathcal {D}}(x^{(C )},k_T,k_S){}}\mu .score$

#### *Proof*

We prove Lemma 5 by induction on the entries of ${\mathcal {P}}{}$ in the order described in the algorithm. Namely, for two entries ${\mathcal {P}}[C_1,k_{T_1},k_{S_1}]$ and ${\mathcal {P}}[C_2,k_{T_2},k_{S_2}]$, ${\mathcal {P}}[C_1,k_{T_1},k_{S_1}] < {\mathcal {P}}[C_2,k_{T_2},k_{S_2}]$ if and only if

- $$|C_1| < |C_2|$$, or
- $$|C_1| = |C_2|$$ and $$k_{S_1} < k_{S_2}$$, or
- $$|C_1| = |C_2|$$ and $$k_{S_1} = k_{S_2}$$ and $$k_{T_1} < k_{T_2}$$

If $C_1 \ne C_2$, $|C_1| = |C_2|$, $k_{S_1} = k_{S_2}$ and $k_{T_1} = k_{T_2}$ are all satisfied, then the order between the entries is chosen arbitrarily.

*Base Cases.* There are two types of base cases, as described in the initialization of the DP table.

1. $L(x^{(C )},k_T,k_S)=0$ and $k_S=0$: Let $\mu$ be a derivation of $x^{(C )}$ with $k_T$ and $k_S$ deletions. By definition, $\mu$ derives an empty string, i.e. there are no characters to map to the leaves of the subtrees rooted in the nodes in *C*. Hence, every child of *x* that is considered (the nodes in *C*) must be deleted under $\mu$. All the nodes in *C* can be deleted if the sum of their spans is equal to the allowed number of deletions in $\mu$ (that is, $k_T$). From the definition of $L(x^{(C )},k_T,k_S)=0$ and the fact that $k_S=0$, we obtain that indeed $k_T = \sum _{c\in C}\mathsf {span}(c)$. Every child node of *x* that is kept under $\mu$ adds to the score of the derivation of *x*, but there are none in this case. In addition, every deletion from the subtree *T*(*x*) adds nothing to the score (in the penalty-free version of the algorithm). Hence, the score of $\mu$ must equal 0.
2. $C=\emptyset$ and $k_T=0$: In this case all of the children of *x* are ignored, so there are no leaves to map. Hence, every character of the derived string should be deleted. Note that the derived string is $S'[1:E_{I}(x^{(C )},k_T,k_S)]$, and its length is $L(x^{(C )},k_T,k_S) = \sum _{c\in C}\mathsf {span}(c) - k_T + k_S = \sum _{c\in \emptyset }\mathsf {span}(c) - 0 + k_S = k_S$. So, the number of deletions from the string in this case is exactly the number needed to delete all the characters in the derived string.

*Induction Assumption.* Assume that every table entry ${\mathcal {P}}[C',k'_T,k'_S]$ such that ${\mathcal {P}}[C',k'_T,k'_S]$ $< {\mathcal {P}}[C,k_T,k_S]$ holds the best score of a derivation in ${\mathcal {D}}(C',k'_T,k'_S)$. Namely, ${\mathcal {P}}[C',k'_T,k'_S]$ $= \max _{\mu \in {\mathcal {D}}(C',k'_T,k'_S)}{\mu .score} = OPT(C',k'_T,k'_S)$.

*Induction Step.* Towards the proof of the step, we prove the following Eq. 5:

5 $$\begin{aligned} \begin{aligned} OPT(C,&k_T,k_S) = \max (OPT(C,k_T,k_S-1),\\&\displaystyle \max _{\mu \in {\mathcal {D}}_\le (C,k_T,k_S){}} {OPT(C\setminus \{\mu .v\},k_T-\mu .del_T,k_S-\mu .del_S) + \mu .score}) \end{aligned} \end{aligned}$$

$$\le$$:
Let $\mu ^* \in {\mathcal {D}}(x^{(C )},k_T,k_S)$ be a derivation such that $\mu ^*.score = OPT(C,k_T,k_S)$, and let $e_c=E_{I}(x^{(C )},k_T,k_S)$. By definition, $\mu ^*$ is a derivation of $x^{(C )}$ to the string $S'[1:e_c]$. In a derivation every character of the derived string is either deleted or it is a part of a substring derived from one of the children of *x*. So, either $S'[e_c]$ is deleted under $\mu ^*$, or it is mapped under some derivation of a child of $x^{(C )}$, to a substring $S'[i:e_c]$ (for an index $0<i\le e_c$). First, if the former is true, then by removing the deletion of $S'[e_c]$ from $\mu ^*$ ($\mathsf {removeDel}(\mu ^*, E_{I}(x^{(C )}, k_T, k_S))$) a derivation $\mu ' \in {\mathcal {D}}(x^{(C )},k_T,k_S-1)$ is obtained. The derivation $\mu '$ derives the string $S'[1:E_{I}(x^{(C )},k_T,k_S-1)] = S'[1:e_c - 1]$. So, the following Eq. 6 is true.
  6 $$\begin{aligned} \begin{aligned} \mu ^*.score&= \mu '.score\\&\le OPT(C,k_T,k_S-1)\\&\le \max (OPT(C,k_T,k_S-1),\\&\displaystyle \max _{\mu \in {\mathcal {D}}_\le (C,k_T,k_S){}} {OPT(C\setminus \{\mu .v\},k_T-\mu .del_T,k_S-\mu .del_S) + \mu .score}) \end{aligned} \end{aligned}$$
  Note that even if there is a penalty cost for deletions, the cost for the deletion of $S'[e_c]$ (i.e. $-\Delta (S'[e_c])$) is constant in this setting. So, for two derivations $\eta , \eta ' \in {\mathcal {D}}(x^{(C )},k_T,k_S-1)$ if $\eta .score \le \eta '.score$ then $\eta .score -\Delta (S'[e_c]) \le \eta '.score -\Delta (S'[e_c])$. Hence, the conclusion from Eq. 6 is still true. Second, if the latter is true, then there is a node $y \in C$ for which there is a derivation $\mu _y \in {\mathcal {D}}{}$ such that $\mu _y.e = e_c$ and $\mu .y$ is the derivation of *y* under $\mu ^*$. For $\mu ^*$ to be a legal derivation, $\mu _y$ must be in ${\mathcal {D}}_\le (C,k_T,k_S){}$. Hence, $\mu _y.score \le \max _{\mu \in {\mathcal {D}}_\le (C,k_T,k_S){}}{\mu .score}$. Furthermore, by removing $\mu _y$ from $\mu ^*$, $\mathsf {remove}(\mu ^*,\mu .y)$, the obtained partial derivation, $\mu '$, is of $x^{(C \setminus \{y\})}$ to $S'[1:\mu _y.s-1]$ with $k_T-\mu _y.del_T$ deletions from the tree and $k_S-\mu _y.del_S$ from the string. Thus, $\mu ' \in {\mathcal {D}}(x^{(C \setminus \{y\})},k_T-\mu _y.del_T, k_S-\mu _y.del_S)$, and so $\mu '.score \le OPT(C\setminus \{y\}, k_T-\mu _y.del_T, k_S-\mu _y.del_S)$. Note that indeed $\mu _y.s = 1 + E_{I}(x^{(C \setminus \{y\})},k_T-\mu _y.del_T, k_S-\mu _y.del_S)$, as can be seen in the following Eq. 7.
  7 $$\begin{aligned} \begin{aligned} \mu _y.s&= e_c - L(y,\mu _y.del_T, \mu _y.del_S) + 1 \\&= \sum _{c\in C}{\mathsf {span}(c)} + k_S - k_T - (\mathsf {span}(y) + \mu _y.del_S- \mu _y.del_T) + 1 \\&= \sum _{c\in C\setminus \{y\}}{\mathsf {span}(c)} + k_S-\mu _y.del_S- (k_T-\mu _y.del_T) + 1 \\&= E_{I}(x^{(C \setminus \{y\})},k_T-\mu _y.del_T, k_S-\mu _y.del_S) + 1 \end{aligned} \end{aligned}$$
  By combining our conclusions about $\mu _y$ and $\mu '$ together, we obtain the following Eq. 8.
  8 $$\begin{aligned} \begin{aligned} \mu ^*.score&= \mu '.score + \mu _y.score \\&\le OPT(C\setminus \{y\},k_T-\mu _y.del_T, k_S-\mu _y.del_S) + \max _{\mu \in {\mathcal {D}}_\le (C,k_T,k_S){}}{\mu .score} \\&\le \displaystyle \max _{\mu \in {\mathcal {D}}_\le (C,k_T,k_S){}} {OPT(C\setminus \{\mu .v\},k_T-\mu .del_T,k_S-\mu .del_S) + \mu .score} \\&\le \max (OPT(C, k_T, k_S-1), \\&\displaystyle \max _{\mu \in {\mathcal {D}}_\le (C,k_T,k_S){}} {OPT(C\setminus \{\mu .v\},k_T-\mu .del_T,k_S-\mu .del_S) + \mu .score}) \end{aligned} \end{aligned}$$
$$\ge$$:
Let $\mu ^*$ be a derivation such that Eq. 9 holds, and let $e_c=E_{I}(x^{(C )},k_T,k_S)$.
  9 $$\begin{aligned} \begin{aligned} \mu ^*.score =&\max (OPT(C, k_T, k_S-1), \\&\max _{\mu \in {\mathcal {D}}_\le (C,k_T,k_S){}} OPT(C\setminus \{\mu .v\},k_T-\mu .del_T,k_S-\mu .del_S) + \mu .score) \end{aligned} \end{aligned}$$
  So, either $\mu ^*.score = OPT(C, k_T, k_S-1)$, or $\mu ^*.score = \displaystyle \max _{\mu \in {\mathcal {D}}_\le (C,k_T,k_S){}}$ $OPT(C\setminus \{\mu .v\},$ $k_T-\mu .del_T,k_S-\mu .del_S) + \mu .score$. First, if the former is true, let $\eta \in {\mathcal {D}}(x^{(C )},k_T,k_S-1)$ be a derivation with $\eta .score = OPT(C, k_T, k_S-1)$. By definition, $\eta$ derives the substring $S'[1:E_{I}(x^{(C )},k_T,k_S-1)]$. Adding to $\eta$ the deletion of $S'[e_c]$, $\mathsf {addDel}(\eta ,e_c)$, results in a derivation $\eta '$ of $x^{(C )}$ to the string $S'[1:e_c]$ with $k_T$ deletions from the tree and $k_S$ deletions from the string. The string $S'[1:e_c]$ is equal to the concatenation of $S'[1:E_{I}(x^{(C )},k_T,k_S-1)]$ and $S'[e_c]$. So, $\eta ' \in {\mathcal {D}}(x^{(C )},k_T,k_S){}$, and thus $\eta '.score \le OPT(C, k_T, k_S)$. The derivation $\eta '$ was constructed such that $\mu ^*.score = \eta '.score$, so $\mu ^*.score \le OPT(C, k_T, k_S)$. Second, if the latter is true, then let $\eta ^* = {{\,\mathrm{arg\max }\,}}_{\mu \in {\mathcal {D}}_\le (C,k_T,k_S){}}OPT(C\setminus \{\mu .v\}, k_T - \mu .del_T, k_S - \mu .del_S) + \mu .score$. Adding $\eta ^*$ to a partial derivation $\eta \in {\mathcal {D}}(x^{(C \setminus \{\eta ^*.v\})},k_T - \eta ^*.del_T, k_S - \eta ^*.del_S)$, $\mathsf {add}(\eta ,\eta ^*)$, results in a partial derivation, $\eta '$, with $k_T-\eta ^*.del_T+ \eta ^*.del_T= k_T$ deletions from the tree and $k_S-\eta ^*.del_S+ \eta ^*.del_S= k_S$ deletions from the string, that takes into account the children of *x* that are in $C\setminus \{\eta ^*.v\} \cup \{\eta ^*.v\} = C$. It is a legal partial derivation since $\eta ^*$ derives the node $\eta ^*.v$ that is not in $C\setminus \{\eta ^*.v\}$ to a string that does not intersect with the string derived by $\eta$. The string that is derived by $\eta$ is $S'[\eta .s:\eta .e]$ and it does not intersect with the string derived by $\eta ^*$ ($S'[\eta ^*.s:\eta ^*.e]$). That is because $\eta .e + 1 = \eta ^*.s$, as can be seen similarly to Eq. 7. So, $\eta ' \in {\mathcal {D}}(x^{(C )},k_T,k_S)$, and thus $\eta '.score \le OPT(C, k_T, k_S)$. The partial derivation $\eta '$ was constructed such that $\mu ^*.score = \eta '.score$, so $\mu ^*.score \le OPT(C, k_T, k_S)$.

From the induction assumption, ${\mathcal {P}}[C, k_T, k_S-1] = OPT(C, k_T, k_S-1)$ and for every $\mu \in {\mathcal {D}}_\le (C,k_T,k_S)$, ${\mathcal {P}}[C\setminus \{\mu .v\}, k_T - \mu .del_T, k_S - \mu .del_S] = OPT(C\setminus \{\mu .v\}, k_T - \mu .del_T, k_S - \mu .del_S)$. Thus, from Eq. 5, it follows that ${\mathcal {P}}[C, k_T, k_S] = OPT(C, k_T, k_S)$. This completes the proof. $\square$

## Time and Space Complexity of the PQ-Tree Search Algorithm

In this section the complexity of the main algorithm for PQ-Tree Search as well as the complexity of the P-mapping algorithm are proven.

### Time and Space Complexity of the Main Algorithm

Here we prove Lemma 1.

#### *Proof*

The number of leaves in the PQ-tree *T* is *m*, hence there are *O*(*m*) nodes in the tree, i.e the size of the first dimension of the DP table, ${\mathcal {A}}{}$, is *O*(*m*). In the algorithm description ("P-node mapping" section) a bound for the possible start indices of substrings derived from nodes in *T* is given (for a node *x*, the start index *i* runs between 1 and $n-(\mathsf {span}(x)-d_T)+1$). The node with the largest span in *T* is the root which has a span of *m*. The root is mapped to the longest substring when there are $d_S$ deletions from the string. Hence, the size of the second dimension of ${\mathcal {A}}{}$ is $\Omega (n-(m+d_S)+1) = \Omega (n)$ (given that $d_S<< n$). The nodes with the smallest spans are the leaves, which have a span of 1, hence the size of the second dimension of ${\mathcal {A}}{}$ is *O*(*n*). The third and fourth dimensions of ${\mathcal {A}}{}$ are of size $d_T+1$ and $d_S+1$, respectively. In total, the DP table ${\mathcal {A}}{}$ is of size $O(d_T d_S m n)$.

In the initialization step $O(d_S m n)$ entries of ${\mathcal {A}}{}$ are computed in $O(d_S)$ time each. This holds because there are *m* leaves and *O*(*n*) start indices for every string of length $k_S\le d_S$, and it takes $O(d_S)$ time to compute the max function. There are also $O((d_T-1) d_S m n)$ entries of ${\mathcal {A}}{}$ that are computed in *O*(1) time each. These are the entries initialized with the 0 and $-\infty$ values. This results in a $O((d_T + d_S) d_S m n)$ time initialization step which can be reduced to $O(d_T d_S m n)$ by using the replacement initialization rule mentioned in " P-node mapping" section, though they are both negligible. The P-mapping algorithm is called for every P-node in *T* and every possible start index *i*, i.e. the P-mapping algorithm is called $O(n m_p)$ times. Similarly, the Q-mapping algorithm is called $O(n m_q)$ times. Thus, it takes $O(n\ (m_p \cdot \text {Time(P-mapping)} + m_q \cdot \text {Time(Q-mapping)}))$ time to fill the DP table. In the final stage of the algorithm the maximum over the entries corresponding to every combination of deletion numbers and start index ($0\le k_T\le d_T$, $0\le k_S \le d_S, 1\le i\le n-(\mathsf {span}(x)-d_T)+1$) is computed. So, it takes $O(d_T d_S n)$ time to find a derivation with maximum score. Tracing back through the DP table to find the actual mapping does not increase the time complexity.

From Lemma 2, the P-mapping algorithm takes $O(\gamma 2^{\gamma } {d_T}^2 {d_S}^2)$ time and $O(d_T d_S 2^\gamma )$ space, and from Lemma 3, the Q-mapping algorithm takes $O(\gamma {d_T}^2 {d_S}^2)$ time and $O(d_T d_S \gamma )$ space. Thus, in total, our algorithm runs in $O(n (m_p \cdot \gamma 2^{\gamma } {d_T}^2 {d_S}^2 + m_q \cdot \gamma {d_T}^2 {d_S}^2)) = O(n \gamma {d_T}^2 {d_S}^2 (m_p \cdot 2^{\gamma } + m_q))$ time. Adding to the space required for the main DP table the space required for the P-mapping algorithm (the space needed for the Q-mapping algorithm is insignificant with respect to the P-mapping algorithm) results in a total space complexity of $O(d_T d_S m n) + O(d_T d_S 2^\gamma ) = O(d_T d_S (m n + 2^\gamma ))$. This completes the proof. $\square$

### Time and Space Complexity of the P-Node Mapping Algorithm

Here we prove Lemma 2.

#### *Proof*

The most space consuming part of the algorithm is the 3-dimensional DP table. The first dimension, *C*, can be any subset of the set $\mathsf {children}(x)$, and therefore it is of size $2^{|\mathsf {children}(x)|} = 2^\gamma$. The size of the second and third dimensions (i.e. $k_T$ and $k_S$) are $d_T+1$ and $d_S+1$, respectively. Hence, the space of the DP algorithm is $O(d_T d_S 2^\gamma )$.

The algorithm has three parts: initialization, filling the DP table, and returning the derivations in the required order. The most time consuming calculation required in the initialization is the calculation of $L(x^{(C )},k_T,k_S)$. It requires summing the spans of all nodes in *C*. This calculation will also be required in the second part of the algorithm. To avoid the repetitive calculations, it is performed once for every $(C,k_T,k_S)$ tuple and the results are saved. This requires $O(d_T d_S 2^{|\mathsf {children}(x)|}) = O(d_t d_S 2^\gamma )$ space (for this is the number of such tuples). Each value is calculated in $O(|\mathsf {children}(x)|) = O(\gamma )$ time. Hence, the calculation of all the $L(x^{(C )},k_T,k_S)$ values (and thus all the $E_{I}(x^{(C )},k_T,k_S)$ values) takes $O(d_T d_S \gamma 2^\gamma )$ time and $O(d_T d_S 2^\gamma )$ space. The second part of the algorithm is done by calculating the value of every entry in the $O(d_T d_S 2^\gamma )$ entries of ${\mathcal {P}}{}$, using the recursion rule in Eq. 2. The first line among the rule takes *O*(1) time, since it involves looking in another entry of ${\mathcal {P}}{}$ and basic computations. The second line of the rule involves going over all derivations $\mu \in {\mathcal {D}}_\le (C,k_T,k_S){}$. Namely, going over all derivations with a specific end point, which derives a node in *C* and has no more than a specific number of deletions from the tree and string (i.e. $\mu .e=E_{I}(C,k_T,k_S)$, $\mu .v \in C$, $\mu .del_T\le k_T$ and $\mu .del_S\le k_S$). The number of deletions from the tree and string are bounded by $d_T$ and $d_S$, respectively, and the number of nodes in *C* is bounded by the number of children of *x*, $\gamma$. Hence, the time to calculate one entry of ${\mathcal {P}}{}$ is $O(d_T d_S \gamma )$. In total, the second part of the algorithm takes $O({d_T}^2 {d_S}^2 \gamma 2^\gamma )$ time. Finally, to construct the returned set of derivations, the algorithm goes over every deletion combination $k_T,k_S$ once, i.e. it takes $O(d_T d_S)$ time. In total, the algorithm takes $O({d_T}^2 {d_S}^2 \gamma 2^\gamma ) + O(d_T d_S \gamma 2^\gamma ) + O(d_T d_S) = O({d_T}^2 {d_S}^2 \gamma 2^\gamma )$ time. $\square$

## Conclusions

In this paper, we defined two new problems in comparative genomics, denoted PQ-Tree Search and PQ-Tree Alignment, where the second is a sub-problem of the first. Both problems take as input a PQ-tree *T* representing the known gene orders of a gene cluster of interest, a gene-to-gene substitution scoring function *h*, integer arguments $d_T$ and $d_S$, and a sequence of genes *S*. The objective in PQ-Tree Search is to identify an approximate instance $S'$ of the gene cluster, such that $S'$ is a substring of *S*. The objective of PQ-Tree Alignment is to determine whether $S'$ is an approximate instance of the gene cluster; An approximate instance could vary from the known gene orders by genome rearrangements that are constrained by *T*, by gene substitutions that are governed by *h*, and by gene deletions and insertions that are bounded from above by $d_T$ and $d_S$, respectively.

We proved that the PQ-Tree Search and the PQ-Tree Alignment problems are NP-hard and proposed a parameterized algorithm that solves PQ-Tree Search in $O^*(2^{\gamma })$ time by solving PQ-Tree Alignment for every substring of *S*. The parameter $\gamma$ is the maximum degree of a node in *T* and $O^*$ is used to hide factors polynomial in the input size.

The proposed algorithm was implemented as a publicly available tool and harnessed to search for tree-guided rearrangements of chromosomal gene clusters in plasmids. We identified 29 chromosomal gene clusters that are rearranged in plasmids, where the rearrangements are guided by the corresponding PQ-tree. A tree-guided rearrangement event of one of these gene clusters, coding for a heavy metal efflux pump, was detected in a bacterial strain that was isolated from an environment polluted with several heavy metals. Thus, a future extension of this study could explore whether similar gene cluster rearrangement events are correlated with environmental stress or other bacterial adaptations.

The said gene cluster was further analysed to characterize its approximate instances in plasmids. An interesting variant of the analysed gene cluster, found among its approximate instances, corresponds to a copper efflux pump. It was found mainly in pathogenic bacteria, and likely constitutes a bacterial defense mechanism against the host immune response. These results exemplify how our proposed tool PQFinder can be harnessed to find meaningful variations of known biological systems that are conserved as gene clusters, and to explore their function and evolution.

Another interesting approach to perform a comparative analysis of gene clusters in chromosomes versus plasmids could theoretically be based on the alignment of PQ-trees that represent the respective gene clusters. However, this will require de-novo discovery of gene clusters in both chromosomes and plasmids—a task that is more challenging in plasmids than in chromosomes for the following two reasons. First, as it is more difficult to assemble plasmids than to assemble chromosomes, some of the plasmids may not be accurately reconstructed [11]. Second, the plasmid gene pool is more diverse and less conserved than the gene pool of chromosomes [55]. This motivated us to identify gene clusters in chromosomes and then to search for approximate tree-guided rearrangements of these gene clusters in plasmids.

One of the downsides to using PQ-trees to represent gene clusters is that very rare gene orders taken into account in the tree construction could greatly increase the number of allowed rearrangements and thus substantially lower the specificity of the PQ-tree. Thus, a natural continuation of our research would be to increase the specificity of the model by considering a stochastic variation of PQ-Tree Search and PQ-Tree Alignment. Namely, defining a PQ-tree in which the internal nodes hold the probability of each rearrangement, and adjusting the algorithms for PQ-Tree Search and PQ-Tree Alignment accordingly. In addition, future extensions of this work could also aim to increase the sensitivity of the model by incorporating gene orientation, and by taking into account gene duplications and gene-fusion events, which are typical events in gene cluster evolution.

## Supplementary Information

**Additional file 1.** Supplementary material of the paper, including additional descriptions, proofs and figures.

**Additional file 2.** A list of chromosomes and plasmids analysed in the main text.

## Data Availability

The code for the PQFinder tool as well as all the data needed to reconstruct the results in this paper are publicly available on GitHub [2]. Earlier versions of the paper can be found in [1, 56].

## Abbreviations

NP-hard: Non-deterministic Polynomial-time Hard
FPT: Fixed Parameter Tractable
JISP: Job Interval Selection Problem

## Footnotes

1 The notation O* is used to hide factors polynomial in the input size.
