# Supplementary material for: Approximate search for known gene clusters in new genomes using PQ-trees
Source: Algorithms Mol Biol. 2021 Jul 9;16:16. doi: 10.1186/s13015-021-00190-9 (PMC8272295; doi:10.1186/s13015-021-00190-9)
Supplement: Supplementary file 1 — Additional file 1. Supplementary material of the paper, including additional descriptions, proofs and figures. [file 13015_2021_190_MOESM1_ESM.pdf]

A list of chromosomes and plasmids analysed in the main text.

	Genome Name	Chromosome Accession IDs	Plasmid Accession IDs
1	Acaryochloris marina MBIC11017 uid58167	NC_009925	NC_009926, NC_009927, NC_009928, NC_009929, NC_009930, NC_009931, NC_009932, NC_009933
2	Acetobacter pasteurianus IFO 3283 01 uid59279	NC_013209	NC_013210, NC_013211, NC_013212
3	Acetohalobium arabaticum DSM 5501 uid51423	NC_014378	-
4	Acholeplasma laidlawii PG 8A uid58901	NC_010163	-
5	Achromobacter xylosoxidans A8 uid59899	NC_014640	NC_014641, NC_014642
6	Acidaminococcus fermentans DSM 20731 uid43471	NC_013740	-
7	Acidaminococcus intestini RyC MR95 uid74445	NC_016077	-
8	Acidianus hospitalis W1 uid66875	NC_015518	-
9	Acidilobus saccharovorans 345 15 uid51395	NC_014374	-
10	Acidimicrobium ferrooxidans DSM 10331 uid59215	NC_013124	-
11	Acidiphilium cryptum JF 5 uid58447	NC_009484	NC_009467, NC_009468, NC_009469, NC_009470, NC_009471, NC_009472
12	Acidiphilium multivorum AIU301 uid63345	NC_015186	NC_015178, NC_015179, NC_015180, NC_015181, NC_015187, NC_015188
13	Acidithiobacillus caldus SM 1 uid70791	NC_015850	NC_015851, NC_015852, NC_015853, NC_015854
14	Acidithiobacillus ferrivorans SS3 uid67387	NC_015942	-
15	Acidithiobacillus ferrooxidans ATCC 23270 uid57649	NC_011761	-
16	Acidithiobacillus ferrooxidans ATCC 53993 uid58613	NC_011206	-
17	Acidobacterium MP5ACTX9 uid50551	NC_015064	NC_015057, NC_015058, NC_015059, NC_015060, NC_015065
18	Acidobacterium capsulatum ATCC 51196 uid59127	NC_012483	-
19	Acidothermus cellulolyticus 11B uid58501	NC_008578	-
20	Acidovorax JS42 uid58427	NC_008782	NC_008765, NC_008766
21	Acidovorax avenae ATCC 19860 uid42497	NC_015138	-
22	Acidovorax citrulli AAC00 1 uid58429	NC_008752	-
23	Acidovorax ebreus TPSY uid59233	NC_011992	-
24	Aciduliprofundum boonei T469 uid43333	NC_013926	-
25	Acinetobacter ADP1 uid61597	NC_005966	-
26	Acinetobacter baumannii AB0057 uid59083	NC_011586	NC_011585
27	Acinetobacter baumannii AB307 0294 uid59271	NC_011595	-
28	Acinetobacter baumannii ACICU uid58765	NC_010611	NC_010605, NC_010606

Continued on next page

A list of chromosomes and plasmids analysed in the main text.

	Genome Name	Chromosome Accession IDs	Plasmid Accession IDs
29	Acinetobacter baumannii ATCC 17978 uid58731	NC_009085	NC_009083, NC_009084
30	Acinetobacter baumannii AYE uid61637	NC_010410	NC_010401, NC_010402, NC_010404
31	Acinetobacter baumannii SDF uid61601	NC_010400	NC_010396, NC_010398
32	Acinetobacter oleivorans DR1 uid50119	NC_014259	-
33	Actinobacillus pleuropneumoniae serovar 3 JL03 uid58891	NC_010278	-
34	Actinobacillus pleuropneumoniae serovar 5b L20 uid58789	NC_009053	-
35	Actinobacillus pleuropneumoniae serovar 7 AP76 uid59231	NC_010939	NC_010941, NC_010942
36	Actinobacillus succinogenes 130Z uid58247	NC_009655	-
37	Actinosynnema mirum DSM 43827 uid58951	NC_013093	-
38	Aerococcus urinae ACS 120 V Col10a uid64757	NC_015278	-
39	Aeromonas hydrophila ATCC 7966 uid58617	NC_008570	-
40	Aeromonas salmonicida A449 uid58631	NC_009348	NC_004923, NC_004924, NC_009349, NC_009350
41	Aeromonas veronii B565 uid66323	NC_015424	-
42	Aeropyrum pernix K1 uid57757	NC_000854	-
43	Aggregatibacter actinomycetemcomitans D11S 1 uid41333	NC_013416	NC_013438, NC_013597, NC_014629
44	Aggregatibacter aphrophilus NJ8700 uid59407	NC_012913	-
45	Agrobacterium H13 3 uid63403	NC_015183, NC_015508	NC_015184
46	Agrobacterium radiobacter K84 uid58269	NC_011983, NC_011985	NC_011987, NC_011990, NC_011994
47	Agrobacterium tumefaciens C58 uid57865	NC_003062, NC_003063	NC_003064, NC_003065
48	Agrobacterium vitis S4 uid58249	NC_011988, NC_011989	NC_011981, NC_011982, NC_011984, NC_011986, NC_011991
49	Akkermansia muciniphila ATCC BAA 835 uid58985	NC_010655	-
50	Alcanivorax borkumensis SK2 uid58169	NC_008260	-
51	Alicycliphilus denitrificans BC uid49953	NC_014910	NC_014908, NC_014911
52	Alicycliphilus denitrificans K601 uid66307	NC_015422	NC_015423
53	Alicyclobacillus acidocaldarius DSM 446 uid59199	NC_013205	NC_013206, NC_013207, NC_013208
54	Aliivibrio salmonicida LFI1238 uid59251	NC_011312, NC_011313	NC_011311, NC_011314
55	Alkalilimnicola ehrlichii MLHE 1 uid58467	NC_008340	-
56	Alkaliphilus metalliredigens QYMF uid58171	NC_009633	-
57	Alkaliphilus oremlandii OhILAs uid58495	NC_009922	-
58	Allochromatium vinosum DSM 180 uid46083	NC_013851	NC_013852, NC_013862
59	Alteromonas SN2 uid67349	NC_015554	-
60	Alteromonas macleodii Deep ecotype uid58251	NC_011138	-

Continued on next page

A list of chromosomes and plasmids analysed in the main text.

	Genome Name	Chromosome Accession IDs	Plasmid Accession IDs
61	Aminobacterium colombiense DSM 12261 uid47083	NC_014011	-
62	Ammonifex degensii KC4 uid41053	NC_013385	NC_013386
63	Amycolatopsis mediterranei U32 uid50565	NC_014318	-
64	Amycolicicoccus subflavus DQS3 9A1 uid67253	NC_015564	NC_015560, NC_015561
65	Anabaena variabilis ATCC 29413 uid58043	NC_007413	NC_007410, NC_007411, NC_007412
66	Anaerococcus prevotii DSM 20548 uid59219	NC_013171	NC_013164
67	Anaerolinea thermophila UNI 1 uid62245	NC_014960	-
68	Anaeromyxobacter Fw109 5 uid58755	NC_009675	-
69	Anaeromyxobacter K uid58953	NC_011145	-
70	Anaeromyxobacter dehalogenans 2CP 1 uid58989	NC_011891	-
71	Anaeromyxobacter dehalogenans 2CP C uid58135	NC_007760	-
72	Anaplasma centrale Israel uid42155	NC_013532	-
73	Anaplasma marginale Florida uid58577	NC_012026	-
74	Anaplasma marginale Maries uid57629	NC_004842	-
75	Anaplasma phagocytophilum HZ uid57951	NC_007797	-
76	Anoxybacillus flavithermus WK1 uid59135	NC_011567	-
77	Aquifex aeolicus VF5 uid57765	NC_000918	NC_001880
78	Arcanobacterium haemolyticum DSM 20595 uid49489	NC_014218	-
79	Archaeoglobus fulgidus DSM 4304 uid57717	NC_000917	-
80	Archaeoglobus profundus DSM 5631 uid43493	NC_013741	-
81	Archaeoglobus veneficus SNP6 uid65269	NC_015320	-
82	Arcobacter butzleri RM4018 uid58557	NC_009850	-
83	Arcobacter nitrofigilis DSM 7299 uid49001	NC_014166	-
84	Aromatoleum aromaticum EbN1 uid58231	NC_006513	NC_006823, NC_006824
85	Arthrobacter FB24 uid58141	NC_008541	NC_008537, NC_008538, NC_008539
86	Arthrobacter arilaitensis Re117 uid53509	NC_014550	NC_014549
87	Arthrobacter aurescens TC1 uid58109	NC_008711	NC_008712, NC_008713
88	Arthrobacter chlorophenolicus A6 uid58969	NC_011886	NC_011879, NC_011881
89	Arthrobacter phenanthrenivorans Sphe3 uid63629	NC_015145	NC_015146, NC_015147
90	Aster yellows witches broom phytoplasma AYWB uid58297	NC_007716	NC_007718
91	Asticcacaulis excentricus CB 48 uid55641	NC_014816, NC_014817	NC_014818, NC_014819
92	Atopobium parvulum DSM 20469 uid59195	NC_013203	-
93	Azoarcus BH72 uid61603	NC_008702	-
94	Azorhizobium caulinodans ORS 571 uid58905	NC_009937	-
95	Azospirillum B510 uid46085	NC_013854	NC_013855, NC_013856, NC_013857, NC_013858, NC_013859, NC_013860

Continued on next page

A list of chromosomes and plasmids analysed in the main text.

	Genome Name	Chromosome Accession IDs	Plasmid Accession IDs
96	Azotobacter vinelandii DJ uid57597	NC_012560	-
97	Bacillus amyloliquefaciens DSM 7 uid53535	NC_014551	-
98	Bacillus amyloliquefaciens FZB42 uid58271	NC_009725	-
99	Bacillus anthracis A0248 uid59385	NC_012659	NC_012655, NC_012656
100	Bacillus anthracis Ames Ancestor uid58083	NC_007530	NC_007322, NC_007323
101	Bacillus anthracis Ames uid57909	NC_003997	-
102	Bacillus anthracis CDC 684 uid59303	NC_012581	NC_012577, NC_012579
103	Bacillus anthracis Sterne uid58091	NC_005945	-
104	Bacillus atrophaeus 1942 uid59887	NC_014639	-
105	Bacillus cellulosilyticus DSM 2522 uid43329	NC_014829	-
106	Bacillus cereus 03BB102 uid59299	NC_012472	NC_012473
107	Bacillus cereus AH187 uid58753	NC_011658	NC_011655, NC_011656
108	Bacillus cereus AH820 uid58751	NC_011773	NC_011771, NC_011777
109	Bacillus cereus ATCC 10987 uid57673	NC_003909	NC_005707
110	Bacillus cereus ATCC 14579 uid57975	NC_004722	NC_004721
111	Bacillus cereus B4264 uid58757	NC_011725	-
112	Bacillus cereus E33L uid58103	NC_006274	NC_007103, NC_007105, NC_007107
113	Bacillus cereus G9842 uid58759	NC_011772	NC_011774, NC_011775
114	Bacillus cereus Q1 uid58529	NC_011969	NC_011971, NC_011973
115	Bacillus cereus biovar anthracis CI uid50615	NC_014335	NC_014331, NC_014332
116	Bacillus clausii KSM K16 uid58237	NC_006582	-
117	Bacillus coagulans 2 6 uid68053	NC_015634	-
118	Bacillus coagulans 36D1 uid54335	NC_016023	-
119	Bacillus cytotoxicus NVH 391 98 uid58317	NC_009674	-
120	Bacillus halodurans C 125 uid57791	NC_002570	-
121	Bacillus licheniformis ATCC 14580 uid58097	NC_006270	-
122	Bacillus megaterium DSM319 uid48371	NC_014103	-
123	Bacillus megaterium QM B1551 uid15862	NC_014019	NC_004604, NC_014023, NC_014025, NC_014031
124	Bacillus pseudofirmus OF4 uid45847	NC_013791	NC_013792, NC_013793
125	Bacillus pumilus SAFR 032 uid59017	NC_009848	-
126	Bacillus selenitireducens MLS10 uid49513	NC_014219	-
127	Bacillus subtilis 168 uid57675	NC_000964	-
128	Bacillus subtilis BSn5 uid62463	NC_014976	-
129	Bacillus subtilis spizizenii TU B 10 uid73967	NC_016047	-
130	Bacillus subtilis spizizenii W23 uid51879	NC_014479	-
131	Bacillus thuringiensis Al Hakam uid58795	NC_008600	NC_008598
132	Bacillus thuringiensis BMB171 uid49135	NC_014171	NC_014172
133	Bacillus thuringiensis serovar konkukian 97 27 uid58089	NC_005957	NC_006578

Continued on next page

A list of chromosomes and plasmids analysed in the main text.

	Genome Name	Chromosome Accession IDs	Plasmid Accession IDs
134	Bacillus weihenstephanensis KBAB4 uid58315	NC_010184	NC_010180, NC_010181, NC_010182, NC_010183
135	Bacteroides fragilis NCTC 9343 uid57639	NC_003228	NC_006873
136	Bacteroides fragilis YCH46 uid58195	NC_006347	NC_006297
137	Bacteroides helcogenes P 36 108 uid62135	NC_014933	-
138	Bacteroides salanitronis DSM 18170 uid63269	NC_015164	NC_015165, NC_015168
139	Bacteroides thetaiotaomicron VPI 5482 uid62913	NC_004663	NC_004703
140	Bacteroides vulgatus ATCC 8482 uid58253	NC_009614	-
141	Bartonella bacilliformis KC583 uid58533	NC_008783	-
142	Bartonella clarridgeiae 73 uid62131	NC_014932	-
143	Bartonella grahamii as4aup uid59405	NC_012846	NC_012847
144	Bartonella henselae Houston 1 uid57745	NC_005956	-
145	Bartonella quintana Toulouse uid57635	NC_005955	-
146	Bartonella tribocorum CIP 105476 uid59129	NC_010161	NC_010160
147	Baumannia cicadellinicola Hc Homalodisca coagulata uid58111	NC_007984	-
148	Bdellovibrio bacteriovorus HD100 uid61595	NC_005363	-
149	Beijerinckia indica ATCC 9039 uid59057	NC_010581	NC_010578, NC_010580
150	Beutenbergia cavernae DSM 12333 uid59047	NC_012669	-
151	Bifidobacterium adolescentis ATCC 15703 uid58559	NC_008618	-
152	Bifidobacterium animalis lactis AD011 uid58911	NC_011835	-
153	Bifidobacterium animalis lactis Bl 04 uid59359	NC_012814	-
154	Bifidobacterium animalis lactis DSM 10140 uid59357	NC_012815	-
155	Bifidobacterium bifidum PRL2010 uid59883	NC_014638	-
156	Bifidobacterium bifidum S17 uid59545	NC_014616	-
157	Bifidobacterium dentium Bd1 uid43091	NC_013714	-
158	Bifidobacterium longum BBMN68 uid60163	NC_014656	-
159	Bifidobacterium longum DJO10A uid58833	NC_010816	NC_004252
160	Bifidobacterium longum JCM 1217 uid62695	NC_015067	-
161	Bifidobacterium longum JDM301 uid49131	NC_014169	-
162	Bifidobacterium longum NCC2705 uid57939	NC_004307	-
163	Bifidobacterium longum infantis 157F uid62693	NC_015052	-
164	Bifidobacterium longum infantis ATCC 15697 uid58677	NC_011593	-
165	Blattabacterium Blattella germanica Bge uid41533	NC_013454	-
166	Blattabacterium Mastotermes darwiniensis MADAR uid77127	NC_016146	-
167	Blattabacterium Periplaneta americana BPLAN uid41287	NC_013418	-
168	Bordetella avium 197N uid61563	NC_010645	-
169	Bordetella bronchiseptica RB50 uid57613	NC_002927	-
170	Bordetella parapertussis 12822 uid57615	NC_002928	-

Continued on next page

A list of chromosomes and plasmids analysed in the main text.

	Genome Name	Chromosome Accession IDs	Plasmid Accession IDs
171	Bordetella pertussis Tohama I uid57617	NC_002929	-
172	Bordetella petrii DSM 12804 uid61631	NC_010170	-
173	Borrelia afzelii PKo uid58653	NC_008277	NC_008274, NC_008565, NC_008566, NC_008567, NC_008569, NT_167350
174	Borrelia bissettii DN127 uid71231	NC_015921	NC_015907, NC_015908, NC_015910, NC_015920, NC_015922
175	Borrelia burgdorferi B31 uid57581	NC_001318	NC_000948, NC_000949, NC_000950, NC_000951, NC_000952, NC_000953, NC_000954, NC_000956, NC_001850, NC_001852, NC_001853, NC_001854, NC_001856, NC_001903
176	Borrelia burgdorferi ZS7 uid59429	NC_011728	NC_011720, NC_011724, NC_011779, NC_011783
177	Borrelia duttonii Ly uid58791	NC_011229	NC_011245, NC_011247, NC_011256, NC_011257, NC_011259, NC_011261, NC_011262
178	Borrelia garinii PBi uid58125	NC_006156	NC_006128
179	Borrelia hermsii DAH uid59225	NC_010673	-
180	Borrelia recurrentis A1 uid58793	NC_011244	NC_011246, NC_011252
181	Borrelia turicatae 91E135 uid58311	NC_008710	-
182	Brachybacterium faecium DSM 4810 uid58649	NC_013172	-
183	Brachyspira hyodysenteriae WA1 uid59291	NC_012225	NC_012226
184	Brachyspira murdochii DSM 12563 uid48819	NC_014150	-
185	Brachyspira pilosicoli 95 1000 uid50609	NC_014330	-
186	Bradyrhizobium BTAi1 uid58505	NC_009485	NC_009475
187	Bradyrhizobium ORS 278 uid58941	NC_009445	-
188	Bradyrhizobium japonicum USDA 110 uid57599	NC_004463	-
189	Brevibacillus brevis NBRC 100599 uid59175	NC_012491	-
190	Brevundimonas subvibrioides ATCC 15264 uid42117	NC_014375	-
191	Brucella abortus S19 uid58873	NC_010740, NC_010742	-
192	Brucella abortus bv 1 9 941 uid58019	NC_006932, NC_006933	-
193	Brucella canis ATCC 23365 uid59009	NC_010103, NC_010104	-
194	Brucella melitensis ATCC 23457 uid59241	NC_012441, NC_012442	-
195	Brucella melitensis biovar Abortus 2308 uid62937	NC_007618, NC_007624	-
196	Brucella melitensis bv 1 16M uid57735	NC_003317, NC_003318	-

Continued on next page

A list of chromosomes and plasmids analysed in the main text.

	Genome Name	Chromosome Accession IDs	Plasmid Accession IDs
197	Brucella microti CCM 4915 uid59319	NC_013118, NC_013119	-
198	Brucella ovis ATCC 25840 uid58113	NC_009504, NC_009505	-
199	Brucella pinnipedialis B2 94 uid71131	NC_015857, NC_015858	-
200	Brucella suis 1330 uid57927	NC_004310, NC_004311	-
201	Brucella suis ATCC 23445 uid59015	NC_010167, NC_010169	-
202	Buchnera aphidicola 5A Acyrthosiphon pisum uid59285	NC_011833	-
203	Buchnera aphidicola APS Acyrthosiphon pisum uid57805	NC_002528	NC_002252, NC_002253
204	Buchnera aphidicola Bp Baizongia pistaciae uid57827	NC_004545	-
205	Buchnera aphidicola Cc Cinara cedri uid58579	NC_008513	NC_011878
206	Buchnera aphidicola Cinara tujafilina uid68101	NC_015662	-
207	Buchnera aphidicola Sg Schizaphis graminum uid57913	NC_004061	-
208	Buchnera aphidicola Tuc7 Acyrthosiphon pisum uid59283	NC_011834	-
209	Burkholderia 383 uid58073	NC_007509, NC_007510, NC_007511	-
210	Burkholderia CCGE1001 uid42975	NC_015136, NC_015137	-
211	Burkholderia CCGE1002 uid42523	NC_014117, NC_014118, NC_014119	NC_014120
212	Burkholderia CCGE1003 uid46253	NC_014539, NC_014540	-
213	Burkholderia ambifaria AMMD uid58303	NC_008390, NC_008391, NC_008392	NC_008385
214	Burkholderia ambifaria MC40 6 uid58701	NC_010551, NC_010552, NC_010557	NC_010553
215	Burkholderia cenocepacia AU 1054 uid58371	NC_008060, NC_008061, NC_008062	-
216	Burkholderia cenocepacia HI2424 uid58369	NC_008542, NC_008543, NC_008544	NC_008545
217	Burkholderia cenocepacia J2315 uid57953	NC_011000, NC_011001, NC_011002	NC_011003
218	Burkholderia cenocepacia MC0 3 uid58769	NC_010508, NC_010512, NC_010515	-
219	Burkholderia gladioli BSR3 uid66301	NC_015376, NC_015381	NC_015377, NC_015378, NC_015382, NC_015383
220	Burkholderia glumae BGR1 uid59397	NC_012721, NC_012724	NC_012718, NC_012720, NC_012723, NC_012725
221	Burkholderia mallei ATCC 23344 uid57725	NC_006348, NC_006349	-
222	Burkholderia mallei NCTC 10229 uid58383	NC_008835, NC_008836	-
223	Burkholderia mallei NCTC 10247 uid58385	NC_009079, NC_009080	-
224	Burkholderia mallei SAVP1 uid58387	NC_008784, NC_008785	-
225	Burkholderia multivorans ATCC 17616 uid58697	NC_010084, NC_010086, NC_010087	NC_010070

Continued on next page

A list of chromosomes and plasmids analysed in the main text.

	Genome Name	Chromosome Accession IDs	Plasmid Accession IDs
226	Burkholderia multivorans ATCC 17616 uid58909	NC_010801, NC_010804, NC_010805	NC_010802
227	Burkholderia phymatum STM815 uid58699	NC_010622, NC_010623	NC_010625, NC_010627
228	Burkholderia phytofirmans PsJN uid58729	NC_010676, NC_010681	NC_010679
229	Burkholderia pseudomallei 1106a uid58515	NC_009076, NC_009078	-
230	Burkholderia pseudomallei 1710b uid58391	NC_007434, NC_007435	-
231	Burkholderia pseudomallei 668 uid58389	NC_009074, NC_009075	-
232	Burkholderia pseudomallei K96243 uid57733	NC_006350, NC_006351	-
233	Burkholderia pseudomallei MSHR346 uid29855	NC_012695	-
234	Burkholderia rhizoxinica HKI 454 uid60487	NC_014722	NC_014718, NC_014723
235	Burkholderia thailandensis E264 uid58081	NC_007650, NC_007651	-
236	Burkholderia vietnamiensis G4 uid58075	NC_009254, NC_009255, NC_009256	NC_009226, NC_009227, NC_009228, NC_009229, NC_009230
237	Burkholderia xenovorans LB400 uid57823	NC_007951, NC_007952, NC_007953	-
238	Butyrivibrio proteoclasticus B316 uid51489	NC_014387, NC_014388	NC_014389, NC_014390
239	Caldicellulosiruptor bescii DSM 6725 uid59201	NC_012034	NC_012036
240	Caldicellulosiruptor hydrothermalis 108 uid60157	NC_014652	-
241	Caldicellulosiruptor kristjanssonii 177R1B uid60393	NC_014721	NC_014719
242	Caldicellulosiruptor kronotskyensis 2002 uid60491	NC_014720	-
243	Caldicellulosiruptor lactoaceticus 6A uid60575	NC_015949	-
244	Caldicellulosiruptor obsidiansis OB47 uid51501	NC_014392	-
245	Caldicellulosiruptor owensensis OL uid60165	NC_014657	-
246	Caldicellulosiruptor saccharolyticus DSM 8903 uid58289	NC_009437	-
247	Calditerrivibrio nitroreducens DSM 19672 uid60821	NC_014758	NC_014749
248	Caldivirga maquilingensis IC 167 uid58711	NC_009954	-
249	Campylobacter concisus 13826 uid58667	NC_009802	NC_009795
250	Campylobacter curvus 525 92 uid58669	NC_009715	-
251	Campylobacter fetus 82 40 uid58545	NC_008599	-
252	Campylobacter hominis ATCC BAA 381 uid58981	NC_009714	-
253	Campylobacter jejuni 81 176 uid58503	NC_008787	NC_008770, NC_008790
254	Campylobacter jejuni 81116 uid58771	NC_009839	-
255	Campylobacter jejuni ICDCCJ07001 uid61249	NC_014802	NC_014801
256	Campylobacter jejuni NCTC 11168 ATCC 700819 uid57587	NC_002163	-
257	Campylobacter jejuni RM1221 uid57899	NC_003912	-
258	Campylobacter jejuni doylei 269 97 uid58671	NC_009707	-
259	Campylobacter lari RM2100 uid58115	NC_012039	NC_012040

Continued on next page

A list of chromosomes and plasmids analysed in the main text.

	Genome Name	Chromosome Accession IDs	Plasmid Accession IDs
260	Candidatus Accumulibacter phosphatis clade IIA UW 1 uid59207	NC_013194	NC_013190, NC_013191, NC_013193
261	Candidatus Amoebophilus asiaticus 5a2 uid58963	NC_010830	-
262	Candidatus Arthromitus SFB mouse Japan uid71379	NC_015913	-
263	Candidatus Arthromitus SFB rat Yit uid73425	NC_016012	-
264	Candidatus Azobacteroides pseudotrichonymphae genomovar CFP2 uid59163	NC_011565	NC_011561, NC_011562, NC_011564
265	Candidatus Blochmannia floridanus uid57999	NC_005061	-
266	Candidatus Blochmannia pennsylvanicus BPEN uid58329	NC_007292	-
267	Candidatus Blochmannia vafer BVAF uid62083	NC_014909	-
268	Candidatus Carsonella ruddii uid58773	NC_008512	-
269	Candidatus Chloracidobacterium thermophilum B uid73587	NC_016024, NC_016025	-
270	Candidatus Cloacamonas acidaminovorans uid62959	NS_000195	-
271	Candidatus Desulforudis audaxviator MP104C uid59067	NC_010424	-
272	Candidatus Hamiltonella defensa 5AT Acyrthosiphon pisum uid59289	NC_012751	NC_012752
273	Candidatus Hodgkinia cicadicola Dsem uid59311	NC_012960	-
274	Candidatus Korarchaeum cryptofilum OPF8 uid58601	NC_010482	-
275	Candidatus Koribacter versatilis Ellin345 uid58479	NC_008009	-
276	Candidatus Liberibacter asiaticus psy62 uid59227	NC_012985	-
277	Candidatus Liberibacter solanacearum CLso ZC1 uid61245	NC_014774	-
278	Candidatus Midichloria mitochondrii IricVA uid68687	NC_015722	-
279	Candidatus Moranella endobia PCIT uid68739	NC_015735	-
280	Candidatus Nitrospira defluvii uid51175	NC_014355	-
281	Candidatus Pelagibacter IMCC9063 uid66305	NC_015380	-
282	Candidatus Pelagibacter ubique HTCC1062 uid58401	NC_007205	-
283	Candidatus Phytoplasma australiense uid61641	NC_010544	-
284	Candidatus Phytoplasma mali uid59087	NC_011047	-
285	Candidatus Protochlamydia amoebophila UWE25 uid58079	NC_005861	-
286	Candidatus Puniceispirillum marinum IMCC1322 uid47081	NC_014010	-
287	Candidatus Riesia pediculicola USDA uid46841	NC_014109	NC_013962
288	Candidatus Ruthia magnifica Cm Calyptogena magnifica uid58645	NC_008610	-
289	Candidatus Solibacter usitatus Ellin6076 uid58139	NC_008536	-
290	Candidatus Sulcia muelleri CARI uid52535	NC_014499	-
291	Candidatus Sulcia muelleri DMIN uid47075	NC_014004	-

Continued on next page

A list of chromosomes and plasmids analysed in the main text.

	Genome Name	Chromosome Accession IDs	Plasmid Accession IDs
292	Candidatus Sulcia muelleri GWSS uid58943	NC_010118	-
293	Candidatus Sulcia muelleri SMDSEM uid59393	NC_013123	-
294	Candidatus Tremblaya princeps PCIT uid68741	NC_015736	-
295	Candidatus Vesicomyosocius okutanii HA uid59427	NC_009465	-
296	Candidatus Zinderia insecticola CARI uid52459	NC_014497	-
297	Capnocytophaga canimorsus Cc5 uid70727	NC_015846	-
298	Capnocytophaga ochracea DSM 7271 uid59197	NC_013162	-
299	Carboxydothermus hydrogenoformans Z 2901 uid57821	NC_007503	-
300	Carnobacterium 17 4 uid65789	NC_015391	NC_015390
301	Catenulispora acidiphila DSM 44928 uid59077	NC_013131	-
302	Caulobacter K31 uid58551	NC_010338	NC_010333, NC_010335
303	Caulobacter crescentus CB15 uid57891	NC_002696	-
304	Caulobacter crescentus NA1000 uid59307	NC_011916	-
305	Caulobacter segnis ATCC 21756 uid41709	NC_014100	-
306	Cellulomonas fimi ATCC 484 uid66779	NC_015514	-
307	Cellulomonas flavigena DSM 20109 uid48821	NC_014151	-
308	Cellulophaga algicola DSM 14237 uid62159	NC_014934	-
309	Cellulophaga lytica DSM 7489 uid63401	NC_015167	-
310	Cellvibrio gilvus ATCC 13127 uid68143	NC_015671	-
311	Cellvibrio japonicus Ueda107 uid59139	NC_010995	-
312	Cenarchaeum symbiosum A uid61411	NC_014820	-
313	Chelativorans BNC1 uid58069	NC_008254	NC_008242, NC_008243, NC_008244
314	Chitinophaga pinensis DSM 2588 uid59113	NC_013132	-
315	Chlamydia muridarum Nigg uid57785	NC_002620	NC_002182
316	Chlamydia trachomatis 434 Bu uid61633	NC_010287	-
317	Chlamydia trachomatis A HAR 13 uid58333	NC_007429	NC_007430
318	Chlamydia trachomatis B Jali20 OT uid59351	NC_012686	-
319	Chlamydia trachomatis B TZ1A828 OT uid59349	NC_012687	-
320	Chlamydia trachomatis D UW 3 CX uid57637	NC_000117	-
321	Chlamydia trachomatis L2b UCH 1 proctitis uid61635	NC_010280	-
322	Chlamydia trachomatis L2c uid68843	NC_015744	-
323	Chlamydophila abortus S26 3 uid57963	NC_004552	-
324	Chlamydophila caviae GPIC uid57783	NC_003361	-
325	Chlamydophila felis Fe C 56 uid57971	NC_007899	NC_007900
326	Chlamydophila pecorum E58 uid66295	NC_015408	-
327	Chlamydophila pneumoniae AR39 uid57809	NC_002179	-
328	Chlamydophila pneumoniae CWL029 uid57811	NC_000922	-
329	Chlamydophila pneumoniae J138 uid57829	NC_002491	-
330	Chlamydophila pneumoniae TW 183 uid57997	NC_005043	-

Continued on next page

A list of chromosomes and plasmids analysed in the main text.

	Genome Name	Chromosome Accession IDs	Plasmid Accession IDs
331	Chlamydophila psittaci 6BC uid63621	NC_015470	NC_015217
332	Chlorobaculum parvum NCIB 8327 uid59185	NC_011027	-
333	Chlorobium chlorochromatii CaD3 uid58375	NC_007514	-
334	Chlorobium limicola DSM 245 uid58127	NC_010803	-
335	Chlorobium luteolum DSM 273 uid58175	NC_007512	-
336	Chlorobium phaeobacteroides BS1 uid58131	NC_010831	-
337	Chlorobium phaeobacteroides DSM 266 uid58133	NC_008639	-
338	Chlorobium phaeovibrioides DSM 265 uid58129	NC_009337	-
339	Chlorobium tepidum TLS uid57897	NC_002932	-
340	Chloroflexus Y 400 fl uid59085	NC_012032	-
341	Chloroflexus aggregans DSM 9485 uid58621	NC_011831	-
342	Chloroflexus aurantiacus J 10 fl uid57657	NC_010175	-
343	Chloroherpeton thalassium ATCC 35110 uid59187	NC_011026	-
344	Chromobacterium violaceum ATCC 12472 uid58001	NC_005085	-
345	Chromohalobacter salexigens DSM 3043 uid62921	NC_007963	-
346	Citrobacter koseri ATCC BAA 895 uid58143	NC_009792	NC_009794
347	Citrobacter rodentium ICC168 uid43089	NC_013716	NC_013717, NC_013718
348	Clavibacter michiganensis NCPPB 382 uid61625	NC_009480	NC_009478, NC_009479
349	Clavibacter michiganensis sepedonicus uid61577	NC_010407	NC_010399, NC_010408
350	Clostridiales genomosp BVAB3 UPII9 5 uid46219	NC_013895	-
351	Clostridium SY8519 uid68705	NC_015737	-
352	Clostridium acetobutylicum ATCC 824 uid57677	NC_003030	NC_001988
353	Clostridium acetobutylicum DSM 1731 uid68293	NC_015687	NC_015686
354	Clostridium beijerinckii NCIMB 8052 uid58137	NC_009617	-
355	Clostridium botulinum A ATCC 19397 uid58927	NC_009697	-
356	Clostridium botulinum A ATCC 3502 uid61579	NC_009495	NC_009496
357	Clostridium botulinum A Hall uid58931	NC_009698	-
358	Clostridium botulinum A2 Kyoto uid59229	NC_012563	-
359	Clostridium botulinum A3 Loch Maree uid59149	NC_010520	NC_010418
360	Clostridium botulinum B Eklund 17B uid59159	NC_010674	NC_010680
361	Clostridium botulinum B1 Okra uid59147	NC_010516	NC_010379
362	Clostridium botulinum BKT015925 uid66203	NC_015425	NC_015417, NC_015418, NC_015419, NC_015426, NC_015427
363	Clostridium botulinum Ba4 657 uid59173	NC_012658	NC_012654
364	Clostridium botulinum E3 Alaska E43 uid59157	NC_010723	-
365	Clostridium botulinum F Langeland uid58929	NC_009699	NC_009700
366	Clostridium cellulolyticum H10 uid58709	NC_011898	-
367	Clostridium cellulovorans 743B uid51503	NC_014393	-
368	Clostridium difficile 630 uid57679	NC_009089	-

Continued on next page

A list of chromosomes and plasmids analysed in the main text.

	Genome Name	Chromosome Accession IDs	Plasmid Accession IDs
369	Clostridium difficile CD196 uid41017	NC_013315	-
370	Clostridium difficile R20291 uid40921	NC_013316	-
371	Clostridium kluyveri DSM 555 uid58885	NC_009706	NC_009466
372	Clostridium kluyveri NBRC 12016 uid59369	NC_011837	NC_011836
373	Clostridium lentocellum DSM 5427 uid49117	NC_015275	-
374	Clostridium ljungdahlii DSM 13528 uid50583	NC_014328	-
375	Clostridium novyi NT uid58643	NC_008593	-
376	Clostridium perfringens 13 uid57681	NC_003366	NC_003042
377	Clostridium perfringens ATCC 13124 uid57901	NC_008261	-
378	Clostridium perfringens SM101 uid58117	NC_008262	NC_008263, NC_008264, NC_008265
379	Clostridium phytofermentans ISDg uid58519	NC_010001	-
380	Clostridium saccharolyticum WM1 uid51419	NC_014376	-
381	Clostridium sticklandii DSM 519 uid59585	NC_014614	-
382	Clostridium tetani E88 uid57683	NC_004557	NC_004565
383	Clostridium thermocellum ATCC 27405 uid57917	NC_009012	-
384	Collimonas fungivorans Ter331 uid70793	NC_015856	-
385	Colwellia psychrerythraea 34H uid57855	NC_003910	-
386	Comamonas testosteroni CNB 2 uid62961	NC_013446	-
387	Conexibacter woesei DSM 14684 uid43467	NC_013739	-
388	Coprothermobacter proteolyticus DSM 5265 uid59253	NC_011295	-
389	Coraliomargarita akajimensis DSM 45221 uid47079	NC_014008	-
390	Coriobacterium glomerans PW2 uid65787	NC_015389	-
391	Corynebacterium aurimucosum ATCC 700975 uid59409	NC_012590	NC_010813
392	Corynebacterium diphtheriae NCTC 13129 uid57691	NC_002935	-
393	Corynebacterium efficiens YS 314 uid62905	NC_004369	NC_004319, NC_004320
394	Corynebacterium glutamicum ATCC 13032 uid57905	NC_003450	-
395	Corynebacterium glutamicum ATCC 13032 uid61611	NC_006958	-
396	Corynebacterium glutamicum R uid58897	NC_009342	-
397	Corynebacterium jeikeium K411 uid58399	NC_007164	NC_003080
398	Corynebacterium kroppenstedtii DSM 44385 uid59411	NC_012704	-
399	Corynebacterium pseudotuberculosis FRC41 uid50585	NC_014329	-
400	Corynebacterium resistens DSM 45100 uid50555	NC_015673	-
401	Corynebacterium ulcerans BR AD22 uid68291	NC_015683	-
402	Corynebacterium urealyticum DSM 7109 uid61639	NC_010545	-
403	Corynebacterium variabile DSM 44702 uid62003	NC_015859	-
404	Coxiella burnetii CbuG Q212 uid58893	NC_011527	-
405	Coxiella burnetii CbuK Q154 uid58895	NC_011528	NC_011526
406	Coxiella burnetii Dugway 5J108 111 uid58629	NC_009727	NC_009726
407	Coxiella burnetii RSA 331 uid58637	NC_010117	NC_010115

Continued on next page

A list of chromosomes and plasmids analysed in the main text.

	Genome Name	Chromosome Accession IDs	Plasmid Accession IDs
408	Coxiella burnetii RSA 493 uid57631	NC_002971	NC_004704
409	Croceibacter atlanticus HTCC2559 uid49661	NC_014230	-
410	Cronobacter sakazakii ATCC BAA 894 uid58145	NC_009778	NC_009779, NC_009780
411	Cronobacter turicensis z3032 uid40821	NC_013282	NC_013283, NC_013284, NC_013285
412	Cryptobacterium curtum DSM 15641 uid59041	NC_013170	-
413	Cupriavidus metallidurans CH34 uid57815	NC_007973	NC_007971, NC_007972, NC_007974
414	Cupriavidus necator N 1 uid68689	NC_015723, NC_015726	NC_015724, NC_015727
415	Cupriavidus taiwanensis LMG 19424 uid61615	NC_010528, NC_010530	NC_010529
416	Cyanothece ATCC 51142 uid59013	NC_010546, NC_010547	NC_010539, NC_010541, NC_010542, NC_010543
417	Cyanothece PCC 7424 uid59025	NC_011729	NC_011730, NC_011732, NC_011737, NC_011738
418	Cyanothece PCC 7425 uid59435	NC_011884	NC_011880, NC_011882, NC_011885
419	Cyanothece PCC 7822 uid52547	NC_014501	NC_014502, NC_014503, NC_014504, NC_014533, NC_014534
420	Cyanothece PCC 8801 uid59027	NC_011726	NC_011721, NC_011723, NC_011727
421	Cyanothece PCC 8802 uid59143	NC_013161	NC_013160, NC_013163, NC_013167, NC_013168
422	Cyclobacterium marinum DSM 745 uid71485	NC_015914	-
423	Cytophaga hutchinsonii ATCC 33406 uid57651	NC_008255	-
424	Dechloromonas aromatica RCB uid58025	NC_007298	-
425	Deferribacter desulfuricans SSM1 uid46653	NC_013939	NC_013940
426	Dehalococcoides BAV1 uid58477	NC_009455	-
427	Dehalococcoides CBDB1 uid58413	NC_007356	-
428	Dehalococcoides GT uid42115	NC_013890	-
429	Dehalococcoides VS uid42393	NC_013552	-
430	Dehalococcoides ethenogenes 195 uid57763	NC_002936	-
431	Dehalogenimonas lykanthroporepellens BL DC 9 uid48131	NC_014314	-
432	Deinococcus deserti VCD115 uid58615	NC_012526	NC_012527, NC_012528, NC_012529
433	Deinococcus geothermalis DSM 11300 uid58275	NC_008025	NC_008010, NC_009939
434	Deinococcus maricopensis DSM 21211 uid62225	NC_014958	-
435	Deinococcus proteolyticus MRP uid63399	NC_015161	NC_015162, NC_015163, NC_015169, NC_015170
436	Deinococcus radiodurans R1 uid57665	NC_001263, NC_001264	NC_000958, NC_000959

Continued on next page

A list of chromosomes and plasmids analysed in the main text.

	Genome Name	Chromosome Accession IDs	Plasmid Accession IDs
437	Delftia Cs1 4 uid67319	NC_015563	-
438	Delftia acidovorans SPH 1 uid58703	NC_010002	-
439	Denitrovibrio acetiphilus DSM 12809 uid46657	NC_013943	-
440	Desulfarculus baarsii DSM 2075 uid51371	NC_014365	-
441	Desulfatibacillum alkenivorans AK 01 uid58913	NC_011768	-
442	Desulfitobacterium hafniense DCB 2 uid57749	NC_011830	-
443	Desulfitobacterium hafniense Y51 uid58605	NC_007907	-
444	Desulfobacca acetoxidans DSM 11109 uid65785	NC_015388	-
445	Desulfobacterium autotrophicum HRM2 uid59061	NC_012108	NC_012109
446	Desulfobulbus propionicus DSM 2032 uid62265	NC_014972	-
447	Desulfococcus oleovorans Hxd3 uid58777	NC_009943	-
448	Desulfohalobium retbaense DSM 5692 uid59183	NC_013223	NC_013224
449	Desulfomicrobium baculatum DSM 4028 uid59217	NC_013173	-
450	Desulfotalea psychrophila LSv54 uid58153	NC_006138	NC_006139, NC_006140
451	Desulfotomaculum acetoxidans DSM 771 uid59109	NC_013216	-
452	Desulfotomaculum carboxydivorans CO 1 SRB uid67317	NC_015565	-
453	Desulfotomaculum kuznetsovii DSM 6115 uid67357	NC_015573	-
454	Desulfotomaculum reducens MI 1 uid58277	NC_009253	-
455	Desulfotomaculum ruminis DSM 2154 uid67507	NC_015589	-
456	Desulfovibrio aespoeensis Aspo 2 uid42613	NC_014844	-
457	Desulfovibrio alaskensis G20 uid57941	NC_007519	-
458	Desulfovibrio desulfuricans ATCC 27774 uid59213	NC_011883	-
459	Desulfovibrio magneticus RS 1 uid59309	NC_012796	NC_012795, NC_012797
460	Desulfovibrio salexigens DSM 2638 uid59223	NC_012881	-
461	Desulfovibrio vulgaris DP4 uid58679	NC_008751	NC_008741
462	Desulfovibrio vulgaris Hildenborough uid57645	NC_002937	NC_005863
463	Desulfovibrio vulgaris Miyazaki F uid59089	NC_011769	-
464	Desulfurispirillum indicum S5 uid45897	NC_014836	-
465	Desulfurivibrio alkaliphilus AHT2 uid49487	NC_014216	-
466	Desulfurobacterium thermolithotrophum DSM 11699 uid63405	NC_015185	-
467	Desulfurococcus kamchatkensis 1221n uid59133	NC_011766	-
468	Desulfurococcus mucosus DSM 2162 uid62227	NC_014961	-
469	Dichelobacter nodosus VCS1703A uid57643	NC_009446	-
470	Dickeya dadantii 3937 uid52537	NC_014500	-
471	Dickeya dadantii Ech586 uid42519	NC_013592	-
472	Dickeya dadantii Ech703 uid59363	NC_012880	-
473	Dickeya zeae Ech1591 uid59297	NC_012912	-
474	Dictyoglomus thermophilum H 6 12 uid59439	NC_011297	-
475	Dictyoglomus turgidum DSM 6724 uid59177	NC_011661	-

Continued on next page

A list of chromosomes and plasmids analysed in the main text.

	Genome Name	Chromosome Accession IDs	Plasmid Accession IDs
476	Dinoroseobacter shibae DFL 12 uid58707	NC_009952	NC_009955, NC_009956, NC_009957, NC_009958, NC_009959
477	Dyadobacter fermentans DSM 18053 uid59049	NC_013037	-
478	Edwardsiella ictaluri 93 146 uid59403	NC_012779	-
479	Edwardsiella tarda EIB202 uid41819	NC_013508	NC_013509
480	Eggerthella YY7918 uid68707	NC_015738	-
481	Eggerthella lenta DSM 2243 uid59079	NC_013204	-
482	Ehrlichia canis Jake uid58071	NC_007354	-
483	Ehrlichia chaffeensis Arkansas uid57933	NC_007799	-
484	Ehrlichia ruminantium Gardel uid58245	NC_006831	-
485	Ehrlichia ruminantium Welgevonden uid58013	NC_005295	-
486	Ehrlichia ruminantium Welgevonden uid58243	NC_006832	-
487	Elusimicrobium minutum Pei191 uid58949	NC_010644	-
488	Enterobacter 638 uid58727	NC_009436	NC_009425
489	Enterobacter aerogenes KCTC 2190 uid68103	NC_015663	-
490	Enterobacter asburiae LF7a uid72793	NC_015968	NC_015963, NC_015969
491	Enterobacter cloacae ATCC 13047 uid48363	NC_014121	NC_014107, NC_014108
492	Enterobacter cloacae SCF1 uid59969	NC_014618	-
493	Enterococcus faecalis V583 uid57669	NC_004668	NC_004669, NC_004670, NC_004671
494	Erwinia amylovora ATCC 49946 uid46943	NC_013971	NC_013972, NC_013973
495	Erwinia amylovora CFBP1430 uid46839	NC_013961	NC_013957
496	Erwinia billingiae Eb661 uid50547	NC_014306	NC_014304, NC_014305
497	Erwinia pyrifoliae Ep1 96 uid40659	NC_012214	NC_013263, NC_013264, NC_013265
498	Erwinia tasmaniensis Et1 99 uid59029	NC_010694	NC_010693, NC_010695, NC_010696, NC_010697, NC_010699
499	Erysipelothrix rhusiopathiae Fujisawa uid68021	NC_015601	-
500	Erythrobacter litoralis HTCC2594 uid58299	NC_007722	-
501	Escherichia coli 536 uid58531	NC_008253	-
502	Escherichia coli 55989 uid59383	NC_011748	-
503	Escherichia coli APEC O1 uid58623	NC_008563	NC_009837, NC_009838
504	Escherichia coli ATCC 8739 uid58783	NC_010468	-
505	Escherichia coli B REL606 uid58803	NC_012967	-
506	Escherichia coli BL21 Gold DE3 pLysS AG uid59245	NC_012947	-
507	Escherichia coli BW2952 uid59391	NC_012759	-
508	Escherichia coli CFT073 uid57915	NC_004431	-

Continued on next page

A list of chromosomes and plasmids analysed in the main text.

	Genome Name	Chromosome Accession IDs	Plasmid Accession IDs
509	Escherichia coli E24377A uid58395	NC_009801	NC_009786, NC_009787, NC_009788, NC_009789, NC_009790
510	Escherichia coli ED1a uid59379	NC_011745	-
511	Escherichia coli HS uid58393	NC_009800	-
512	Escherichia coli IAI39 uid59381	NC_011750	-
513	Escherichia coli K 12 substr DH10B uid58979	NC_010473	-
514	Escherichia coli K 12 substr MG1655 uid57779	NC_000913	-
515	Escherichia coli O103 H2 12009 uid41013	NC_013353	NC_013354
516	Escherichia coli O111 H 11128 uid41023	NC_013364	NC_013365, NC_013366, NC_013370
517	Escherichia coli O127 H6 E2348 69 uid59343	NC_011601	NC_011602, NC_011603
518	Escherichia coli O157 H7 EC4115 uid59091	NC_011353	NC_011350, NC_011351
519	Escherichia coli O157 H7 EDL933 uid57831	NC_002655	NC_007414
520	Escherichia coli O157 H7 Sakai uid57781	NC_002695	NC_002128
521	Escherichia coli O157 H7 TW14359 uid59235	NC_013008	NC_013010
522	Escherichia coli O26 H11 11368 uid41021	NC_013361	NC_013362, NC_013369
523	Escherichia coli O55 H7 CB9615 uid46655	NC_013941	NC_013942
524	Escherichia coli S88 uid62979	NC_011742	NC_011747
525	Escherichia coli SE11 uid59425	NC_011415	NC_011413, NC_011416, NC_011419
526	Escherichia coli SMS 3 5 uid58919	NC_010498	NC_010488
527	Escherichia coli UMN026 uid62981	NC_011751	NC_011739, NC_011749
528	Escherichia coli UTI89 uid58541	NC_007946	NC_007941
529	Escherichia fergusonii ATCC 35469 uid59375	NC_011740	NC_011743
530	Ethanoligenens harbinense YUAN 3 uid46255	NC_014828	-
531	Eubacterium eligens ATCC 27750 uid59171	NC_012778	NC_012780, NC_012782
532	Eubacterium limosum KIST612 uid59777	NC_014624	-
533	Eubacterium rectale ATCC 33656 uid59169	NC_012781	-
534	Exiguobacterium AT1b uid59093	NC_012673	-
535	Exiguobacterium sibiricum 255 15 uid58053	NC_010556	-
536	Ferrimonas balearica DSM 9799 uid53371	NC_014541	-
537	Ferroglobus placidus DSM 10642 uid40863	NC_013849	-
538	Fervidobacterium nodosum Rt17 B1 uid58625	NC_009718	-
539	Fibrobacter succinogenes S85 uid41169	NC_013410	-
540	Finegoldia magna ATCC 29328 uid58867	NC_010376	NC_010371
541	Flavobacteriaceae bacterium 3519 10 uid59413	NC_013062	-
542	Flavobacteriales bacterium HTCC2170 uid51877	NC_014472	-
543	Flavobacterium branchiophilum FL 15 uid73421	NC_016001	-
544	Flavobacterium johnsoniae UW101 uid58493	NC_009441	-

Continued on next page

A list of chromosomes and plasmids analysed in the main text.

	Genome Name	Chromosome Accession IDs	Plasmid Accession IDs
545	Flavobacterium psychrophilum JIP02 86 uid61627	NC_009613	-
546	Flexistipes sinusarabici DSM 4947 uid68147	NC_015672	-
547	Fluviicola taffensis DSM 16823 uid65271	NC_015321	-
548	Francisella TX077308 uid68321	NC_015696	-
549	Francisella novicida U112 uid58499	NC_008601	-
550	Francisella philomiragia ATCC 25017 uid59105	NC_010336	-
551	Francisella tularensis FSC198 uid58693	NC_008245	-
552	Francisella tularensis SCHU S4 uid57589	NC_006570	-
553	Francisella tularensis WY96 3418 uid58811	NC_009257	-
554	Francisella tularensis holarctica FTNF002 00 uid58999	NC_009749	-
555	Francisella tularensis holarctica LVS uid58595	NC_007880	-
556	Francisella tularensis holarctica OSU18 uid58687	NC_008369	-
557	Francisella tularensis mediasiatica FSC147 uid58939	NC_010677	-
558	Frankia CcI3 uid58397	NC_007777	-
559	Frankia EAN1pec uid58367	NC_009921	-
560	Frankia EuI1c uid42615	NC_014666	-
561	Frankia alni ACN14a uid58695	NC_008278	-
562	Frankia symbiont of Datisca glomerata uid46257	NC_015656	NC_015664
563	Fusobacterium nucleatum ATCC 25586 uid57885	NC_003454	-
564	Gallibacterium anatis UMN179 uid66567	NC_015460	-
565	Gallionella capsiferriformans ES 2 uid51505	NC_014394	-
566	Gardnerella vaginalis 409 05 uid43211	NC_013721	-
567	Gardnerella vaginalis ATCC 14019 uid55487	NC_014644	-
568	Gemmatimonas aurantiaca T 27 uid58813	NC_012489	-
569	Geobacillus C56 T3 uid49467	NC_014206	-
570	Geobacillus WCH70 uid59045	NC_012793	NC_012790, NC_012794
571	Geobacillus Y4 1MC1 uid55779	NC_014650	NC_014651
572	Geobacillus Y412MC52 uid55381	NC_014915	NC_014916
573	Geobacillus Y412MC61 uid41171	NC_013411	NC_013412
574	Geobacillus kaustophilus HTA426 uid58227	NC_006510	NC_006509
575	Geobacillus thermodenitrificans NG80 2 uid58829	NC_009328	NC_009329
576	Geobacillus thermoglucosidasius C56 YS93 uid48129	NC_015660	NC_015661, NC_015665
577	Geobacter FRC 32 uid58543	NC_011979	-
578	Geobacter M18 uid55771	NC_014973	-
579	Geobacter M21 uid59037	NC_012918	-
580	Geobacter bemidjiensis Bem uid58749	NC_011146	-
581	Geobacter lovleyi SZ uid58713	NC_010814	NC_010815
582	Geobacter sulfurreducens PCA uid57743	NC_002939	-
583	Geobacter uraniireducens Rf4 uid58475	NC_009483	-
584	Geodermatophilus obscurus DSM 43160 uid43725	NC_013757	-

Continued on next page

A list of chromosomes and plasmids analysed in the main text.

	Genome Name	Chromosome Accession IDs	Plasmid Accession IDs
585	Glaciecola 4H 3 7 YE 5 uid66595	NC_015497	NC_015498
586	Glaciecola nitratireducens FR1064 uid73759	NC_016041	-
587	Gloeobacter violaceus PCC 7421 uid58011	NC_005125	-
588	Gluconacetobacter diazotrophicus PAl 5 uid59075	NC_011365	NC_011367
589	Gluconacetobacter diazotrophicus PAl 5 uid61587	NC_010125	NC_010123, NC_010124
590	Gluconacetobacter xylinus NBRC 3288 uid46523	NC_016027	NC_016021, NC_016028, NC_016030, NC_016037
591	Gluconobacter oxydans 621H uid58239	NC_006677	NC_006672, NC_006673, NC_006674, NC_006675
592	Gordonia bronchialis DSM 43247 uid41403	NC_013441	NC_013442
593	Gramella forsetii KT0803 uid58881	NC_008571	-
594	Granulibacter bethesdensis CGDNIH1 uid58661	NC_008343	-
595	Haemophilus ducreyi 35000HP uid57625	NC_002940	-
596	Haemophilus influenzae 86 028NP uid58093	NC_007146	-
597	Haemophilus influenzae F3031 uid62123	NC_014920	-
598	Haemophilus influenzae F3047 uid62097	NC_014922	-
599	Haemophilus influenzae PittEE uid58591	NC_009566	-
600	Haemophilus influenzae PittGG uid58593	NC_009567	-
601	Haemophilus influenzae Rd KW20 uid57771	NC_000907	-
602	Haemophilus parainfluenzae T3T1 uid72801	NC_015964	-
603	Haemophilus parasuis SH0165 uid59273	NC_011852	-
604	Haemophilus somnus 129PT uid57929	NC_008309	-
605	Haemophilus somnus 2336 uid57979	NC_010519	-
606	Hahella chejuensis KCTC 2396 uid58483	NC_007645	-
607	Halalkalicoccus jeotgali B3 uid50305	NC_014297	NC_014298, NC_014299, NC_014300, NC_014301, NC_014302
608	Halanaerobium hydrogeniformans uid60191	NC_014654	-
609	Haliangium ochraceum DSM 14365 uid41425	NC_013440	-
610	Haliscomenobacter hydrossis DSM 1100 uid66777	NC_015510	NC_015511, NC_015512, NC_015513
611	Haloarcula hispanica ATCC 33960 uid72475	NC_015943, NC_015948	NC_015944
612	Haloarcula marismortui ATCC 43049 uid57719	NC_006396, NC_006397	NC_006389, NC_006390, NC_006392, NC_006393, NC_006394, NC_006395
613	Halobacterium NRC 1 uid57769	NC_002607	NC_001869, NC_002608
614	Halobacterium salinarum R1 uid61571	NC_010364	NC_010366, NC_010367, NC_010368, NC_010369
615	Haloferax volcanii DS2 uid46845	NC_013967	NC_013964, NC_013966, NC_013968

Continued on next page

A list of chromosomes and plasmids analysed in the main text.

	Genome Name	Chromosome Accession IDs	Plasmid Accession IDs
616	Halogeometricum borinquense DSM 11551 uid54919	NC_014729	NC_014731, NC_014732, NC_014735, NC_014736, NC_014737
617	Halomicrobium mukohataei DSM 12286 uid59107	NC_013202	NC_013201
618	Halomonas elongata DSM 2581 uid52781	NC_014532	-
619	Halopiger xanaduensis SH 6 uid68105	NC_015666	NC_015658, NC_015659, NC_015667
620	Haloquadratum walsbyi DSM 16790 uid58673	NC_008212	NC_008213
621	Halorhabdus utahensis DSM 12940 uid59189	NC_013158	-
622	Halorhodospira halophila SL1 uid58473	NC_008789	-
623	Halorubrum lacusprofundi ATCC 49239 uid58807	NC_012028, NC_012029	NC_012030
624	Haloterrigena turkmenica DSM 5511 uid43501	NC_013743	NC_013744, NC_013745, NC_013746, NC_013747, NC_013748, NC_013749
625	Halothermothrix orenii H 168 uid58585	NC_011899	-
626	Halothiobacillus neapolitanus c2 uid41317	NC_013422	-
627	Helicobacter acinonychis Sheeba uid58685	NC_008229	-
628	Helicobacter bizzozeronii CIII 1 uid68141	NC_015674	NC_015670
629	Helicobacter felis ATCC 49179 uid61409	NC_014810	-
630	Helicobacter hepaticus ATCC 51449 uid57737	NC_004917	-
631	Helicobacter mustelae 12198 uid46647	NC_013949	-
632	Helicobacter pylori 26695 uid57787	NC_000915	-
633	Helicobacter pylori B38 uid59415	NC_012973	-
634	Helicobacter pylori B8 uid49873	NC_014256	-
635	Helicobacter pylori G27 uid59305	NC_011333	NC_011334
636	Helicobacter pylori HPAG1 uid58517	NC_008086	NC_008087
637	Helicobacter pylori J99 uid57789	NC_000921	-
638	Helicobacter pylori P12 uid59327	NC_011498	NC_011499
639	Helicobacter pylori PeCan4 uid53539	NC_014555	NC_014556
640	Helicobacter pylori SJM180 uid53541	NC_014560	-
641	Helicobacter pylori Shi470 uid59165	NC_010698	-
642	Heliobacterium modesticaldum Ice1 uid58279	NC_010337	-
643	Herbaspirillum seropedicae SmR1 uid50427	NC_014323	-
644	Herminiimonas arsenicoxydans uid58291	NC_009138	-
645	Herpetosiphon aurantiacus DSM 785 uid58599	NC_009972	NC_009973, NC_009974
646	Hippea maritima DSM 10411 uid65267	NC_015318	-
647	Hirschia baltica ATCC 49814 uid59365	NC_012982	NC_012983
648	Hydrogenobacter thermophilus TK 6 uid45927	NC_013799	-
649	Hydrogenobaculum Y04AAS1 uid58857	NC_011126	-
650	Hyperthermus butylicus DSM 5456 uid57755	NC_008818	-

Continued on next page

A list of chromosomes and plasmids analysed in the main text.

	Genome Name	Chromosome Accession IDs	Plasmid Accession IDs
651	Hyphomicrobium MC1 uid68453	NC_015717	-
652	Hyphomicrobium denitrificans ATCC 51888 uid50325	NC_014313	-
653	Hyphomonas neptunium ATCC 15444 uid58433	NC_008358	-
654	Idiomarina loihiensis L2TR uid58087	NC_006512	-
655	Ignicoccus hospitalis KIN4 I uid58365	NC_009776	-
656	Ignisphaera aggregans DSM 17230 uid51875	NC_014471	-
657	Ilyobacter polytropus DSM 2926 uid59769	NC_014632	NC_014633, NC_014634
658	Intrasporangium calvum DSM 43043 uid61729	NC_014830	-
659	Isoptericola variabilis 225 uid67501	NC_015588	-
660	Isosphaera pallida ATCC 43644 uid62207	NC_014962	NC_014957
661	Jannaschia CCS1 uid58147	NC_007802	NC_007801
662	Janthinobacterium Marseille uid58603	NC_009659	-
663	Jonesia denitrificans DSM 20603 uid59053	NC_013174	-
664	Kangiella koreensis DSM 16069 uid59209	NC_013166	-
665	Ketogulonicigenium vulgare Y25 uid59581	NC_014625	NC_014621, NC_014626
666	Kineococcus radiotolerans SRS30216 uid58067	NC_009664	NC_009660, NC_009806
667	Kitasatospora setae KM 6054 uid77027	NC_016109	-
668	Klebsiella pneumoniae 342 uid59145	NC_011283	NC_011281, NC_011282
669	Klebsiella pneumoniae MGH 78578 uid57619	NC_009648	NC_009649, NC_009650, NC_009651, NC_009653
670	Klebsiella pneumoniae NTUH K2044 uid59073	NC_012731	NC_006625
671	Klebsiella variicola At 22 uid42113	NC_013850	-
672	Kocuria rhizophila DC2201 uid59099	NC_010617	-
673	Kosmotoga olearia TBF 19 5 1 uid59205	NC_012785	-
674	Kribbella flavida DSM 17836 uid43465	NC_013729	-
675	Krokinobacter 4H 3 7 5 uid66593	NC_015496	-
676	Kyrpidia tusciae DSM 2912 uid48361	NC_014098	-
677	Kytococcus sedentarius DSM 20547 uid59071	NC_013169	-
678	Lacinutrix 5H 3 7 4 uid68067	NC_015638	-
679	Lactobacillus acidophilus 30SC uid63605	NC_015214	NC_015218
680	Lactobacillus acidophilus NCFM uid57685	NC_006814	-
681	Lactobacillus amylovorus GRL 1112 uid61179	NC_014724	NC_015319, NC_015322
682	Lactobacillus brevis ATCC 367 uid57989	NC_008497	NC_008498, NC_008499
683	Lactobacillus buchneri NRRL B 30929 uid66205	NC_015428	NC_015420, NC_015421, NC_015429
684	Lactobacillus casei ATCC 334 uid57985	NC_008526	NC_008502
685	Lactobacillus casei BL23 uid59237	NC_010999	-
686	Lactobacillus casei Zhang uid50673	NC_014334	NC_011352
687	Lactobacillus crispatus ST1 uid48359	NC_014106	-

Continued on next page

A list of chromosomes and plasmids analysed in the main text.

	Genome Name	Chromosome Accession IDs	Plasmid Accession IDs
688	Lactobacillus delbrueckii bulgaricus ATCC 11842 uid58647	NC_008054	-
689	Lactobacillus delbrueckii bulgaricus ATCC BAA 365 uid57987	NC_008529	-
690	Lactobacillus delbrueckii bulgaricus ND02 uid60621	NC_014727	-
691	Lactobacillus fermentum IFO 3956 uid58865	NC_010610	-
692	Lactobacillus gasseri ATCC 33323 uid57687	NC_008530	-
693	Lactobacillus helveticus DPC 4571 uid58761	NC_010080	-
694	Lactobacillus johnsonii FI9785 uid41735	NC_013504	NC_013505
695	Lactobacillus johnsonii NCC 533 uid58029	NC_005362	-
696	Lactobacillus kefiranofaciens ZW3 uid67985	NC_015602	NC_015598, NC_015603
697	Lactobacillus plantarum JDM1 uid59361	NC_012984	-
698	Lactobacillus plantarum ST III uid53537	NC_014554	NC_014558
699	Lactobacillus plantarum WCFS1 uid62911	-	NC_006377
700	Lactobacillus reuteri DSM 20016 uid58471	NC_009513	-
701	Lactobacillus reuteri JCM 1112 uid58875	NC_010609	-
702	Lactobacillus reuteri SD2112 uid55357	NC_015697	NC_015698, NC_015700, NC_015701
703	Lactobacillus rhamnosus GG uid59313	NC_013198	-
704	Lactobacillus rhamnosus Lc 705 uid59315	NC_013199	NC_013200
705	Lactobacillus ruminis ATCC 27782 uid73417	NC_015975	-
706	Lactobacillus sakei 23K uid58281	NC_007576	-
707	Lactobacillus salivarius UCC118 uid58233	NC_007929	NC_006529, NC_006530, NC_007930
708	Lactobacillus sanfranciscensis TMW 1 1304 uid72937	NC_015978	NC_015979, NC_015980
709	Lactococcus garvieae ATCC 49156 uid73413	NC_015930	-
710	Lactococcus lactis Il1403 uid57671	NC_002662	-
711	Lactococcus lactis KF147 uid42831	NC_013656	NC_013657
712	Lactococcus lactis cremoris MG1363 uid58837	NC_009004	-
713	Lactococcus lactis cremoris SK11 uid57983	NC_008527	NC_008503, NC_008504, NC_008505, NC_008506, NC_008507
714	Laribacter hongkongensis HLHK9 uid59265	NC_012559	-
715	Lawsonia intracellularis PHE MN1 00 uid61575	NC_008011	NC_008012, NC_008013, NC_008014
716	Leadbetterella byssophila DSM 17132 uid60161	NC_014655	-
717	Legionella longbeachae NSW150 uid46099	NC_013861	NC_014544
718	Legionella pneumophila 2300 99 Alcoy uid48801	NC_014125	-
719	Legionella pneumophila Corby uid58733	NC_009494	-
720	Legionella pneumophila Lens uid58209	NC_006369	NC_006366

Continued on next page

A list of chromosomes and plasmids analysed in the main text.

	Genome Name	Chromosome Accession IDs	Plasmid Accession IDs
721	Legionella pneumophila Paris uid58211	NC_006368	NC_006365
722	Legionella pneumophila Philadelphia 1 uid57609	NC_002942	-
723	Leifsonia xyli CTCB07 uid57759	NC_006087	-
724	Leptospira biflexa serovar Patoc 1 Ames uid58511	-	NC_010846
725	Leptospira biflexa serovar Patoc 1 Paris uid58993	-	NC_010844
726	Leptospira borgpetersenii serovar Hardjo bovis JB197 uid58509	NC_008510, NC_008511	-
727	Leptospira borgpetersenii serovar Hardjo bovis L550 uid58507	NC_008508, NC_008509	-
728	Leptospira interrogans serovar Copenhageni Fiocruz L1 130 uid58065	NC_005823, NC_005824	-
729	Leptospira interrogans serovar Lai 56601 uid57881	NC_004342, NC_004343	-
730	Leptothrix cholodnii SP 6 uid58971	NC_010524	-
731	Leptotrichia buccalis C 1013 b uid59211	NC_013192	-
732	Leuconostoc C2 uid68743	NC_015734	-
733	Leuconostoc citreum KM20 uid58481	NC_010471	NC_010466, NC_010467, NC_010469, NC_010470
734	Leuconostoc gasicomitatum LMG 18811 uid50385	NC_014319	-
735	Leuconostoc kimchii IMSNU 11154 uid48589	NC_014136	NC_014131, NC_014132, NC_014133, NC_014134
736	Leuconostoc mesenteroides ATCC 8293 uid57919	NC_008531	NC_008496
737	Listeria innocua Clip11262 uid61567	NC_003212	NC_003383
738	Listeria ivanovii PAM 55 uid73473	NC_016011	-
739	Listeria monocytogenes 08 5923 uid43727	NC_013768	-
740	Listeria monocytogenes Clip80459 uid59317	NC_012488	-
741	Listeria monocytogenes EGD e uid61583	NC_003210	-
742	Listeria monocytogenes HCC23 uid59203	NC_011660	-
743	Listeria monocytogenes serotype 4b F2365 uid57689	NC_002973	-
744	Listeria monocytogenes uid43671	NC_013766	NC_013767
745	Listeria seeligeri serovar 1 2b SLCC3954 uid46215	NC_013891	-
746	Listeria welshimeri serovar 6b SLCC5334 uid61605	NC_008555	-
747	Lysinibacillus sphaericus C3 41 uid58945	NC_010382	NC_010381
748	Macrococcus caseolyticus JCSC5402 uid59003	NC_011999	NC_011995, NC_011996
749	Magnetococcus MC 1 uid57833	NC_008576	-
750	Magnetospirillum magneticum AMB 1 uid58527	NC_007626	-
751	Mahella australiensis 50 1 BON uid66917	NC_015520	-
752	Mannheimia succiniciproducens MBEL55E uid58197	NC_006300	-
753	Maricaulis maris MCS10 uid58689	NC_008347	-
754	Marinithermus hydrothermalis DSM 14884 uid65783	NC_015387	-
755	Marinobacter aquaeolei VT8 uid59419	NC_008740	NC_008738, NC_008739

Continued on next page

A list of chromosomes and plasmids analysed in the main text.

	Genome Name	Chromosome Accession IDs	Plasmid Accession IDs
756	Marinomonas MWYL1 uid58715	NC_009654	-
757	Marinomonas mediterranea MMB 1 uid64753	NC_015276	-
758	Marinomonas posidonica IVIA Po 181 uid67323	NC_015559	-
759	Marivirga tractuosa DSM 4126 uid60837	NC_014759	NC_014750
760	Megasphaera elsdenii DSM 20460 uid71135	NC_015873	-
761	Meiothermus ruber DSM 1279 uid46661	NC_013946	-
762	Meiothermus silvanus DSM 9946 uid49485	NC_014212	NC_014213, NC_014214
763	Melissococcus plutonius ATCC 35311 uid66803	NC_015516	NC_015517
764	Mesoplasma florum L1 uid58055	NC_006055	-
765	Mesorhizobium ciceri biovar biserrulae WSM1271 uid62101	-	NC_014918
766	Mesorhizobium loti MAFF303099 uid57601	NC_002678	NC_002679, NC_002682
767	Mesorhizobium opportunistum WSM2075 uid40861	NC_015675	-
768	Metallosphaera cuprina Ar 4 uid66329	NC_015435	-
769	Metallosphaera sedula DSM 5348 uid58717	NC_009440	-
770	Methanobacterium AL 21 uid63623	NC_015216	-
771	Methanobacterium SWAN 1 uid67359	NC_015574	-
772	Methanobrevibacter ruminantium M1 uid45857	NC_013790	-
773	Methanobrevibacter smithii ATCC 35061 uid58827	NC_009515	-
774	Methanocaldococcus FS406 22 uid42499	NC_013887	NC_013888
775	Methanocaldococcus fervens AG86 uid59347	NC_013156	NC_013157
776	Methanocaldococcus infernus ME uid48803	NC_014122	-
777	Methanocaldococcus jannaschii DSM 2661 uid57713	NC_000909	NC_001732, NC_001733
778	Methanocaldococcus vulcanius M7 uid41131	NC_013407	-
779	Methanocella arvoryzae MRE50 uid61623	NC_009464	-
780	Methanocella paludicola SANAE uid42887	NC_013665	-
781	Methanococcoides burtonii DSM 6242 uid58023	NC_007955	-
782	Methanococcus aeolicus Nankai 3 uid58823	NC_009635	-
783	Methanococcus maripaludis C5 uid58741	NC_009135	-
784	Methanococcus maripaludis C6 uid58947	NC_009975	-
785	Methanococcus maripaludis C7 uid58847	NC_009637	-
786	Methanococcus maripaludis S2 uid58035	NC_005791	-
787	Methanococcus maripaludis X1 uid70729	NC_015847	-
788	Methanococcus vannielii SB uid58767	NC_009634	-
789	Methanococcus voltae A3 uid49529	NC_014222	-
790	Methanocorpusculum labreanum Z uid58785	NC_008942	-
791	Methanoculleus marisnigri JR1 uid58561	NC_009051	-
792	Methanohalobium evestigatum Z 7303 uid49857	NC_014253	NC_014254
793	Methanohalophilus mahii DSM 5219 uid47313	NC_014002	-
794	Methanoplanus petrolearius DSM 11571 uid52695	NC_014507	-

Continued on next page

A list of chromosomes and plasmids analysed in the main text.

	Genome Name	Chromosome Accession IDs	Plasmid Accession IDs
795	Methanopyrus kandleri AV19 uid57883	NC_003551	-
796	Methanoregula boonei 6A8 uid58815	NC_009712	-
797	Methanosaeta concilii GP6 uid66207	NC_015416	-
798	Methanosaeta thermophila PT uid58469	NC_008553	-
799	Methanosalsum zhilinae DSM 4017 uid68249	NC_015676	-
800	Methanosarcina acetivorans C2A uid57879	NC_003552	-
801	Methanosarcina barkeri Fusaro uid57715	NC_007355	NC_007349
802	Methanosarcina mazei Go1 uid57893	NC_003901	-
803	Methanosphaera stadtmanae DSM 3091 uid58407	NC_007681	-
804	Methanosphaerula palustris E1 9c uid59193	NC_011832	-
805	Methanospirillum hungatei JF 1 uid58181	NC_007796	-
806	Methanothermobacter marburgensis Marburg uid51637	NC_014408	-
807	Methanothermobacter thermautotrophicus Delta H uid57877	NC_000916	-
808	Methanothermococcus okinawensis IH1 uid51535	NC_015636	NC_015632
809	Methanothermus fervidus DSM 2088 uid60167	NC_014658	-
810	Methanotorris igneus Kol 5 uid67321	NC_015562	-
811	Methylacidiphilum infernorum V4 uid59161	NC_010794	-
812	Methylibium petroleiphilum PM1 uid58085	NC_008825	NC_008826
813	Methylobacillus flagellatus KT uid58049	NC_007947	-
814	Methylobacterium 4 46 uid58843	NC_010511	NC_010373, NC_010374
815	Methylobacterium chloromethanicum CM4 uid58933	NC_011757	NC_011758, NC_011760
816	Methylobacterium extorquens AM1 uid57605	NC_012808	NC_012807, NC_012809, NC_012811
817	Methylobacterium extorquens DM4 uid61617	NC_012988	NC_012987, NC_012989
818	Methylobacterium extorquens PA1 uid58821	NC_010172	-
819	Methylobacterium nodulans ORS 2060 uid59023	NC_011894	NC_011887, NC_011888, NC_011892, NC_011893
820	Methylobacterium populi BJ001 uid58937	NC_010725	NC_010721, NC_010727
821	Methylobacterium radiotolerans JCM 2831 uid58845	NC_010505	NC_010502, NC_010507, NC_010509, NC_010510, NC_010514, NC_010517, NC_010518
822	Methylocella silvestris BL2 uid59433	NC_011666	-
823	Methylococcus capsulatus Bath uid57607	NC_002977	-
824	Methylomicrobium alcaliphilum uid77119	NC_016112	-
825	Methylomonas methanica MC09 uid67363	NC_015572	-
826	Methylotenera 301 uid49469	NC_014207	-
827	Methylotenera mobilis JLW8 uid59373	NC_012968	-
828	Methylovorus MP688 uid60723	NC_014733	-

Continued on next page

A list of chromosomes and plasmids analysed in the main text.

	Genome Name	Chromosome Accession IDs	Plasmid Accession IDs
829	Methylovorus glucosetrophus SIP3 4 uid59367	NC_012969	NC_012970, NC_012972
830	Micavibrio aeruginosavorus ARL 13 uid73585	NC_016026	-
831	Microbacterium testaceum StLB037 uid62789	NC_015125	-
832	Micrococcus luteus NCTC 2665 uid59033	NC_012803	-
833	Microcystis aeruginosa NIES 843 uid59101	NC_010296	-
834	Microlunatus phosphovorus NM 1 uid68055	NC_015635	-
835	Micromonospora L5 uid45895	NC_014815	-
836	Micromonospora aurantiaca ATCC 27029 uid42501	NC_014391	-
837	Mobiluncus curtisii ATCC 43063 uid49695	NC_014246	-
838	Moorella thermoacetica ATCC 39073 uid58051	NC_007644	-
839	Moraxella catarrhalis RH4 uid48809	NC_014147	-
840	Muricauda ruestringensis DSM 13258 uid72479	NC_015945	-
841	Mycobacterium JDM601 uid67369	NC_015576	-
842	Mycobacterium JLS uid58489	NC_009077	-
843	Mycobacterium KMS uid58491	NC_008705	NC_008703, NC_008704
844	Mycobacterium MCS uid58465	NC_008146	NC_008147
845	Mycobacterium abscessus ATCC 19977 uid61613	NC_010397	NC_010394
846	Mycobacterium africanum GM041182 uid68839	NC_015758	-
847	Mycobacterium avium 104 uid57693	NC_008595	-
848	Mycobacterium avium paratuberculosis K 10 uid57699	NC_002944	-
849	Mycobacterium bovis AF2122 97 uid57695	NC_002945	-
850	Mycobacterium bovis BCG Pasteur 1173P2 uid58781	NC_008769	-
851	Mycobacterium bovis BCG Tokyo 172 uid59281	NC_012207	-
852	Mycobacterium canettii CIPT 140010059 uid70731	NC_015848	-
853	Mycobacterium gilvum PYR GCK uid59421	NC_009338	NC_009339, NC_009340, NC_009341
854	Mycobacterium gilvum Spyr1 uid61403	NC_014814	NC_014811, NC_014812
855	Mycobacterium leprae Br4923 uid59293	NC_011896	-
856	Mycobacterium leprae TN uid57697	NC_002677	-
857	Mycobacterium marinum M uid59423	NC_010612	NC_010604
858	Mycobacterium smegmatis MC2 155 uid57701	NC_008596	-
859	Mycobacterium tuberculosis CDC1551 uid57775	NC_002755	-
860	Mycobacterium tuberculosis F11 uid58417	NC_009565	-
861	Mycobacterium tuberculosis H37Ra uid58853	NC_009525	-
862	Mycobacterium tuberculosis H37Rv uid57777	NC_000962	-
863	Mycobacterium tuberculosis KZN 1435 uid59069	NC_012943	-
864	Mycobacterium ulcerans Agy99 uid62939	NC_008611	NC_005916
865	Mycobacterium vanbaalenii PYR 1 uid58463	NC_008726	-
866	Mycoplasma agalactiae PG2 uid61619	NC_009497	-
867	Mycoplasma agalactiae uid46679	NC_013948	-

Continued on next page

A list of chromosomes and plasmids analysed in the main text.

	Genome Name	Chromosome Accession IDs	Plasmid Accession IDs
868	Mycoplasma arthritidis 158L3 1 uid58005	NC_011025	-
869	Mycoplasma bovis Hubei 1 uid68691	NC_015725	-
870	Mycoplasma bovis PG45 uid60859	NC_014760	-
871	Mycoplasma capricolum ATCC 27343 uid58525	NC_007633	-
872	Mycoplasma conjunctivae HRC 581 uid59325	NC_012806	-
873	Mycoplasma crocodyli MP145 uid47087	NC_014014	-
874	Mycoplasma fermentans JER uid53543	NC_014552	-
875	Mycoplasma fermentans M64 uid62099	NC_014921	-
876	Mycoplasma gallisepticum R low uid57993	NC_004829	-
877	Mycoplasma genitalium G37 uid57707	NC_000908	-
878	Mycoplasma haemofelis Langford 1 uid62461	NC_014970	-
879	Mycoplasma hominis ATCC 23114 uid41875	NC_013511	-
880	Mycoplasma hyopneumoniae 232 uid58205	NC_006360	-
881	Mycoplasma hyopneumoniae 7448 uid58039	NC_007332	-
882	Mycoplasma hyopneumoniae J uid58059	NC_007295	-
883	Mycoplasma hyorhinis HUB 1 uid51695	NC_014448	-
884	Mycoplasma leachii PG50 uid60849	NC_014751	-
885	Mycoplasma mobile 163K uid58077	NC_006908	-
886	Mycoplasma mycoides SC PG1 uid58031	NC_005364	-
887	Mycoplasma mycoides capri LC 95010 uid66189	NC_015431	-
888	Mycoplasma penetrans HF 2 uid57729	NC_004432	-
889	Mycoplasma pneumoniae M129 uid57709	NC_000912	-
890	Mycoplasma pulmonis UAB CTIP uid61569	NC_002771	-
891	Mycoplasma putrefaciens KS1 uid72481	NC_015946	-
892	Mycoplasma suis Illinois uid61897	NC_015155	-
893	Mycoplasma suis KI3806 uid63665	NC_015153	-
894	Mycoplasma synoviae 53 uid58061	NC_007294	-
895	Myxococcus fulvus HW 1 uid68443	NC_015711	-
896	Myxococcus xanthus DK 1622 uid58003	NC_008095	-
897	Nakamurella multipartita DSM 44233 uid59221	NC_013235	-
898	Nanoarchaeum equitans Kin4 M uid58009	NC_005213	-
899	Natranaerobius thermophilus JW NM WN LF uid59001	NC_010718	NC_010715, NC_010724
900	Natrialba magadii ATCC 43099 uid46245	NC_013922	NC_013923, NC_013924, NC_013925
901	Natronomonas pharaonis DSM 2160 uid58435	NC_007426	NC_007427
902	Nautilia profundicola AmH uid59345	NC_012115	-
903	Neisseria gonorrhoeae FA 1090 uid57611	NC_002946	-
904	Neisseria gonorrhoeae NCCP11945 uid59191	NC_011035	-
905	Neisseria lactamica 020 06 uid60851	NC_014752	-
906	Neisseria meningitidis 053442 uid58587	NC_010120	-

Continued on next page

A list of chromosomes and plasmids analysed in the main text.

	Genome Name	Chromosome Accession IDs	Plasmid Accession IDs
907	Neisseria meningitidis FAM18 uid57825	NC_008767	-
908	Neisseria meningitidis MC58 uid57817	NC_003112	-
909	Neisseria meningitidis Z2491 uid57819	NC_003116	-
910	Neisseria meningitidis alpha14 uid61649	NC_013016	-
911	Neorickettsia risticii Illinois uid58889	NC_013009	-
912	Neorickettsia sennetsu Miyayama uid57965	NC_007798	-
913	Nitratifractor salsuginis DSM 16511 uid62183	NC_014935	-
914	Nitratiruptor SB155 2 uid58861	NC_009662	-
915	Nitrobacter hamburgensis X14 uid58293	NC_007964	NC_007959, NC_007960, NC_007961
916	Nitrobacter winogradskyi Nb 255 uid58295	NC_007406	-
917	Nitrosococcus halophilus Nc4 uid46803	NC_013960	NC_013958
918	Nitrosococcus oceani ATCC 19707 uid58403	NC_007484	NC_007483
919	Nitrosococcus watsonii C 113 uid50331	NC_014315	NC_014316, NC_014317
920	Nitrosomonas AL212 uid55727	NC_015222	NC_015221, NC_015223
921	Nitrosomonas Is79A3 uid68745	NC_015731	-
922	Nitrosomonas europaea ATCC 19718 uid57647	NC_004757	-
923	Nitrosomonas eutropha C91 uid58363	NC_008344	NC_008341, NC_008342
924	Nitrosopumilus maritimus SCM1 uid58903	NC_010085	-
925	Nitrosospira multiformis ATCC 25196 uid58361	NC_007614	NC_007615, NC_007616, NC_007617
926	Nocardia farcinica IFM 10152 uid58203	NC_006361	NC_006362, NC_006363
927	Nocardioides JS614 uid58149	NC_008699	NC_008697
928	Nocardiopsis dassonvillei DSM 43111 uid49483	NC_014210, NC_014211	-
929	Nostoc PCC 7120 uid57803	NC_003272	NC_003240, NC_003267, NC_003270, NC_003273, NC_003276
930	Nostoc azollae 0708 uid49725	NC_014248	NC_014249, NC_014250
931	Nostoc punctiforme PCC 73102 uid57767	NC_010628	NC_010629, NC_010630, NC_010631, NC_010632, NC_010633
932	Novosphingobium PP1Y uid67383	NC_015580	NC_015579, NC_015582, NC_015583
933	Novosphingobium aromaticivorans DSM 12444 uid57747	NC_007794	NC_009426, NC_009427
934	Oceanithermus profundus DSM 14977 uid60855	NC_014761	NC_014753
935	Oceanobacillus iheyensis HTE831 uid57867	NC_004193	-
936	Ochrobactrum anthropi ATCC 49188 uid58921	NC_009667, NC_009668	NC_009669, NC_009670, NC_009671, NC_009672
937	Odoribacter splanchnicus DSM 20712 uid63397	NC_015160	-
938	Oenococcus oeni PSU 1 uid59417	NC_008528	-

Continued on next page

A list of chromosomes and plasmids analysed in the main text.

	Genome Name	Chromosome Accession IDs	Plasmid Accession IDs
939	Oligotropha carboxidovorans OM5 uid59155	NC_011386	-
940	Oligotropha carboxidovorans OM5 uid72795	NC_015684	NC_015685, NC_015689
941	Olsenella uli DSM 7084 uid51367	NC_014363	-
942	Onion yellows phytoplasma OY M uid58015	NC_005303	-
943	Opitutus terrae PB90 1 uid58965	NC_010571	-
944	Orientia tsutsugamushi Boryong uid61621	NC_009488	-
945	Orientia tsutsugamushi Ikeda uid58869	NC_010793	-
946	Oscillibacter valericigenes uid73895	NC_016048	NC_016046
947	Paenibacillus JDR 2 uid59021	NC_012914	-
948	Paenibacillus Y412MC10 uid41127	NC_013406	-
949	Paenibacillus mucilaginosus KNP414 uid68311	NC_015690	-
950	Paenibacillus polymyxa E681 uid53477	NC_014483	-
951	Paenibacillus polymyxa SC2 uid59583	NC_014622	NC_014628
952	Paludibacter propionicigenes WB4 uid60725	NC_014734	-
953	Pantoea At 9b uid55845	NC_014837	NC_014838, NC_014839, NC_014840, NC_014841, NC_014842
954	Pantoea ananatis LMG 20103 uid46807	NC_013956	-
955	Pantoea vagans C9 1 uid49871	NC_014562	NC_014258, NC_014561, NC_014563
956	Parabacteroides distasonis ATCC 8503 uid58301	NC_009615	-
957	Parachlamydia acanthamoebae UV7 uid68335	NC_015702	-
958	Paracoccus denitrificans PD1222 uid58187	NC_008686, NC_008687	NC_008688
959	Parvibaculum lavamentivorans DS 1 uid58739	NC_009719	-
960	Parvularcula bermudensis HTCC2503 uid51641	NC_014414	-
961	Pasteurella multocida Pm70 uid57627	NC_002663	-
962	Pectobacterium atrosepticum SCRI1043 uid57957	NC_004547	-
963	Pectobacterium carotovorum PC1 uid59295	NC_012917	-
964	Pectobacterium wasabiae WPP163 uid41297	NC_013421	-
965	Pediococcus pentosaceus ATCC 25745 uid57981	NC_008525	-
966	Pedobacter heparinus DSM 2366 uid59111	NC_013061	-
967	Pedobacter saltans DSM 12145 uid61349	NC_015177	-
968	Pelagibacterium halotolerans B2 uid74393	NC_016078	NC_016079
969	Pelobacter propionicus DSM 2379 uid58255	NC_008609	NC_008607, NC_008608
970	Pelodictyon phaeoclathratiforme BU 1 uid58173	NC_011060	-
971	Pelotomaculum thermopropionicum SI uid58877	NC_009454	-
972	Persephonella marina EX H1 uid58119	NC_012440	NC_012439
973	Petrotoga mobilis SJ95 uid58747	NC_010003	-
974	Phenylobacterium zucineum HLK1 uid58959	NC_011144	NC_011143
975	Photobacterium profundum SS9 uid62923	NC_006370, NC_006371	NC_005871

Continued on next page

A list of chromosomes and plasmids analysed in the main text.

	Genome Name	Chromosome Accession IDs	Plasmid Accession IDs
976	Photorhabdus asymbiotica ATCC 43949 uid59243	NC_012962	NC_012961
977	Photorhabdus luminescens laumondii TTO1 uid61593	NC_005126	-
978	Picrophilus torridus DSM 9790 uid58041	NC_005877	-
979	Pirellula staleyi DSM 6068 uid43209	NC_013720	-
980	Planctomyces brasiliensis DSM 5305 uid60583	NC_015174	-
981	Planctomyces limnophilus DSM 3776 uid48643	NC_014148	NC_014149
982	Polaromonas JS666 uid58207	NC_007948	NC_007949, NC_007950
983	Polaromonas naphthalenivorans CJ2 uid58273	NC_008781	NC_008757, NC_008758, NC_008759, NC_008760, NC_008761, NC_008762
984	Polymorphum gilvum SL003B 26A1 uid65447	NC_015259	NC_015258
985	Polynucleobacter necessarius STIR1 uid58967	NC_010531	-
986	Polynucleobacter necessarius asymbioticus QLW P1DMWA 1 uid58611	NC_009379	-
987	Porphyromonas asaccharolytica DSM 20707 uid66603	NC_015501	-
988	Porphyromonas gingivalis ATCC 33277 uid58879	NC_010729	-
989	Porphyromonas gingivalis TDC60 uid67407	NC_015571	-
990	Porphyromonas gingivalis W83 uid57641	NC_002950	-
991	Prevotella denticola F0289 uid65091	NC_015311	-
992	Prevotella melaninogenica ATCC 25845 uid51377	NC_014370, NC_014371	-
993	Prevotella ruminicola 23 uid47507	NC_014033	-
994	Prochlorococcus marinus AS9601 uid58307	NC_008816	-
995	Prochlorococcus marinus CCMP1375 uid57995	NC_005042	-
996	Prochlorococcus marinus MIT 9211 uid58309	NC_009976	-
997	Prochlorococcus marinus MIT 9215 uid58819	NC_009840	-
998	Prochlorococcus marinus MIT 9301 uid58437	NC_009091	-
999	Prochlorococcus marinus MIT 9303 uid58305	NC_008820	-
1000	Prochlorococcus marinus MIT 9312 uid58357	NC_007577	-
1001	Prochlorococcus marinus MIT 9313 uid57773	NC_005071	-
1002	Prochlorococcus marinus MIT 9515 uid58313	NC_008817	-
1003	Prochlorococcus marinus NATL1A uid58423	NC_008819	-
1004	Prochlorococcus marinus NATL2A uid58359	NC_007335	-
1005	Prochlorococcus marinus pastoris CCMP1986 uid57761	NC_005072	-
1006	Propionibacterium acnes KPA171202 uid58101	NC_006085	-
1007	Propionibacterium acnes SK137 uid48071	NC_014039	-
1008	Propionibacterium freudenreichii shermanii CIRM BIA1 uid49535	NC_014215	-
1009	Prosthecochloris aestuarii DSM 271 uid58151	NC_011059	NC_011061
1010	Proteus mirabilis HI4320 uid61599	NC_010554	NC_010555
1011	Pseudoalteromonas SM9913 uid61247	NC_014800, NC_014803	-

Continued on next page

A list of chromosomes and plasmids analysed in the main text.

	Genome Name	Chromosome Accession IDs	Plasmid Accession IDs
1012	Pseudoalteromonas atlantica T6c uid58283	NC_008228	-
1013	Pseudoalteromonas haloplanktis TAC125 uid58431	NC_007481, NC_007482	-
1014	Pseudogulbenkiania NH8B uid73423	NC_016002	-
1015	Pseudomonas aeruginosa LESB58 uid59275	NC_011770	-
1016	Pseudomonas aeruginosa PA7 uid58627	NC_009656	-
1017	Pseudomonas aeruginosa PAO1 uid57945	NC_002516	-
1018	Pseudomonas aeruginosa UCBPP PA14 uid57977	NC_008463	-
1019	Pseudomonas brassicacearum NFM421 uid66303	NC_015379	-
1020	Pseudomonas entomophila L48 uid58639	NC_008027	-
1021	Pseudomonas fluorescens Pf 5 uid57937	NC_004129	-
1022	Pseudomonas fluorescens Pf0 1 uid57591	NC_007492	-
1023	Pseudomonas fluorescens SBW25 uid158693	NC_012660	-
1024	Pseudomonas fulva 12 X uid67351	NC_015556	-
1025	Pseudomonas mendocina NK 01 uid66299	NC_015410	-
1026	Pseudomonas mendocina ymp uid58723	NC_009439	-
1027	Pseudomonas putida F1 uid58355	NC_009512	-
1028	Pseudomonas putida GB 1 uid58735	NC_010322	-
1029	Pseudomonas putida KT2440 uid57843	NC_002947	-
1030	Pseudomonas putida S16 uid68747	NC_015733	-
1031	Pseudomonas putida W619 uid58651	NC_010501	-
1032	Pseudomonas stutzeri A1501 uid58641	NC_009434	-
1033	Pseudomonas stutzeri ATCC 17588 LMG 11199 uid68749	NC_015740	-
1034	Pseudomonas syringae B728a uid57931	NC_007005	-
1035	Pseudomonas syringae phaseolicola 1448A uid58099	NC_005773	NC_007274, NC_007275
1036	Pseudomonas syringae tomato DC3000 uid57967	NC_004578	NC_004632, NC_004633
1037	Pseudonocardia dioxanivorans CB1190 uid65087	NC_015312	NC_015313, NC_015314
1038	Pseudoxanthomonas spadix BD a59 uid75113	NC_016147	-
1039	Pseudoxanthomonas suwonensis 11 1 uid62105	NC_014924	-
1040	Psychrobacter PRwf 1 uid58459	NC_009524	NC_009516
1041	Psychrobacter arcticus 273 4 uid58021	NC_007204	-
1042	Psychrobacter cryohalolentis K5 uid58373	NC_007969	NC_007968
1043	Psychromonas ingrahamii 37 uid58521	NC_008709	-
1044	Pusillimonas T7 7 uid66391	NC_015458	NC_015459
1045	Pyrobaculum aerophilum IM2 uid57727	NC_003364	-
1046	Pyrobaculum arsenaticum DSM 13514 uid58409	NC_009376	-
1047	Pyrobaculum calidifontis JCM 11548 uid58787	NC_009073	-
1048	Pyrobaculum islandicum DSM 4184 uid58635	NC_008701	-
1049	Pyrococcus NA2 uid66551	NC_015474	-
1050	Pyrococcus abyssi GE5 uid62903	NC_000868	-
1051	Pyrococcus furiosus DSM 3638 uid57873	NC_003413	-

Continued on next page

A list of chromosomes and plasmids analysed in the main text.

	Genome Name	Chromosome Accession IDs	Plasmid Accession IDs
1052	Pyrococcus horikoshii OT3 uid57753	NC_000961	-
1053	Pyrococcus yayanosii CH1 uid68281	NC_015680	-
1054	Pyrolobus fumarii 1A uid73415	NC_015931	-
1055	Rahnella Y9602 uid62715	NC_015061	NC_015062, NC_015063
1056	Ralstonia eutropha H16 uid62925	NC_008313, NC_008314	NC_005241
1057	Ralstonia eutropha JMP134 uid58047	NC_007347, NC_007348	NC_007336, NC_007337
1058	Ralstonia pickettii 12D uid58859	NC_012856, NC_012857	NC_012849, NC_012851, NC_012855
1059	Ralstonia pickettii 12J uid58737	NC_010678, NC_010682	NC_010683
1060	Ralstonia solanacearum CFBP2957 uid50545	NC_014307	-
1061	Ralstonia solanacearum GMI1000 uid57593	NC_003295	NC_003296
1062	Ralstonia solanacearum PSI07 uid50539	NC_014311	NC_014310
1063	Ramlibacter tataouinensis TTB310 uid68279	NC_015677	-
1064	Renibacterium salmoninarum ATCC 33209 uid58899	NC_010168	-
1065	Rhizobium NGR234 uid59081	NC_012587	NC_000914, NC_012586
1066	Rhizobium etli CFN 42 uid58377	NC_007761	NC_004041, NC_007762, NC_007763, NC_007764, NC_007765, NC_007766
1067	Rhizobium etli CIAT 652 uid59115	NC_010994	NC_010996, NC_010997, NC_010998
1068	Rhizobium leguminosarum bv trifolii WSM1325 uid58991	NC_012850	NC_012848, NC_012852, NC_012853, NC_012854, NC_012858
1069	Rhizobium leguminosarum bv trifolii WSM2304 uid58997	NC_011369	NC_011366, NC_011368, NC_011370, NC_011371
1070	Rhizobium leguminosarum bv viciae 3841 uid57955	NC_008380	NC_008378, NC_008379, NC_008381, NC_008382, NC_008383, NC_008384
1071	Rhodobacter capsulatus SB 1003 uid47509	NC_014034	NC_014035
1072	Rhodobacter sphaeroides 2 4 1 uid57653	NC_007493, NC_007494	NC_007488, NC_007489, NC_007490, NC_009007, NC_009008
1073	Rhodobacter sphaeroides ATCC 17025 uid58451	NC_009428	NC_009429, NC_009430, NC_009431, NC_009432, NC_009433
1074	Rhodobacter sphaeroides ATCC 17029 uid58449	NC_009049, NC_009050	NC_009040
1075	Rhodobacter sphaeroides KD131 uid59277	NC_011958, NC_011963	NC_011960, NC_011962
1076	Rhodococcus equi 103S uid60171	NC_014659	-
1077	Rhodococcus erythropolis PR4 uid59019	NC_012490	NC_007486, NC_007491

Continued on next page

A list of chromosomes and plasmids analysed in the main text.

	Genome Name	Chromosome Accession IDs	Plasmid Accession IDs
1078	Rhodococcus jostii RHA1 uid58325	NC_008268	NC_008269, NC_008270, NC_008271
1079	Rhodococcus opacus B4 uid13791	NC_012522	NC_012520, NC_012521, NC_012523
1080	Rhodoferax ferrireducens T118 uid58353	NC_007908	NC_007901
1081	Rhodomicrobium vannielii ATCC 17100 uid43247	NC_014664	-
1082	Rhodopirellula baltica SH 1 uid61589	NC_005027	-
1083	Rhodopseudomonas palustris BisA53 uid58445	NC_008435	-
1084	Rhodopseudomonas palustris BisB18 uid58443	NC_007925	-
1085	Rhodopseudomonas palustris BisB5 uid58441	NC_007958	-
1086	Rhodopseudomonas palustris CGA009 uid62901	NC_005296	NC_005297
1087	Rhodopseudomonas palustris DX 1 uid43327	NC_014834	-
1088	Rhodopseudomonas palustris HaA2 uid58439	NC_007778	-
1089	Rhodopseudomonas palustris TIE 1 uid58995	NC_011004	-
1090	Rhodospirillum centenum SW uid58805	NC_011420	-
1091	Rhodospirillum rubrum ATCC 11170 uid57655	NC_007643	NC_007641
1092	Rhodothermus marinus DSM 4252 uid41729	NC_013501	NC_013502
1093	Rhodothermus marinus SG0 5JP17 172 uid72767	NC_015966	NC_015970
1094	Rickettsia africae ESF 5 uid58799	NC_012633	NC_012634
1095	Rickettsia akari Hartford uid58161	NC_009881	-
1096	Rickettsia bellii OSU 85 389 uid58681	NC_009883	-
1097	Rickettsia bellii RML369 C uid58405	NC_007940	-
1098	Rickettsia canadensis McKiel uid58159	NC_009879	-
1099	Rickettsia conorii Malish 7 uid57633	NC_003103	-
1100	Rickettsia felis URRWXCal2 uid58331	NC_007109	NC_007110, NC_007111
1101	Rickettsia heilongjiangensis 054 uid70839	NC_015866	-
1102	Rickettsia japonica YH uid73963	NC_016050	-
1103	Rickettsia massiliae MTU5 uid58801	NC_009900	-
1104	Rickettsia peacockii Rustic uid59301	NC_012730	NC_012732
1105	Rickettsia prowazekii Madrid E uid61565	NC_000963	-
1106	Rickettsia rickettsii Iowa uid58961	NC_010263	-
1107	Rickettsia rickettsii Sheila Smith uid58027	NC_009882	-
1108	Rickettsia typhi Wilmington uid58063	NC_006142	-
1109	Riemerella anatipestifer ATCC 11845 DSM 15868 uid60727	NC_014738	-
1110	Robiginitalea biformata HTCC2501 uid58285	NC_013222	-
1111	Roseburia hominis A2 183 uid73419	NC_015977	-
1112	Roseiflexus RS 1 uid58523	NC_009523	-
1113	Roseiflexus castenholzii DSM 13941 uid58287	NC_009767	-

Continued on next page

A list of chromosomes and plasmids analysed in the main text.

	Genome Name	Chromosome Accession IDs	Plasmid Accession IDs
1114	Roseobacter denitrificans OCh 114 uid58597	NC_008209	NC_008386, NC_008387, NC_008388, NC_008389
1115	Roseobacter litoralis Och 149 uid54719	NC_015730	NC_015728, NC_015729, NC_015741
1116	Rothia dentocariosa ATCC 17931 uid49331	NC_014643	-
1117	Rothia mucilaginosa uid43093	NC_013715	-
1118	Rubrobacter xylanophilus DSM 9941 uid58057	NC_008148	-
1119	Ruegeria TM1040 uid58193	NC_008044	NC_008042, NC_008043
1120	Ruegeria pomeroyi DSS 3 uid57863	NC_003911	NC_006569
1121	Ruminococcus albus 7 uid51721	NC_014833	NC_014824, NC_014825, NC_014826, NC_014827
1122	Runella slithyformis DSM 19594 uid68317	NC_015703	NC_015693, NC_015694, NC_015695, NC_015704, NC_015705
1123	Saccharomonospora viridis DSM 43017 uid59055	NC_013159	-
1124	Saccharophagus degradans 2 40 uid57921	NC_007912	-
1125	Saccharopolyspora erythraea NRRL 2338 uid62947	NC_009142	-
1126	Salinibacter ruber DSM 13855 uid58513	NC_007677	NC_007678
1127	Salinibacter ruber M8 uid47323	NC_014032	NC_014028
1128	Salinispora arenicola CNS 205 uid58659	NC_009953	-
1129	Salinispora tropica CNB 440 uid58565	NC_009380	-
1130	Salmonella bongori NCTC 12419 uid70155	NC_015761	-
1131	Salmonella enterica arizonae serovar 62 z4 z23 RSK2980 uid58191	NC_010067	-
1132	Salmonella enterica serovar Agona SL483 uid59431	NC_011149	NC_011148
1133	Salmonella enterica serovar Choleraesuis SC B67 uid58017	NC_006905	NC_006855, NC_006856
1134	Salmonella enterica serovar Dublin CT 02021853 uid58917	NC_011205	NC_011204
1135	Salmonella enterica serovar Enteritidis P125109 uid59247	NC_011294	-
1136	Salmonella enterica serovar Gallinarum 287 91 uid59249	NC_011274	-
1137	Salmonella enterica serovar Heidelberg SL476 uid58973	NC_011083	NC_011081
1138	Salmonella enterica serovar Newport SL254 uid58831	NC_011080	NC_009140
1139	Salmonella enterica serovar Paratyphi A AKU 12601 uid59269	NC_011147	-
1140	Salmonella enterica serovar Paratyphi A ATCC 9150 uid58201	NC_006511	-
1141	Salmonella enterica serovar Paratyphi B SPB7 uid59097	NC_010102	-
1142	Salmonella enterica serovar Paratyphi C RKS4594 uid59063	NC_012125	NC_012124
1143	Salmonella enterica serovar Schwarzengrund CVM19633 uid58915	NC_011094	NC_011092

Continued on next page

A list of chromosomes and plasmids analysed in the main text.

	Genome Name	Chromosome Accession IDs	Plasmid Accession IDs
1144	Salmonella enterica serovar Typhi CT18 uid57793	NC_003198	NC_003384, NC_003385
1145	Salmonella enterica serovar Typhi Ty2 uid57973	NC_004631	-
1146	Salmonella enterica serovar Typhimurium LT2 uid57799	NC_003197	NC_003277
1147	Sanguibacter keddieii DSM 10542 uid40845	NC_013521	-
1148	Sebaldella termitidis ATCC 33386 uid41865	NC_013517	NC_013518, NC_013519
1149	Segniliparus rotundus DSM 44985 uid49049	NC_014168	-
1150	Serratia AS12 uid67315	NC_015566	-
1151	Serratia plymuthica AS9 uid67313	NC_015567	-
1152	Serratia proteamaculans 568 uid58725	NC_009832	NC_009829
1153	Shewanella ANA 3 uid58347	NC_008577	NC_008573
1154	Shewanella MR 4 uid58345	NC_008321	-
1155	Shewanella MR 7 uid58343	NC_008322	NC_008320
1156	Shewanella W3 18 1 uid58341	NC_008750	-
1157	Shewanella amazonensis SB2B uid58257	NC_008700	-
1158	Shewanella baltica OS155 uid58259	NC_009052	NC_009035, NC_009036, NC_009037
1159	Shewanella baltica OS185 uid58743	NC_009665	NC_009661
1160	Shewanella baltica OS195 uid58261	NC_009997	NC_009998, NC_009999, NC_010000
1161	Shewanella baltica OS223 uid58775	NC_011663	NC_011664, NC_011665, NC_011668
1162	Shewanella denitrificans OS217 uid58263	NC_007954	-
1163	Shewanella frigidimarina NCIMB 400 uid58265	NC_008345	-
1164	Shewanella halifaxensis HAW EB4 uid59007	NC_010334	-
1165	Shewanella loihica PV 4 uid58349	NC_009092	-
1166	Shewanella oneidensis MR 1 uid57949	NC_004347	NC_004349
1167	Shewanella pealeana ATCC 700345 uid58705	NC_009901	-
1168	Shewanella piezotolerans WP3 uid58745	NC_011566	-
1169	Shewanella putrefaciens CN 32 uid58267	NC_009438	-
1170	Shewanella sediminis HAW EB3 uid58835	NC_009831	-
1171	Shewanella violacea DSS12 uid47085	NC_014012	-
1172	Shewanella woodyi ATCC 51908 uid58721	NC_010506	-
1173	Shigella boydii CDC 3083 94 uid58415	NC_010658	NC_010657, NC_010660
1174	Shigella boydii Sb227 uid58215	NC_007613	NC_007608
1175	Shigella dysenteriae Sd197 uid58213	NC_007606	NC_007607, NC_009344
1176	Shigella flexneri 2a 2457T uid57991	NC_004741	-
1177	Shigella flexneri 2a 301 uid62907	NC_004337	NC_004851
1178	Shigella flexneri 5 8401 uid58583	NC_008258	-
1179	Shigella sonnei Ss046 uid58217	NC_007384	NC_007385, NC_009345
1180	Sideroxydans lithotrophicus ES 1 uid46801	NC_013959	-

Continued on next page

A list of chromosomes and plasmids analysed in the main text.

	Genome Name	Chromosome Accession IDs	Plasmid Accession IDs
1181	Simkania negevensis Z uid68451	NC_015713	NC_015710
1182	Sinorhizobium medicae WSM419 uid58549	NC_009636	NC_009620, NC_009621, NC_009622
1183	Sinorhizobium meliloti 1021 uid57603	NC_003047	NC_003037, NC_003078
1184	Sinorhizobium meliloti AK83 uid52607	NC_015590, NC_015591, NC_015596	NC_015592, NC_015597
1185	Slackia heliotrinireducens DSM 20476 uid59051	NC_013165	-
1186	Sodalis glossinidius morsitans uid58553	NC_007712	NC_007713, NC_007714
1187	Sorangium cellulosum So ce 56 uid61629	NC_010162	-
1188	Sphaerobacter thermophilus DSM 20745 uid41997	NC_013523, NC_013524	-
1189	Sphingobacterium 21 uid64755	NC_015277	-
1190	Sphingobium SYK 6 uid73353	NC_015976	NC_015974
1191	Sphingobium chlorophenolicum L 1 uid52597	NC_015593, NC_015594	NC_015595
1192	Sphingobium japonicum UT26S uid47077	NC_014006, NC_014013	NC_014005, NC_014007
1193	Sphingomonas wittichii RW1 uid58691	NC_009511	NC_009507, NC_009508
1194	Sphingopyxis alaskensis RB2256 uid58351	NC_008048	NC_008036
1195	Spirochaeta Buddy uid63633	NC_015152	-
1196	Spirochaeta caldaria DSM 7334 uid68753	NC_015732	-
1197	Spirochaeta coccoides DSM 17374 uid66331	NC_015436	-
1198	Spirochaeta smaragdinae DSM 11293 uid51369	NC_014364	-
1199	Spirochaeta thermophila DSM 6192 uid53037	NC_014484	-
1200	Spirosoma linguale DSM 74 uid43413	NC_013730	NC_013731, NC_013732, NC_013733, NC_013734, NC_013735, NC_013737
1201	Stackebrandtia nassauensis DSM 44728 uid46663	NC_013947	-
1202	Staphylococcus aureus COL uid57797	NC_002951	-
1203	Staphylococcus aureus ED98 uid41455	NC_013450	NC_013453
1204	Staphylococcus aureus JH1 uid58457	NC_009632	NC_009619
1205	Staphylococcus aureus JH9 uid58455	NC_009487	NC_009477
1206	Staphylococcus aureus MRSA252 uid57839	NC_002952	-
1207	Staphylococcus aureus MSSA476 uid57841	NC_002953	NC_005951
1208	Staphylococcus aureus MW2 uid57903	NC_003923	-
1209	Staphylococcus aureus Mu3 uid58817	NC_009782	-
1210	Staphylococcus aureus Mu50 uid57835	NC_002758	NC_002774
1211	Staphylococcus aureus N315 uid57837	NC_002745	NC_003140
1212	Staphylococcus aureus NCTC 8325 uid57795	NC_007795	-
1213	Staphylococcus aureus Newman uid58839	NC_009641	-
1214	Staphylococcus aureus RF122 uid57661	NC_007622	-
1215	Staphylococcus aureus USA300 FPR3757 uid58555	NC_007793	NC_007791, NC_007792
1216	Staphylococcus aureus USA300 TCH1516 uid58925	NC_010079	NC_010063

Continued on next page

A list of chromosomes and plasmids analysed in the main text.

	Genome Name	Chromosome Accession IDs	Plasmid Accession IDs
1217	Staphylococcus carnosus TM300 uid59401	NC_012121	-
1218	Staphylococcus epidermidis ATCC 12228 uid57861	NC_004461	NC_005003, NC_005004, NC_005005
1219	Staphylococcus epidermidis RP62A uid57663	NC_002976	NC_006663
1220	Staphylococcus haemolyticus JCSC1435 uid62919	NC_007168	NC_007169, NC_007171
1221	Staphylococcus lugdunensis HKU09 01 uid46233	NC_013893	-
1222	Staphylococcus pseudintermedius HKU10 03 uid62125	NC_014925	-
1223	Staphylococcus saprophyticus ATCC 15305 uid58411	NC_007350	NC_007351, NC_007352
1224	Staphylothermus hellenicus DSM 12710 uid45893	NC_014205	-
1225	Staphylothermus marinus F1 uid58719	NC_009033	-
1226	Starkeya novella DSM 506 uid48815	NC_014217	-
1227	Stenotrophomonas maltophilia JV3 uid72473	NC_015947	-
1228	Stenotrophomonas maltophilia K279a uid61647	NC_010943	-
1229	Stenotrophomonas maltophilia R551 3 uid58657	NC_011071	-
1230	Streptobacillus moniliformis DSM 12112 uid41863	NC_013515	NC_013516
1231	Streptococcus agalactiae 2603V R uid57943	NC_004116	-
1232	Streptococcus agalactiae A909 uid57935	NC_007432	-
1233	Streptococcus agalactiae NEM316 uid61585	NC_004368	-
1234	Streptococcus dysgalactiae equisimilis GGS 124 uid59103	NC_012891	-
1235	Streptococcus equi 4047 uid59259	NC_012471	-
1236	Streptococcus equi zooepidemicus MGCS10565 uid59263	NC_011134	-
1237	Streptococcus equi zooepidemicus uid59261	NC_012470	-
1238	Streptococcus gallolyticus ATCC BAA 2069 uid63617	NC_015215	NC_015219
1239	Streptococcus gallolyticus UCN34 uid46061	NC_013798	-
1240	Streptococcus gordonii Challis substr CH1 uid57667	NC_009785	-
1241	Streptococcus mitis B6 uid46097	NC_013853	-
1242	Streptococcus mutans NN2025 uid46353	NC_013928	-
1243	Streptococcus mutans UA159 uid57947	NC_004350	-
1244	Streptococcus oralis Uo5 uid65449	NC_015291	-
1245	Streptococcus parasanguinis ATCC 15912 uid49313	NC_015678	-
1246	Streptococcus parauberis KCTC 11537 uid67355	NC_015558	-
1247	Streptococcus pasteurianus ATCC 43144 uid68019	NC_015600	-
1248	Streptococcus pneumoniae 670 6B uid52533	NC_014498	-
1249	Streptococcus pneumoniae 70585 uid59125	NC_012468	-
1250	Streptococcus pneumoniae AP200 uid52453	NC_014494	-
1251	Streptococcus pneumoniae ATCC 700669 uid59287	NC_011900	-
1252	Streptococcus pneumoniae CGSP14 uid59181	NC_010582	-
1253	Streptococcus pneumoniae D39 uid58581	NC_008533	-
1254	Streptococcus pneumoniae G54 uid59167	NC_011072	-
1255	Streptococcus pneumoniae Hungary19A 6 uid59117	NC_010380	-

Continued on next page

A list of chromosomes and plasmids analysed in the main text.

	Genome Name	Chromosome Accession IDs	Plasmid Accession IDs
1256	Streptococcus pneumoniae JJA uid59121	NC_012466	-
1257	Streptococcus pneumoniae P1031 uid59123	NC_012467	-
1258	Streptococcus pneumoniae R6 uid57859	NC_003098	-
1259	Streptococcus pneumoniae TCH8431 19A uid49735	NC_014251	-
1260	Streptococcus pneumoniae TIGR4 uid57857	NC_003028	-
1261	Streptococcus pneumoniae Taiwan19F 14 uid59119	NC_012469	-
1262	Streptococcus pseudopneumoniae IS7493 uid71153	NC_015875	-
1263	Streptococcus pyogenes M1 GAS uid57845	NC_002737	-
1264	Streptococcus pyogenes MGAS10270 uid58571	NC_008022	-
1265	Streptococcus pyogenes MGAS10394 uid58105	NC_006086	-
1266	Streptococcus pyogenes MGAS10750 uid58575	NC_008024	-
1267	Streptococcus pyogenes MGAS2096 uid58573	NC_008023	-
1268	Streptococcus pyogenes MGAS315 uid57911	NC_004070	-
1269	Streptococcus pyogenes MGAS5005 uid58337	NC_007297	-
1270	Streptococcus pyogenes MGAS6180 uid58335	NC_007296	-
1271	Streptococcus pyogenes MGAS8232 uid57871	NC_003485	-
1272	Streptococcus pyogenes MGAS9429 uid58569	NC_008021	-
1273	Streptococcus pyogenes Manfredo uid57847	NC_009332	-
1274	Streptococcus pyogenes NZ131 uid59035	NC_011375	-
1275	Streptococcus pyogenes SSI 1 uid57895	NC_004606	-
1276	Streptococcus salivarius CCHSS3 uid70481	NC_015760	-
1277	Streptococcus sanguinis SK36 uid58381	NC_009009	-
1278	Streptococcus suis 05ZYH33 uid58663	NC_009442	-
1279	Streptococcus suis 98HAH33 uid58665	NC_009443	-
1280	Streptococcus suis BM407 uid59321	NC_012926	NC_012923
1281	Streptococcus suis P1 7 uid32235	NC_012925	-
1282	Streptococcus suis SC84 uid59323	NC_012924	-
1283	Streptococcus suis ST3 uid66327	NC_015433	-
1284	Streptococcus thermophilus CNRZ1066 uid58221	NC_006449	-
1285	Streptococcus thermophilus LMD 9 uid58327	NC_008532	NC_008500
1286	Streptococcus thermophilus LMG 18311 uid58219	NC_006448	-
1287	Streptococcus uberis 0140J uid57959	NC_012004	-
1288	Streptomyces SirexAA E uid72627	NC_015953	-
1289	Streptomyces avermitilis MA 4680 uid57739	NC_003155	NC_004719
1290	Streptomyces cattleya NRRL 8057 uid77117	NC_016111	NC_016113
1291	Streptomyces coelicolor A3 2 uid57801	NC_003888	NC_003903, NC_003904
1292	Streptomyces flavogriseus ATCC 33331 uid40839	NC_016114	NC_016110, NC_016115
1293	Streptomyces griseus NBRC 13350 uid58983	NC_010572	-
1294	Streptomyces scabiei 87 22 uid46531	NC_013929	-
1295	Streptomyces violaceusniger Tu 4113 uid52609	NC_015957	NC_015951, NC_015952

Continued on next page

A list of chromosomes and plasmids analysed in the main text.

	Genome Name	Chromosome Accession IDs	Plasmid Accession IDs
1296	Streptosporangium roseum DSM 43021 uid42521	NC_013595	NC_013596
1297	Sulfobacillus acidophilus TPY uid68841	NC_015757	-
1298	Sulfolobus acidocaldarius DSM 639 uid58379	NC_007181	-
1299	Sulfolobus islandicus L D 8 5 uid43679	NC_013769	NC_013770
1300	Sulfolobus islandicus L S 2 15 uid58871	NC_012589	-
1301	Sulfolobus islandicus M 14 25 uid58849	NC_012588	-
1302	Sulfolobus islandicus M 16 27 uid58851	NC_012632	-
1303	Sulfolobus islandicus M 16 4 uid58841	NC_012726	-
1304	Sulfolobus islandicus Y G 57 14 uid58923	NC_012622	-
1305	Sulfolobus islandicus Y N 15 51 uid58825	NC_012623	NC_012624
1306	Sulfolobus solfataricus P2 uid57721	NC_002754	-
1307	Sulfolobus tokodaii 7 uid57807	NC_003106	-
1308	Sulfuricurvum kujiense DSM 16994 uid60789	NC_014762	NC_014754, NC_014755, NC_014756
1309	Sulfurihydrogenibium YO3AOP1 uid58855	NC_010730	-
1310	Sulfurihydrogenibium azorense Az Fu1 uid58121	NC_012438	-
1311	Sulfurimonas autotrophica DSM 16294 uid53043	NC_014506	-
1312	Sulfurimonas denitrificans DSM 1251 uid58185	NC_007575	-
1313	Sulfurospirillum deleyianum DSM 6946 uid41861	NC_013512	-
1314	Sulfurovum NBC37 1 uid58863	NC_009663	-
1315	Symbiobacterium thermophilum IAM 14863 uid58165	NC_006177	-
1316	Synechococcus CC9311 uid58123	NC_008319	-
1317	Synechococcus CC9605 uid58319	NC_007516	-
1318	Synechococcus CC9902 uid58323	NC_007513	-
1319	Synechococcus JA 2 3B a 2 13 uid58537	NC_007776	-
1320	Synechococcus JA 3 3Ab uid58535	NC_007775	-
1321	Synechococcus PCC 7002 uid59137	NC_010475	NC_010474, NC_010477, NC_010478, NC_010479, NC_010480
1322	Synechococcus RCC307 uid61609	NC_009482	-
1323	Synechococcus WH 7803 uid61607	NC_009481	-
1324	Synechococcus WH 8102 uid61581	NC_005070	-
1325	Synechococcus elongatus PCC 6301 uid58235	NC_006576	-
1326	Synechococcus elongatus PCC 7942 uid58045	NC_007604	NC_007595
1327	Synechocystis PCC 6803 uid57659	NC_000911	NC_005229, NC_005230, NC_005231, NC_005232
1328	Syntrophobacter fumaroxidans MPOB uid58177	NC_008554	-
1329	Syntrophobotulus glycolicus DSM 8271 uid63343	NC_015172	-
1330	Syntrophomonas wolfei Goettingen uid58179	NC_008346	-
1331	Syntrophothermus lipocalidus DSM 12680 uid49527	NC_014220	-

Continued on next page

A list of chromosomes and plasmids analysed in the main text.

	Genome Name	Chromosome Accession IDs	Plasmid Accession IDs
1332	Syntrophus aciditrophicus SB uid58539	NC_007759	-
1333	Taylorella asinigenitalis MCE3 uid73771	NC_016043	-
1334	Taylorella equigenitalis MCE9 uid62103	NC_014914	-
1335	Tepidanaerobacter Re1 uid66873	NC_015519	-
1336	Teredinibacter turnerae T7901 uid59267	NC_012997	-
1337	Terriglobus saanensis SP1PR4 uid53251	NC_014963	-
1338	Tetragenococcus halophilus uid74441	NC_016052	-
1339	Thauera MZ1T uid58987	NC_011662	NC_011667
1340	Thermaerobacter marianensis DSM 12885 uid61727	NC_014831	-
1341	Thermanaerovibrio acidaminovorans DSM 6589 uid41925	NC_013522	-
1342	Thermincola potens JR uid48823	NC_014152	-
1343	Thermoanaerobacter X513 uid53065	NC_014538	-
1344	Thermoanaerobacter X514 uid58589	NC_010320	-
1345	Thermoanaerobacter brockii finnii Ako 1 uid55639	NC_014964	-
1346	Thermoanaerobacter italicus Ab9 uid46241	NC_013921	-
1347	Thermoanaerobacter mathranii A3 uid49481	NC_014209	-
1348	Thermoanaerobacter pseudethanolicus ATCC 33223 uid58339	NC_010321	-
1349	Thermoanaerobacter tengcongensis MB4 uid57813	NC_003869	-
1350	Thermoanaerobacter wiegelii Rt8 B1 uid52581	NC_015958	-
1351	Thermoanaerobacterium thermosaccharolyticum DSM 571 uid51639	NC_014410	-
1352	Thermoanaerobacterium xylanolyticum LX 11 uid63163	NC_015555	-
1353	Thermobaculum terrenum ATCC BAA 798 uid42011	NC_013525, NC_013526	-
1354	Thermobifida fusca YX uid57703	NC_007333	-
1355	Thermobispora bispora DSM 43833 uid48999	NC_014165	-
1356	Thermococcus 4557 uid70841	NC_015865	-
1357	Thermococcus AM4 uid54735	NC_016051	-
1358	Thermococcus barophilus MP uid54733	NC_014804	NC_015471
1359	Thermococcus gammatolerans EJ3 uid59389	NC_012804	-
1360	Thermococcus kodakarensis KOD1 uid58225	NC_006624	-
1361	Thermococcus onnurineus NA1 uid59043	NC_011529	-
1362	Thermococcus sibiricus MM 739 uid59399	NC_012883	-
1363	Thermocrinis albus DSM 14484 uid46231	NC_013894	-
1364	Thermodesulfatator indicus DSM 15286 uid68285	NC_015681	-
1365	Thermodesulfobacterium OPB45 uid68283	NC_015682	-
1366	Thermodesulfobium narugense DSM 14796 uid66601	NC_015499	-
1367	Thermodesulfovibrio yellowstonii DSM 11347 uid59257	NC_011296	-
1368	Thermofilum pendens Hrk 5 uid58563	NC_008698	-
1369	Thermomicrobium roseum DSM 5159 uid59341	NC_011959	NC_011961

Continued on next page

A list of chromosomes and plasmids analysed in the main text.

	Genome Name	Chromosome Accession IDs	Plasmid Accession IDs
1370	Thermomonospora curvata DSM 43183 uid41885	NC_013510	-
1371	Thermoplasma acidophilum DSM 1728 uid61573	NC_002578	-
1372	Thermoplasma volcanium GSS1 uid57751	NC_002689	-
1373	Thermoproteus neutrophilus V24Sta uid58421	NC_010525	-
1374	Thermoproteus tenax Kra 1 uid74443	NC_016070	-
1375	Thermoproteus uzoniensis 768 20 uid65089	NC_015315	-
1376	Thermosediminibacter oceani DSM 16646 uid51421	NC_014377	-
1377	Thermosipho africanus TCF52B uid59095	NC_011653	-
1378	Thermosipho melanesiensis BI429 uid58683	NC_009616	-
1379	Thermosphaera aggregans DSM 11486 uid48993	NC_014160	-
1380	Thermosynechococcus elongatus BP 1 uid57907	NC_004113	-
1381	Thermotoga RQ2 uid58935	NC_010483	-
1382	Thermotoga lettingae TMO uid58419	NC_009828	-
1383	Thermotoga maritima MSB8 uid57723	NC_000853	-
1384	Thermotoga naphthophila RKU 10 uid42777	NC_013642	-
1385	Thermotoga neapolitana DSM 4359 uid59065	NC_011978	-
1386	Thermotoga petrophila RKU 1 uid58655	NC_009486	-
1387	Thermotoga thermarum DSM 5069 uid68449	NC_015707	-
1388	Thermovibrio ammonificans HB 1 uid62095	NC_014926	NC_014917
1389	Thermovirga lienii DSM 17291 uid77129	NC_016148	NC_016149
1390	Thermus scotoductus SA 01 uid62273	NC_014974	NC_014975
1391	Thermus thermophilus HB27 uid58033	NC_005835	NC_005838
1392	Thermus thermophilus HB8 uid58223	NC_006461	NC_006462, NC_006463
1393	Thioalkalimicrobium cyclicum ALM1 uid67391	NC_015581	-
1394	Thioalkalivibrio K90mix uid46181	NC_013889	NC_013930
1395	Thioalkalivibrio sulfidophilus HL EbGr7 uid59179	NC_011901	-
1396	Thiobacillus denitrificans ATCC 25259 uid58189	NC_007404	-
1397	Thiomicrospira crunogena XCL 2 uid58183	NC_007520	-
1398	Thiomonas intermedia K12 uid48825	NC_014153	NC_014154, NC_014155
1399	Tolumonas auensis DSM 9187 uid59395	NC_012691	-
1400	Treponema azotonutricium ZAS 9 uid67365	NC_015577	-
1401	Treponema brennaborense DSM 12168 uid66607	NC_015500	-
1402	Treponema denticola ATCC 35405 uid57583	NC_002967	-
1403	Treponema pallidum Nichols uid57585	NC_000919	-
1404	Treponema pallidum SS14 uid58977	NC_010741	-
1405	Treponema paraluiscuniculi Cuniculi A uid68447	NC_015714	-
1406	Treponema primitia ZAS 2 uid67367	NC_015578	-
1407	Treponema succinifaciens DSM 2489 uid65781	NC_015385	NC_015386
1408	Trichodesmium erythraeum IMS101 uid57925	NC_008312	-
1409	Tropheryma whipplei TW08 27 uid57961	NC_004551	-

Continued on next page

A list of chromosomes and plasmids analysed in the main text.

	Genome Name	Chromosome Accession IDs	Plasmid Accession IDs
1410	Tropheryma whipplei Twist uid57705	NC_004572	-
1411	Truepera radiovictrix DSM 17093 uid49533	NC_014221	-
1412	Tsukamurella paurometabola DSM 20162 uid48829	NC_014158	NC_014159
1413	Ureaplasma parvum serovar 3 ATCC 27815 uid58887	NC_010503	-
1414	Ureaplasma parvum serovar 3 ATCC 700970 uid57711	NC_002162	-
1415	Ureaplasma urealyticum serovar 10 ATCC 33699 uid59011	NC_011374	-
1416	Variovorax paradoxus EPS uid62107	NC_014931	-
1417	Variovorax paradoxus S110 uid59437	NC_012791, NC_012792	-
1418	Veillonella parvula DSM 2008 uid41927	NC_013520	-
1419	Verminephrobacter eiseniae EF01 2 uid58675	NC_008786	NC_008771
1420	Verrucosispora maris AB 18 032 uid66297	NC_015434	NC_015409
1421	Vibrio Ex25 uid41601	NC_013456, NC_013457	-
1422	Vibrio anguillarum 775 uid68057	NC_015633, NC_015637	-
1423	Vibrio cholerae M66 2 uid59355	NC_012578, NC_012580	-
1424	Vibrio cholerae MJ 1236 uid59387	NC_012667, NC_012668	-
1425	Vibrio cholerae O1 2010EL 1786 uid78933	NC_016445, NC_016446	-
1426	Vibrio cholerae O1 biovar El Tor N16961 uid57623	NC_002505, NC_002506	-
1427	Vibrio cholerae O395 uid58425	NC_009456, NC_009457	-
1428	Vibrio fischeri ES114 uid58163	NC_006840, NC_006841	NC_006842
1429	Vibrio fischeri MJ11 uid58907	NC_011184, NC_011186	NC_011185
1430	Vibrio harveyi ATCC BAA 1116 uid58957	NC_009783, NC_009784	NC_009777
1431	Vibrio parahaemolyticus RIMD 2210633 uid57969	NC_004603, NC_004605	-
1432	Vibrio splendidus LGP32 uid59353	NC_011744, NC_011753	-
1433	Vibrio vulnificus CMCP6 uid62909	NC_004459, NC_004460	-
1434	Vibrio vulnificus MO6 24 O uid62243	NC_014965, NC_014966	-
1435	Vibrio vulnificus YJ016 uid58007	NC_005139, NC_005140	NC_005128
1436	Vulcanisaeta distributa DSM 14429 uid52827	NC_014537	-
1437	Vulcanisaeta moutnovskia 768 28 uid63631	NC_015151	-
1438	Waddlia chondrophila WSU 86 1044 uid49531	NC_014225	NC_014226
1439	Weeksella virosa DSM 16922 uid63627	NC_015144	-
1440	Weissella koreensis KACC 15510 uid68837	NC_015759	NC_015756
1441	Wigglesworthia glossinidia endosymbiont of Glossina brevipalpis uid57853	NC_004344	NC_003425
1442	Wolbachia endosymbiont TRS of Brugia malayi uid58107	NC_006833	-
1443	Wolbachia endosymbiont of Culex quinquefasciatus Pel uid61645	NC_010981	-
1444	Wolbachia endosymbiont of Drosophila melanogaster uid57851	NC_002978	-
1445	Wolbachia wRi uid59371	NC_012416	-

Continued on next page

A list of chromosomes and plasmids analysed in the main text.

	Genome Name	Chromosome Accession IDs	Plasmid Accession IDs
1446	Wolinella succinogenes DSM 1740 uid61591	NC_005090	-
1447	Xanthobacter autotrophicus Py2 uid58453	NC_009720	NC_009717
1448	Xanthomonas albilineans GPE PC73 uid43163	NC_013722	-
1449	Xanthomonas axonopodis citri 306 uid57889	NC_003919	NC_003921, NC_003922
1450	Xanthomonas axonopodis citrumelo F1 uid73179	NC_016010	-
1451	Xanthomonas campestris 8004 uid57595	NC_007086	-
1452	Xanthomonas campestris ATCC 33913 uid57887	NC_003902	-
1453	Xanthomonas campestris B100 uid61643	NC_010688	-
1454	Xanthomonas campestris vesicatoria 85 10 uid58321	NC_007508	NC_007505, NC_007506, NC_007507
1455	Xanthomonas oryzae KACC 10331 uid58155	NC_006834	-
1456	Xanthomonas oryzae MAFF 311018 uid58547	NC_007705	-
1457	Xanthomonas oryzae PXO99A uid59131	NC_010717	-
1458	Xenorhabdus bovienii SS 2004 uid46345	NC_013892	-
1459	Xenorhabdus nematophila ATCC 19061 uid49133	NC_014228	NC_014170
1460	Xylanimonas cellulosilytica DSM 15894 uid41935	NC_013530	NC_013531
1461	Xylella fastidiosa 9a5c uid57849	NC_002488	NC_002490
1462	Xylella fastidiosa M12 uid58763	NC_010513	-
1463	Xylella fastidiosa M23 uid58809	NC_010577	NC_010579
1464	Xylella fastidiosa Temecula1 uid57869	NC_004556	-
1465	Yersinia enterocolitica 8081 uid57741	NC_008800	NC_008791
1466	Yersinia enterocolitica palearctica 105 5R r uid63663	NC_015224	NC_015475
1467	Yersinia pestis Angola uid58485	NC_010159	NC_010157, NC_010158
1468	Yersinia pestis Antiqua uid58607	NC_008150	NC_008120, NC_008121, NC_008122
1469	Yersinia pestis CO92 uid57621	NC_003143	NC_003131, NC_003132, NC_003134
1470	Yersinia pestis KIM 10 uid57875	NC_004088	NC_004838
1471	Yersinia pestis Nepal516 uid58609	NC_008149	NC_008118, NC_008119
1472	Yersinia pestis Pestoides F uid58619	NC_009381	NC_009377, NC_009378
1473	Yersinia pestis Z176003 uid47317	NC_014029	NC_014017, NC_014022, NC_014027
1474	Yersinia pestis biovar Microtus 91001 uid58037	NC_005810	NC_005813, NC_005814, NC_005815, NC_005816
1475	Yersinia pseudotuberculosis IP 31758 uid58487	NC_009708	NC_009704, NC_009705
1476	Yersinia pseudotuberculosis IP 32953 uid58157	NC_006155	NC_006153, NC_006154
1477	Yersinia pseudotuberculosis PB1 uid59153	NC_010634	NC_010635
1478	Yersinia pseudotuberculosis YPIII uid59151	NC_010465	-
1479	Zobellia galactanivorans uid70621	NC_015844	-
1480	Zunongwangia profunda SM A87 uid48073	NC_014041	-

Continued on next page

A list of chromosomes and plasmids analysed in the main text.

	Genome Name	Chromosome Accession IDs	Plasmid Accession IDs
1481	Zymomonas mobilis NCIMB 11163 uid41019	NC_013355	NC_013356, NC_013357, NC_013358
1482	Zymomonas mobilis ZM4 uid58095	NC_006526	-
1483	Zymomonas mobilis pomaceae ATCC 29192 uid68445	NC_015709	NC_015715, NC_015716
1484	cyanobacterium UCYN A uid43697	NC_013771	-
1485	gamma proteobacterium HdN1 uid51635	NC_014366	-
1486	halophilic archaeon DL31 uid72619	NC_015954	NC_015955, NC_015959
1487	uncultured Termite group 1 bacterium phylotype Rs D17 uid59059	NS_000191	-
