# Supplementary material for: Approximate search for known gene clusters in new genomes using PQ-trees
Source: Algorithms Mol Biol. 2021 Jul 9;16:16. doi: 10.1186/s13015-021-00190-9 (PMC8272295; doi:10.1186/s13015-021-00190-9)
Supplement: Supplementary file 2 — Additional file 2. A list of chromosomes and plasmids analysed in the main text. [file 13015_2021_190_MOESM2_ESM.pdf]

# Approximate Search for Known Gene Clusters in New Genomes Using PQ-Trees

## Supplementary Materials

G. R. Zimerman, D. Svetlitsky, M. Zehavi*, and M. Ziv-Ukelson*

Department of Computer Science, Ben Gurion University of the Negev, Be'er Sheva

## Contents

**1 The Q-Node Mapping Algorithm** **2**
1.1 Q-Node Mapping: The Algorithm . . . . . . . . . . . . . . . . . . . . . . . . . . . . 2
1.2 Correctness of the Q-Node Mapping Algorithm . . . . . . . . . . . . . . . . . . . . . 3
1.3 Time and Space Complexity of the Q-Node Mapping Algorithm . . . . . . . . . . . . 6

**2 Penalizing Deletions** **7**

**3 The Naïve Solution** **8**

**4 Additional Figures and Tables** **9**

## List of Figures

S1 The plasmid instances of the heavy metal efflux pump gene cluster and the functional description of its COGs . . . . . . . . . . . . . . . . . . . . . . . . . . . . . . . . . . . 9

## List of Tables

S1 PQ-trees for which tree-guided rearrangements were found in plasmids. . . . . . . . . . 11

*M.Z. and M.Z.U are corresponding authors

# 1 The Q-Node Mapping Algorithm

In this section the Q-mapping algorithm (called by the main algorithm described in Section "The main algorithm" of the main text) is given along with its proof of correctness and complexity analysis.

## 1.1 Q-Node Mapping: The Algorithm

The input and output of the Q-mapping algorithm is the same as the input and output of the P-mapping algorithm (Section "P-node mapping: the algorithm" of the main text), except for the type of $x$, the node received as input. In the description of the algorithm the terms previously defined in Section "P-node mapping: terminology" of the main text are used as well as the following. Given that the children of $x$ in consecutive order are $x_1, x_2, \ldots, x_\gamma$ and given an index $1 \leq i \leq \gamma$, $x_{[i]}$ denotes the set of the first $i$ children of $x$. Formally, $x_{[i]} \doteq \{x_1, \ldots, x_i\}$. In addition, $x^{(i)}$, denotes the node $x$ as if its only children are the nodes in $x_{[i]}$. Consequentially, the span of $x^{(i)}$ is defined as $\sum_{j=1}^{i} \mathsf{span}(x_j)$ and the set $\mathcal{D}(x^{(i)}, k_T, k_S)$ (in Definition 2 of the main text, where $U = \{x^{(i)}\}$) now refers to a set of *partial* derivations. To use $x^{(i)}$ to describe the base cases of the algorithm, let us define $x^{(0)}$ ($x^{(i)}$ for $i = 0$) as a tree with no labeled leaves to map.

The algorithm (whose pseudocode is given in Algorithm S1) constructs two 3-dimensional DP tables $\mathcal{Q}_\ell$ and $\mathcal{Q}_r$. Both have an entry for every $0 \leq k_T \leq d_T$, $0 \leq k_S \leq d_S$ and index $0 \leq i \leq \gamma$. The purpose of an entry $\mathcal{Q}_\ell[i, k_T, k_S]$ (and $\mathcal{Q}_r[i, k_T, k_S]$) is to hold the best score of a partial derivation in $\mathcal{D}(x^{(i)}, k_T, k_S)$, i.e. a partial derivation rooted in $x^{(i)}$ to a prefix of $S'$ with exactly $k_T$ deletions from the tree and $k_S$ deletions from the string. The children of $x$ that are not in $x_{[i]}$ are ignored under the partial derivation stored by the DP table entry $\mathcal{Q}_\ell[i, k_T, k_S]$ (and $\mathcal{Q}_r[i, k_T, k_S]$). The difference between $\mathcal{Q}_\ell$ and $\mathcal{Q}_r$ is in the order in which the children of $x$ are arranged. In $\mathcal{Q}_\ell$ the children of $x$ are considered in a left-to-right order, namely, $x_1$ is the leftmost child of $x$ and $x_{[i]}$ is the set of the $i$ leftmost children of $x$. In $\mathcal{Q}_r$ the children of $x$ are considered in a right-to-left order, namely, $x_1$ is the rightmost child of $x$ and $x_{[i]}$ is the set of the $i$ rightmost children of $x$. For abbreviation, from now on $\mathcal{Q}$ is used when a notion is true for both $\mathcal{Q}_\ell$ and $\mathcal{Q}_r$. Similarly to the main algorithm and the P-mapping algorithm, some of the entries of the DP tables are invalid, and their value is defined as $-\infty$. Formally, an entry $\mathcal{Q}[i, k_T, k_S]$ is invalid if one of the following is true: $k_T > \sum_{c \in x_{[i]}} \mathsf{span}(c)$, $k_S > L(x^{(i)}, k_T, k_S)$, $L(x^{(i)}, k_T, k_S) > \mathsf{len}(S')$, or $L(x^{(i)}, k_T, k_S) < 0$.

For every $0 \leq k_S \leq \mathsf{len}(S')$, the entry $\mathcal{Q}[0, 0, k_S]$ is initialized with 0. These entries of the DP table capture the cases in which a prefix of $S'$ of length $L(x^{(\emptyset)}, 0, k_S) = k_S$ is derived and there are no leaves to map. Thus, all the characters in $S'[1 : k_S]$ must be deleted under the partial derivation. This is possible because the allowed number of deletions from the string is exactly the number of characters in the derived substring. Note that $k_S \leq \mathsf{len}(S')$ because otherwise $\mathcal{Q}[0, 0, k_S]$ is an invalid entry and its value should remain $-\infty$.

After the initialization, the remaining entries of $\mathcal{Q}$ are calculated using the recursion rule in Eq. (1) ahead. The order of computation is ascending with respect to the child index $i$, and for a given $i$, the order of computation is ascending with respect to the number of deletions from the string.

$$\mathcal{Q}[i, k_T, k_S] = \max \begin{cases} \mathcal{Q}[i, k_T, k_S - 1] \\ \mathcal{Q}[i-1, k_T - \mathsf{span}(x_i), k_S] \\ \max\limits_{\substack{\mu \in \mathcal{D}_{\leq}(x_{[i]}, k_T, k_S) \\ \text{s.t. } \mu.v = x_i}} \mathcal{Q}[i-1, k_T - \mu.del_T, k_S - \mu.del_S] + \mu.score \end{cases} \tag{1}$$

Intuitively, every entry $\mathcal{Q}[i, k_T, k_S]$ defines some index $e' = E_I(x^{(i)}, k_T, k_S)$ of $S'$ that is the end point of every partial derivation $\mu \in \mathcal{D}(x^{(i)}, k_T, k_S)$. Thus, $S'[e']$ is either deleted under $\mu$ (the first case) or it is mapped under $\mu$. If $S'[e']$ is mapped under $\mu$, then due to the hierarchical structure of $T(x)$, it must be mapped under some derivation $\mu'$ of one of the children of $x$ that are in $x_{[i]}$.

**Algorithm S1:** Q-Mapping

**Input:** $x, S', \mathcal{D}, d_T, d_S$

**Output:** A best derivation of $x$ to every prefix of $S'$

$\gamma \leftarrow |\mathsf{children}(x)|$;
Build $\mathcal{Q}_\ell$ and $\mathcal{Q}_r$ each with dimensions $\gamma + 1 \times d_T + 1 \times d_S + 1$;
**for** $k_S = 0$ **to** $\mathsf{len}(S')$ **do**
    //Initialization
    $\mathcal{Q}_\ell[0, 0, k_S] \leftarrow 0$;
    $\mathcal{Q}_r[0, 0, k_S] \leftarrow 0$;
**end**
**for** $i = 1$ **to** $\gamma$ **do**
    **for** $k_S = 0$ **to** $d_S$ **do**
        **for** $k_T = 0$ **to** $d_T$ **do**
            Consider the children of $x$ from left to right and compute $\mathcal{Q}_\ell[i, k_T, k_S]$ according to Eq. (1);
            Consider the children of $x$ from right to left and compute $\mathcal{Q}_r[i, k_T, k_S]$ according to Eq. (1);
        **end**
    **end**
**end**
**return** $\max\{\mathcal{Q}_r[\gamma, k_T, k_S], \mathcal{Q}_\ell[\gamma, k_T, k_S]\}$ *for every* $0 \leq k_T \leq d_T$ *and* $0 \leq k_S \leq d_S$ ;

Additionally, the partial derivation $\mu$ does not ignore $x_i$, so either it is deleted under $\mu$ (the second case), or it is kept under $\mu$. Therefore, if $x_i$ is kept under $\mu$ and $S'[e']$ is mapped under $\mu$, then $\mu'$ must be a derivation of $x_i$ (otherwise, the sequential order of the children of $x$ is defied). Thus, we receive the third case of the recursion rule.

Once both DP tables are filled, the algorithm iterates over them and for every deletion numbers combination $k_T, k_S$, it returns the maximum between $\mathcal{Q}_\ell[\gamma, k_T, k_S]$ and $\mathcal{Q}_r[\gamma, k_T, k_S]$.

## 1.2 Correctness of the Q-Node Mapping Algorithm

In this section the correctness of the Q-mapping algorithm presented in Section 1.1 is asserted by proving Lemma 1. The main lemma of the proof (Lemma 1 below) and its proof refer to the DP table $\mathcal{Q}$. Namely, they are true for $\mathcal{Q}_\ell$ considering the children of $x$ in a left-to-right order and for $\mathcal{Q}_r$ considering the children of $x$ in a right-to-left order. For example, when the first $i$ children of $x$ ($x_{[i]}$) are mentioned, in the context of $\mathcal{Q}_\ell$ we refer to the $i$ leftmost children of $x$ and in the context of $\mathcal{Q}_r$ to the $i$ rightmost children.

**Lemma 1.** *At the end of the algorithm every entry of the DP table $\mathcal{Q}$, $\mathcal{Q}[i, k_T, k_S]$, holds the best score of a derivation of $x^{(i)}$ and a prefix of $S'$ with $k_T$ deletions from the tree and $k_S$ deletions from the string, i.e.* $\mathcal{Q}[i, k_T, k_S] = \max_{\mu \in \mathcal{D}(x^{(i)}, k_T, k_S)} \mu.score$.

For the purpose of the proof of the lemma, the following definitions are needed (along with the operations defined in Section "Correctness of our algorithms" of the main text). Given a partial derivation $\mu$, which derives a string $S$, let us define the removal and addition of a deleted node: $\mathsf{removeDel}(\mu, x)$ and $\mathsf{addDel}(\mu, x)$.

**Operation 1.** The operation $\mathsf{removeDel}(\mu, x)$ is defined only if $x$ is deleted under $\mu$. The operation returns a partial derivation $\mu'$ with $\mu.del_T - \mathsf{span}(x)$ deletions from the tree and $\mu.del_S$ deletions from the string. The derivation $\mu'$ ignores $x$ and derives the same substring derived by $\mu$. The one-to-one mapping that yields $\mu'$ is $\mu.o \setminus \{(\ell, \varepsilon) : \ell \in \mathsf{leaves}(x)\}$.

**Operation 2.** The operation $\mathsf{addDel}(\mu, x)$ is defined only if $x$ is ignored under $\mu$. The operation returns a partial derivation $\mu'$ with $\mu.del_T + \mathsf{span}(x)$ deletions from the tree. The substring derived by $\mu'$ is equal to the substring derived by $\mu$. The one-to-one mapping that yields $\mu'$ is $\mu.o \cup \{(\ell, \varepsilon) : \ell \in \mathsf{leaves}(x)\}$.

*Proof.* We prove Lemma 1 by induction on the entries of $\mathcal{Q}$ in the order described in the algorithm. Namely, for two entries $\mathcal{Q}[i_1, k_{T_1}, k_{S_1}]$ and $\mathcal{Q}[i_2, k_{T_2}, k_{S_2}]$, $\mathcal{Q}[i_1, k_{T_1}, k_{S_1}] < \mathcal{Q}[i_2, k_{T_2}, k_{S_2}]$ if and only if

- $i_1 < i_2$, or
- $i_1 = i_2$ and $k_{S_1} < k_{S_2}$, or
- $i_1 = i_2$ and $k_{S_1} = k_{S_2}$ and $k_{T_1} < k_{T_2}$.

**Base Case.** The base case is the initialization of the DP table entries $\mathcal{Q}[0, 0, k_S]$ for $0 \le k_S \le \mathsf{len}(S')$, with a value of 0. Each of these entries holds the score of some derivation $\mu$ of $x^{(0)}$, i.e. $\mu$ is a partial derivation that ignores all nodes in $T(x)$. In addition $\mu$ derives the substring $S'[1 : L(x^{(0)}, 0, k_S)] = S'[1 : k_S]$. Hence, all the characters in $S'[1 : k_S]$ must be deleted under $\mu$. Each deletion does not add to the score of the derivation and there are no mappings under $\mu$ either, so the score of such a derivation is 0.

**Induction Assumption.** Assume that every table entry $\mathcal{Q}[i', k'_T, k'_S]$ such that $\mathcal{Q}[i', k'_T, k'_S] < \mathcal{Q}[i, k_T, k_S]$ holds the best score of a derivation from the set $\mathcal{D}(x^{(i')}, k'_T, k'_S)$. Namely, $\mathcal{Q}[i', k'_T, k'_S] = \max_{\mu \in \mathcal{D}(x^{(i')}, k'_T, k'_S)} \mu.score = OPT(i', k'_T, k'_S)$.

**Induction Step.** Towards the proof of the step, we prove the following Eq. (2):

$$\begin{aligned} OPT(i, k_T, k_S) = \max(&OPT(i, k_T, k_S - 1), \\ &OPT(i-1, k_T - \mathsf{span}(x_i), k_S), \\ &\max_{\substack{\mu \in \mathcal{D}_{\le}(x_{[i]}, k_T, k_S) \\ \text{s.t. } \mu.v = x_i}} OPT(i-1, k_T - \mu.del_T, k_S - \mu.del_S) + \mu.score) \end{aligned} \tag{2}$$

$\le$: Let $\mu^* \in \mathcal{D}(x^{(i)}, k_T, k_S)$ be a derivation such that $\mu^*.score = OPT(i, k_T, k_S)$, and let $e_i = E_I(x^{(i)}, k_T, k_S)$. By definition, $\mu^*$ is a derivation of $x^{(i)}$ to the string $S'[1 : e_i]$. Every child of the root of the derivation is either deleted or kept and every character of the derived string is either deleted or mapped. Thus, $x_i \in \mathsf{children}(x^{(i)})$ is either deleted or kept under $\mu^*$, and the character $S'[e_i]$ is either deleted or mapped under $\mu^*$. Now, let us consider every case.

First, consider a case in which $x_i$ is deleted under $\mu^*$. By removing the deletion of $x_i$ from $\mu^*$ ($\mathsf{removeDel}(\mu^*, x_i)$) a partial derivation $\mu'$ that ignores $x_i$ is obtained, therefore the root of $\mu'$ is $x^{(i-1)}$. By Operation 1, $\mu'$ has $\mu^*.del_T - \mathsf{span}(x_i) = k_T - \mathsf{span}(x_i)$ deletions from the tree and $\mu^*.del_S = k_S$ deletions from the string. Hence, $\mu' \in \mathcal{D}(x^{(i-1)}, k_T - \mathsf{span}(x_i), k_S)$ and Eq. (3) below is true (remember that a deletion of a node does not change the score of a derivation).

$$\begin{aligned} \mu^*.score = \mu'.score &\le OPT(i-1, k_T - \mathsf{span}(x_i), k_S) \\ &\le \max(OPT(i, k_T, k_S - 1), OPT(i-1, k_T - \mathsf{span}(x_i), k_S), \\ &\quad \max_{\substack{\mu \in \mathcal{D}_{\le}(x_{[i]}, k_T, k_S) \\ \text{s.t. } \mu.v = x_i}} OPT(i-1, k_T - \mu.del_T, k_S - \mu.del_S) + \mu.score) \end{aligned} \tag{3}$$

Second, consider a case in which $S'[e_i]$ is deleted under $\mu^*$. By removing the deletion of $S'[e_i]$ from $\mu^*$, $\mathsf{removeDel}(\mu^*, e_i)$ (see Operation 3 in the main text), the partial derivation obtained, $\mu'$, has $k_T$ and $k_S - 1$ deletions from the tree and string, respectively, and its root is $x^{(i)}$. Hence,

$\mu' \in \mathcal{D}(x^{(i)}, k_T, k_S - 1)$ and Eq. (4) below is true (remember that a deletion of a character does not change the score of a derivation).

$$\begin{aligned} \mu^*.score = \mu'.score &\leq OPT(i, k_T, k_S - 1) \\ &\leq \max(OPT(i, k_T, k_S - 1), OPT(i-1, k_T - \mathsf{span}(x_i), k_S), \\ &\max_{\substack{\mu \in \mathcal{D}_{\leq}(x_{[i]}, k_T, k_S) \\ \text{s.t. } \mu.v = x_i}} OPT(i-1, k_T - \mu.del_T, k_S - \mu.del_S) + \mu.score) \end{aligned} \tag{4}$$

Lastly, if neither is true, then $S'[e_i]$ is mapped under $\mu^*$ and $x_i$ is kept under $\mu^*$. Let $\mu_i$ be the derivation of $x_i$ under $\mu^*$ (there is one because $x_i$ is kept under $\mu^*$). Because $S'[e_i]$ is mapped, then it is a part of a substring of $S'[1 : e_i]$ that is derived by some derivation, $\mu_j$, such that $\mu_j$ is the derivation of the child node $\mu_j.v \in \mathsf{children}(x^{(i)})$ under $\mu^*$. Since $x_i$ is the last child of $x^{(i)}$ and the children of the Q-node $x$ can only be arranged sequentially, $\mu_j.v$ must be $x_i$ (otherwise, the sequential order is defied). Every child of $x$ can only have one derivation under $\mu^*$ (otherwise, it is not a one-to-one mapping), so $\mu_i = \mu_j$. Note that $\mu_i$ must have up to $k_T$ and $k_S$ deletions from the tree and string, respectively, else $\mu^*$ is not a legal derivation. In addition, the end point of $\mu_i$ is $e_i$. Let $\mu_i^*$ be the highest scoring derivation of $x_i$ with up to $k_T$ and $k_S$ deletions which has the end point $e_i$, i.e $\mu_i.score \leq \mu_i^*.score$. By definition, $\mu_i^* \in \mathcal{D}_{\leq}(x^{(i)}, k_T, k_S)$, hence $\mu_i.score \leq \max_{\mu \in \mathcal{D}_{\leq}(x^{(i)}, k_T, k_S)} \mu.score$. Now, removing $\mu_i$ from $\mu^*$, $\mathsf{remove}(\mu^*, \mu_i)$ (see Operation 1 in the main text), results in a derivation, $\mu'$, with $\mu^*.del_T - \mu_i.del_T = k_T - \mu_i.del_T$ deletions from the tree and $\mu^*.del_S - \mu_i.del_S = k_S - \mu_i.del_S$ deletions from the string. In addition $\mu'$ ignores $x_i$, and so its root is $x^{(i-1)}$. Hence, similarly to $\mu_i$, $\mu'.score \leq \max_{\mu \in \mathcal{D}(x^{(i-1)}, k_T - \mu_i.del_T, k_S - \mu_i.del_S)} \mu.score = OPT(i-1, k_T - \mu_i.del_T, k_S - \mu_i.del_S)$. Putting the conclusions on $\mu_i$ and $\mu'$ together we obtain Eq. (5) below.

$$\begin{aligned} \mu^*.score &= \mu_i.score + \mu'.score \\ &\leq \max_{\substack{\mu \in \mathcal{D}_{\leq}(x_{[i]}, k_T, k_S) \\ \text{s.t. } \mu.v = x_i}} \mu.score + OPT(i-1, k_T - \mu_i.del_T, k_S - \mu_i.del_S) \\ &\leq \max_{\substack{\mu \in \mathcal{D}_{\leq}(x_{[i]}, k_T, k_S) \\ \text{s.t. } \mu.v = x_i}} OPT(i-1, k_T - \mu.del_T, k_S - \mu.del_S) + \mu.score \\ &\leq \max(OPT(i, k_T, k_S - 1), OPT(i-1, k_T - \mathsf{span}(x_i), k_S), \\ &\max_{\substack{\mu \in \mathcal{D}_{\leq}(x_{[i]}, k_T, k_S) \\ \text{s.t. } \mu.v = x_i}} OPT(i-1, k_T - \mu.del_T, k_S - \mu.del_S) + \mu.score) \end{aligned} \tag{5}$$

In any case Eq. (6) below is true.

$$\begin{aligned} OPT(i, k_T, k_S) = \mu^*.score \\ &\leq \max(OPT(i, k_T, k_S - 1), OPT(i-1, k_T - \mathsf{span}(x_i), k_S), \\ &\max_{\substack{\mu \in \mathcal{D}_{\leq}(x_{[i]}, k_T, k_S) \\ \text{s.t. } \mu.v = x_i}} OPT(i-1, k_T - \mu.del_T, k_S - \mu.del_S) + \mu.score) \end{aligned} \tag{6}$$

$\geq$: Let $\mu^*$ be a derivation such that $\mu^*.score = \max(OPT(i, k_T, k_S - 1), OPT(i-1, k_T - \mathsf{span}(x_i), k_S), \max_{\substack{\mu \in \mathcal{D}_{\leq}(x_{[i]}, k_T, k_S) \\ \text{s.t. } \mu.v = x_i}} OPT(i-1, k_T - \mu.del_T, k_S - \mu.del_S) + \mu.score)$. Hence, $\mu^*.score = OPT(i, k_T, k_S - 1)$ or $\mu^*.score = OPT(i-1, k_T - \mathsf{span}(x_i), k_S)$ or $\mu^*.score = \max_{\substack{\mu \in \mathcal{D}_{\leq}(x_{[i]}, k_T, k_S) \\ \text{s.t. } \mu.v = x_i}} OPT(i-1, k_T - \mu.del_T, k_S - \mu.del_S) + \mu.score$, and let $e_i = E_I(x^{(i)}, k_T, k_S)$.

First, assume $\mu^*.score = OPT(i, k_T, k_S - 1)$. Let $\eta \in \mathcal{D}(x^{(i)}, k_T, k_S - 1)$ be a derivation with $\eta.score = OPT(i, k_T, k_S - 1)$. By definition, $\eta$ derives the substring $S'[1 : E_I(x^{(i)}, k_T, k_S - 1)]$. From Operation 4 in the main text, Adding to $\eta$ the deletion of $S'[e_i]$ ($\mathsf{addDel}(\eta, e_i)$) results in a derivation, $\eta'$ that derives $x^{(i)}$ to the string $S'[1 : e_i]$ with $k_T$ deletions from the tree and $k_S$ deletions from the string. The string $S'[1 : e_i]$ is equal to the concatenation of $S'[1 : E_I(x^{(i)}, k_T, k_S - 1)]$ and $S'[e_i]$. So, $\eta' \in \mathcal{D}(x^{(i)}, k_T, k_S)$, and thus $\eta'.score \le OPT(i, k_T, k_S)$. We have thus built $\eta'$ such that $\mu^*.score = \eta'.score$, so $\mu^*.score \le OPT(i, k_T, k_S)$.

Second, assume $\mu^*.score = OPT(i-1, k_T - \mathsf{span}(x_i), k_S)$. Let $\eta \in \mathcal{D}(x^{(i-1)}, k_T - \mathsf{span}(x_i), k_S)$ be a derivation with $\eta.score = OPT(i-1, k_T - \mathsf{span}(x_i), k_S)$. By definition, $\eta$ derives the substring $S'[1 : E_I(x^{(i-1)}, k_T - \mathsf{span}(x_i), k_S)]$. From Operation 2, adding the deletion of the node $x_i$ to $\eta$ ($\mathsf{addDel}(\eta, x_i)$) results in a derivation $\eta'$ that derives $x^{(i)}$ to the string $S'[1 : e_i]$ with $k_T$ deletions from the tree and $k_S$ deletions from the string. So, $\eta' \in \mathcal{D}(x^{(i)}, k_T, k_S)$, and thus $\eta'.score \le OPT(i, k_T, k_S)$. We built $\eta'$ such that $\mu^*.score = \eta'.score$, so $\mu^*.score \le OPT(i, k_T, k_S)$.

Lastly, assume $\mu^*.score = \max\limits_{\substack{\mu \in \mathcal{D}_{\le}(x_{[i]}, k_T, k_S) \\ \text{s.t. } \mu.v = x_i}} OPT(i-1, k_T - \mu.del_T, k_S - \mu.del_S) + \mu.score$. Let $\eta^*$ be a derivation of $x_i$ that is in $\mathcal{D}_{\le}(x_{[i]}, k_T, k_S)$ that by setting $\eta^*$ to $\mu$ yields the highest value for $OPT(i-1, k_T - \mu.del_T, k_S - \mu.del_S) + \mu.score$ of all the derivations of $x_i$ in $\mathcal{D}_{\le}(x_{[i]}, k_T, k_S)$. Formally, $\eta^* = \arg\max_{\substack{\mu \in \mathcal{D}_{\le}(x_{[i]}, k_T, k_S) \\ \text{s.t. } \mu.v = x_i}} OPT(i-1, k_T - \mu.del_T, k_S - \mu.del_S) + \mu.score$. From Operation 2 in the main text, adding $\eta^*$ to a partial derivation $\eta \in \mathcal{D}(x^{(i-1)}, k_T - \eta^*.del_T, k_S - \eta^*.del_S)$ ($\mathsf{add}(\eta, \eta^*)$) results in a partial derivation $\eta'$ with $k_T - \eta^*.del_T + \eta^*.del_T = k_T$ deletions from the tree and $k_S - \eta^*.del_S + \eta^*.del_S = k_S$ deletions from the string, that takes into account the children of $x$ that are in $x_{[i-1]} \cup \{x_i\} = x_{[i]}$. It is a legal partial derivation since $\eta^*$ derives the node $\eta^*.v$ that is not in $x_{[i-1]}$ to a string that does not intersect with the string derived by $\eta$. The string that is derived by $\eta$ is $S'[\eta.s : \eta.e]$ and it does not intersect with the string derived by $\eta^*$ ($S'[\eta^*.s : \eta^*.e]$). That is because $\eta.e + 1 = \eta^*.s$, as can be seen in Eq. (7) below. So, $\eta' \in \mathcal{D}(x^{(i)}, k_T, k_S)$, and thus $\eta'.score \le OPT(i, k_T, k_S)$. We built $\eta'$ such that $\mu^*.score = \eta'.score$, so $\mu^*.score \le OPT(i, k_T, k_S)$.

$$
\begin{aligned}
\eta^*.s &= e_i - L(x_i, \eta^*.del_T, \eta^*.del_S) + 1 \\
&= \sum_{x_j \in x_{[i]}} \mathsf{span}(x_j) + k_S - k_T - (\mathsf{span}(x_i) + \eta^*.del_S - \eta^*.del_T) + 1 \\
&= \sum_{x_j \in x_{[i-1]}} \mathsf{span}(x_j) + k_S - \eta^*.del_S - (k_T - \eta^*.del_T) + 1 \\
&= E_I(x^{(i-1)}, k_T - \eta^*.del_T, k_S - \eta^*.del_S) + 1 = \eta.e + 1
\end{aligned} \tag{7}
$$

From the induction assumption, $\mathcal{Q}[i, k_T, k_S - 1] = OPT(i, k_T, k_S - 1)$, $\mathcal{Q}[i-1, k_T - \mathsf{span}(x_i), k_S] = OPT(i-1, k_T - \mathsf{span}(x_i), k_S)$ and for every $\mu \in \mathcal{D}(x^{(i)}, k_T, k_S)$ such that $\mu.v = x_i$, $\mathcal{Q}[i-1, k_T - \mu.del_T, k_S - \mu.del_S] = OPT(i-1, k_T - \mu.del_T, k_S - \mu.del_S)$. Thus from Eq. (2), it follows that $\mathcal{Q}[i, k_T, k_S] = OPT(i, k_T, k_S)$. This completes the proof. □

### 1.3 Time and Space Complexity of the Q-Node Mapping Algorithm

Here Lemma 3 of the main text is proven, we repeat it here for convenience.

**Lemma 3.** *The Q-mapping algorithm takes $O({d_T}^2 {d_S}^2 \gamma)$ time and $O(d_T d_S \gamma)$ space.*

*Proof.* The DP table is the most space consuming data structure in the described algorithm. Its dimensions are $\gamma + 1 \times d_T + 1 \times d_S + 1$, and the algorithm uses two DP tables. The computation of an entry of the DP table, $\mathcal{Q}[i, k_T, k_S]$, includes two $O(1)$ calculations (the first and second cases of the

recursion rule) and a longer computation going over every derivation of $x_i$ in $\mathcal{D}_{\leq}(x_{[i]}, k_T, k_S)$. All those derivations have the same root and the same end point, but a different number of deletions. In fact, there are no two derivations of $x_i$ in $\mathcal{D}_{\leq}(x_{[i]}, k_T, k_S)$ that have the same deletion combination $(k'_T, k'_S)$. Hence, the number of such derivations is equal to the number of deletion combinations, $k_T \cdot k_S$, and so the calculation of an entry of the DP table takes $O(k_T k_S) = O(d_T d_S)$ time. Thus, the time complexity of the algorithm is $O(\gamma {d_T}^2 {d_S}^2)$.

In the previous paragraph the calculation of $E_I$ for every entry, which yields the relevant end point for the entry, was ignored. The most time consuming part of that calculation is the summation of spans $(\sum_{j=1}^{i} \mathsf{span}(x_j))$ which takes $O(\gamma)$ time. To prevent the wasteful repetition, these summations are calculated once and then saved in a table of size $\gamma$. These summations are calculated twice - once for each possible children ordering (left-to-right and right-to-left). This is negligible with respect to the time it takes to fill the DP table. □

## 2 Penalizing Deletions

To assign deletions a penalty cost (and not only bound them), the algorithm should receive as input a deletion penalty function, $\delta : \Sigma_T \cup \Sigma_S \to \mathbb{R}$. The function defines the penalty of deleting a character from $S$ or a leaf from $T$ according to its label. Let us expand $\delta$ and define the deletion penalty of a node $x$ in $T$ as the summation of the deletion penalty of all the leaves in the subtree rooted in $x$. Thus, the set of nodes in $T$ is denoted by $T.nodes$, and a new function $\Delta : T.nodes \cup \Sigma_S \to \mathbb{R}$ is defined in Eq. (8) below. Note that the $\Delta$ function can be calculated in advance, by going over $T$ in postorder. This calculation takes $O(m') = O(m)$ time.

$$\Delta(x) = \begin{cases} \delta(x), & if\ x \in \Sigma_S \\ \displaystyle\sum_{\ell \in \mathsf{leaves}(x)} \delta(\mathsf{label}(\ell)), & if\ x \in T.nodes \end{cases} \tag{8}$$

In addition, the following changes to the main algorithm and to the P-mapping and Q-mapping algorithms are needed. First, the initialization of the main DP table $\mathcal{A}$ should change and add to the score of every leaf entry (i.e. $\mathcal{A}[x, i, k_T, k_S]$ such that $x$ is a leaf) the cost of the deleted nodes and characters. Namely, in Algorithm 1 (given in Section "The main algorithm" of the main text) lines 6-7 should be replaced with Eq. (9) below. Second, the $\Delta$ function in Eq. (8) should be sent from the main algorithm to the Q-mapping and P-mapping algorithms.

$$\begin{aligned} \mathcal{A}[x, i, 1, k_S] &\leftarrow -\Delta(x) - \sum_{\ell=i}^{i+k_S-1} \Delta(S[\ell]) \\ \mathcal{A}[x, i, 0, k_S] &\leftarrow \max_{i'=i,\ldots,i+d_S} h(x, i') - \sum_{\ell=i}^{i+k_S} \Delta(S[\ell]) + \Delta(S[i']) \end{aligned} \tag{9}$$

Third, the initialization of the DP table, $\mathcal{P}$, and the recursion rule of the P-mapping algorithm need to change. When $L(x^{(C)}, k_T, k_S) = 0$ and $k_S = 0$, then $\mathcal{P}[C, k_T, k_S] = -\sum_{c \in C} \Delta(c)$ because these entries depict cases in which every node in $C$ is deleted. When $C = \emptyset$ and $k_T = 0$, then $\mathcal{P}[C, k_T, k_S] = -\sum_{i=1}^{k_S} \Delta(S'[i])$ because these entries portray the cases in which every character in $S'[1 : k_S]$ is deleted. The recursion rule of the P-mapping algorithm should be changed to the one in Eq. (10). Note that the change is only in the first case where the cost of deleting the $i^{\text{th}}$ character of $S'$ is subtracted from the score.

$$\mathcal{P}[C, k_T, k_S] = \max \begin{cases} \mathcal{P}[C, k_T, k_S - 1] - \Delta(S[E_I(x^{(C)}, k_T, k_S)]) \\ \displaystyle\max_{\mu \in \mathcal{D}_{\leq}(C, k_T, k_S)} \mathcal{P}[C \setminus \{\mu.v\}, k_T - \mu.del_T, k_S - \mu.del_S] + \mu.score \end{cases} \tag{10}$$

Lastly, in the Q-mapping algorithm the initialization of the DP table, $\mathcal{Q}$, and the recursion rule also need to change. In the initialization, for every $0 \leq k_S \leq d_S$, $\mathcal{Q}[0,0,k_S] = -\sum_{i=0}^{k_S} \Delta(S'[i])$. That is because this is the case in which every character in $S'[1:k_S]$ is deleted. When filling the DP table, the recursion rule in Eq. (11) should be used. Note, the change is in the first and second cases. In the first the cost of deleting the $i^{\text{th}}$ character of $S'$ is subtracted from the score, and in the second the score for deleting $x_i$ is subtracted.

$$\mathcal{Q}[i,k_T,k_S] = \max \begin{cases} \mathcal{Q}[i,k_T,k_S-1] - \Delta(S[E_I(x^{(i)},k_T,k_S)]) \\ \mathcal{Q}[i-1,k_T-\mathsf{span}(x_i),k_S] - \Delta(x_i) \\ \max\limits_{\substack{\mu \in \mathcal{D}_{\leq}(x_{[i]},k_T,k_S) \\ \text{s.t. } \mu.v = x_i}} \mathcal{Q}[i-1,k_T-\mu.del_T,k_S-\mu.del_S] + \mu.score \end{cases} \tag{11}$$

## 3 The Naïve Solution

In this section a naïve, alternative, algorithm for the PQ-Tree Search problem is described and its time complexity is analyzed. Thus, it is shown that the time complexity of our algorithm is substantially smaller than that of the naïve algorithm.

Solving the PQ-Tree Search problem requires a search for a one-to-one mapping that yields a derivation of a PQ-tree $T$ to a substring of the input string $S$. That is, a substring $S'$ of $S$, such that the deletion of up to $d_S$ characters from $S'$ and the substitution of some of its characters yields a new string $S'' \in C_{d_T}(T)$ (see the definition in Section "PQ-tree: representing the pattern" of the main text). Hence, a naïve way to solve the problem is to go over every string in $C_{d_T}(T)$ and try to find an alignment between it and every substring of $S$, when only $d_S$ deletions are allowed from $S$. Equivalently, it is possible to search for an alignment between every substring of $S$ and every string $S_T \in C_0(T)$ with up to $d_S$ deletions from $S$ and up to $d_T$ deletions from $S_T$.

Naturally, sequence alignment can be used, but in order to bound the number of deletions, the basic algorithm needs to be modified. The usual 2-dimensional DP table needs to be extended with two additional dimensions that correspond to the numbers of deletions from $S$ and $S_T$. This way, when filling the table, the best scoring alignment considered so far for every deletion numbers combination can be stored. At the end of the algorithm, the score of the best alignment is the maximum between the entries of the DP table corresponding to an alignment between $S_T$ and a prefix of $S$ that has a length between $m - d_T$ and $m + d_S$. Thus, the outline of the naïve algorithm is as follows. For every string $S_T \in C(T)$ and every possible start index $i$, perform sequence alignment with a bounded number of deletions. Then, find the start index $i$ that resulted in the highest scoring alignment.

The size of the DP table is $O(m(m+d_S)d_Td_S)$, but in the first two dimensions only a diagonal with a width of $O(d_T + d_S)$ entries needs to be computed. The computation of each entry takes $O(1)$ time and finding the best alignment takes $O(d_Td_S)$. Thus, every run of the sequence alignment with a bounded number of deletions and a specific start index $i$ takes $O(m(d_T+d_S)d_Td_S)$ time. As seen in Section "The main algorithm" of the main text, there are $O(n)$ possible values for $i$.

Finally, let us bound the number of strings in $C(T)$ which is equal to the number of PQ-trees that are equivalent to $T$. By definition, every legal permutation of the children of an internal node of $T$ results in a new PQ-tree $T'$ that is equivalent to $T$, i.e. $T \equiv T'$ (equivalence and not quasi-equivalence is used here because $C_0(T)$ is considered, i.e. there are no deletions from the tree). The children of a Q-node can be permuted only in one of two ways (left-to-right or right-to-left) and the children of a P-node can be arranged in any order. So, for an internal node $x$ of $T$, for every string resulting from the rearrangement of the children of all the other nodes in $T$, $x$ contributes 2 strings to $C(T)$ if it is a Q-node, and $\gamma!$ strings if it is a P-node. Thus, $|C(T)| = O(2^{m_q}(\gamma!)^{m_p})$. In total, the naïve solution for PQ-Tree Search takes $O(2^{m_q}(\gamma!)^{m_p}nm(d_T+d_S)d_Td_S)$ time.

# 4 Additional Figures and Tables

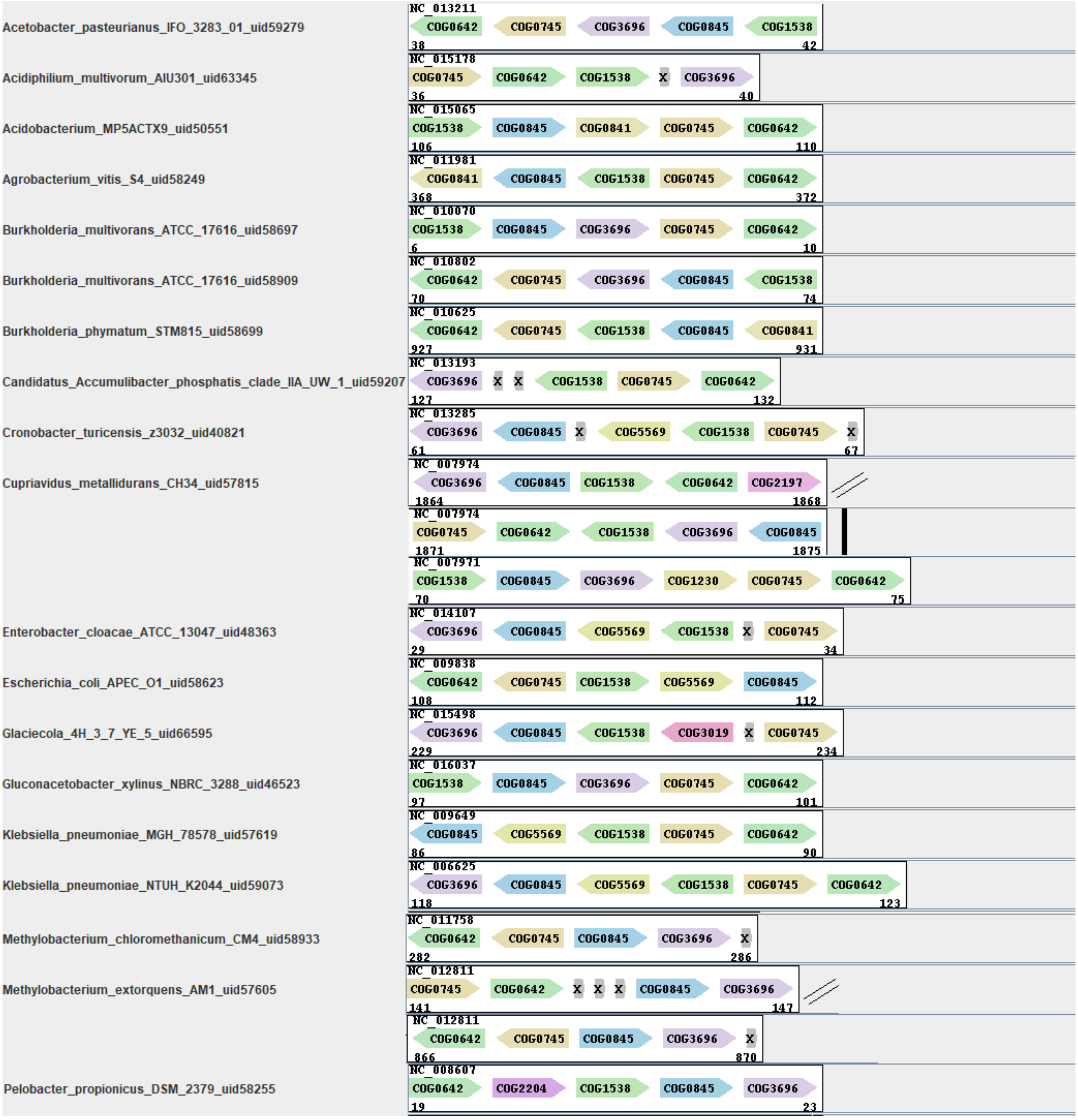


Figure S1: This figure is continued in the next page.

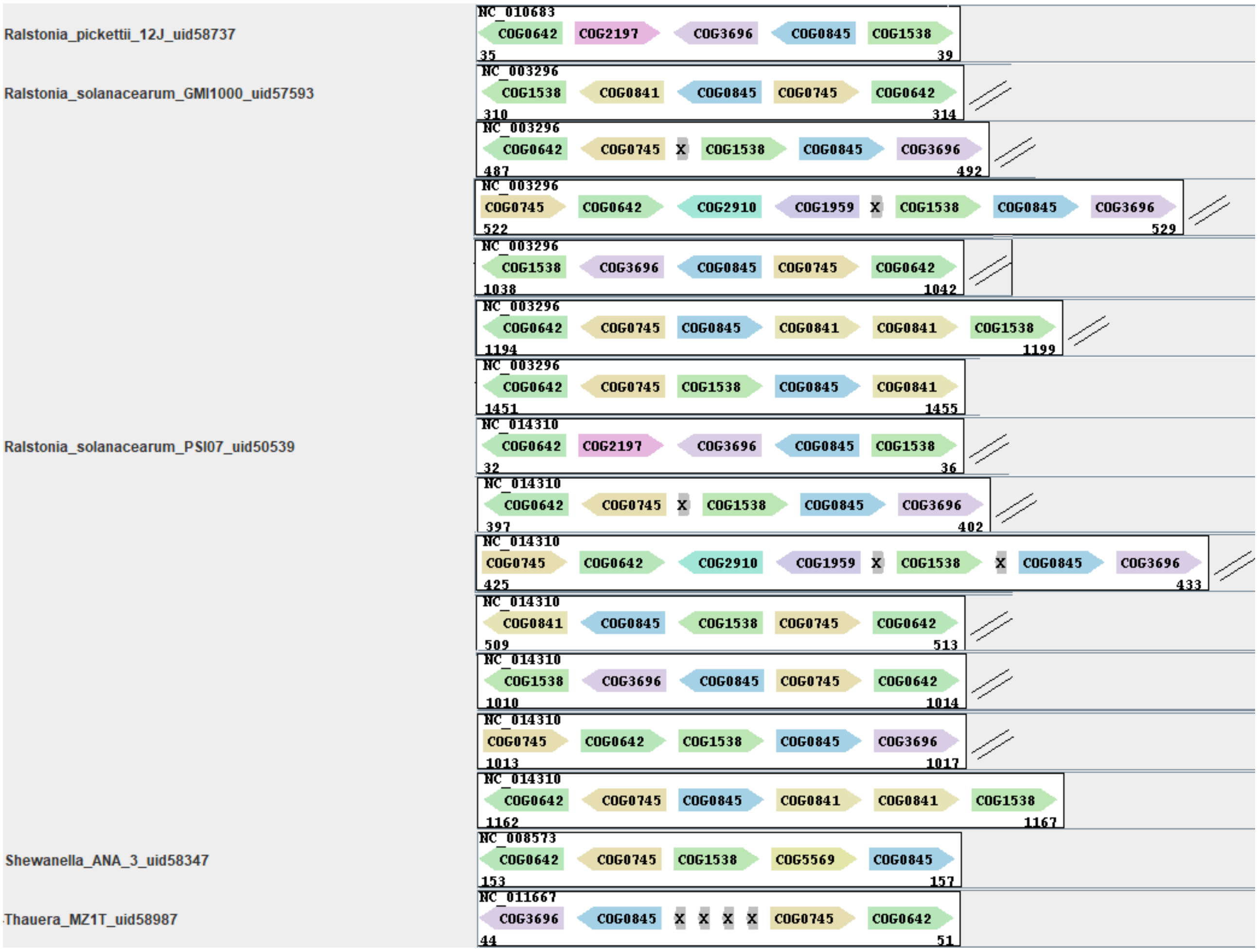


a

COG0642 Signal transduction mechanisms | Signal transduction histidine kinase | BaeS
COG0745 Signal transduction mechanisms/Transcription | DNA-binding response regulator, OmpR family, contains REC and winged-helix (wHTH) domain | OmpR
COG3696 Inorganic ion transport and metabolism | Cu/Ag efflux pump CusA | CusA
COG0845 Cell wall/membrane/envelope biogenesis/Defense mechanisms | Multidrug efflux pump subunit AcrA (membrane-fusion protein) | AcrA
COG1538 Cell wall/membrane/envelope biogenesis | Outer membrane protein TolC | TolC
COG2204 Signal transduction mechanisms | DNA-binding transcriptional response regulator, NtrC family, contains REC, AAA-type ATPase, and a Fis-type DNA-binding domains | AtoC
COG1959 Transcription | DNA-binding transcriptional regulator, IscR family | IscR
COG2910 General function prediction only | Putative NADH-flavin reductase | YwnB
COG5569 Inorganic ion transport and metabolism | Periplasmic Cu and Ag efflux protein CusF | CusF
COG2197 Signal transduction mechanisms/Transcription | DNA-binding response regulator, NarL/FixJ family, contains REC and HTH domains | CitB
COG3019 Function unknown | Uncharacterized conserved protein |
COG0841 Defense mechanisms | Multidrug efflux pump subunit AcrB | AcrB
COG1230 Inorganic ion transport and metabolism | Co/Zn/Cd efflux system component | CzcD

b

Figure S1 (Cont.): **(a)** The plasmid instances of the heavy metal efflux pump gene cluster discussed in Section "Finding approximate instances of an RND efflux pump" of the main text. The COGs of the query gene cluster are: COG0642, COG0745, COG3639, COG0845, COG1538. The instances were identified using PQFinder and displayed using the graphical interface of the tool CSBFinder-S [1]. "X" indicates a gene with no COG annotation. The image was edited to display instances of the same genome in separate lines. Note that the DNA sequence of *Klebsiella pneumoniae* MGH 78578 was updated by the NCBI on Oct 9, 2019, and since then the gene CusA (COG3696) is identified in the corresponding plasmid (Accession ID NC_009649). **(b)** The functional description of the COGs shown in (a).

| | PQ-Tree[1] | S-score | # Genomes[2] | Functional Category |
|---|---|---|---|---|
| 1 | [[0683 [[0411 0410] [0559 4177]]] 0583] | 22.5 | 5 (2) | Amino acid transport |
| 2 | (1609 [1653 1175 0395] 3839) | 10.0 | 10 (2) | Carbohydrate transport |
| 3 | [[1538 [3696 0845]] [0642 0745]] | 7.5 | 7 (1) | Heavy metal efflux |
| 4 | [[2115 1070] [4213 [1129 4214]]] | 7.5 | 1 (1) | Carbohydrate transport |
| 5 | [1960 [[2011 1135] [2141 1464]]] | 7.5 | 3 (1) | Amino acid transport |
| 6 | [[0596 0599] [[3485 3485] 0015]] | 7.5 | 9 (1) | Metabolism |
| 7 | [[[1129 1172 1172] 1879] 3254] | 7.5 | 6 (1) | Carbohydrate transport |
| 8 | (1609 1869 [[1129 1172] 1879] 0524) | 7.5 | 1 (1) | Carbohydrate transport |
| 9 | (0683 [0559 4177] [0411 0410] 0318) | 7.5 | 1 (1) | Amino acid transport |
| 10 | (3839 0673 [[0395 1175] 1653]) | 5.0 | 10 (1) | Carbohydrate transport |
| 11 | [0583 (0687 3842 [1176 1177])] | 5.0 | 9 (3) | Amino acid transport |
| 12 | [1012 (0687 3842 [1176 1177])] | 5.0 | 8 (1) | Amino acid transport |
| 13 | (0284 0461 [0540 1781] 0543 0044 0167) | 3.5 | 1 (1) | Metabolism |
| 14 | ((2080 1319 1529) 1975 2068) | 3.3 | 6 (1) | Energy production and conversion |
| 15 | [0044 [[0543 0167] 0284]] | 3.0 | 1 (1) | Metabolism |
| 16 | [1802 [1638 [3090 1593]]] | 3.0 | 7 (1) | Carbohydrate transport |
| 17 | [0410 [[4177 0559] 0683]] | 3.0 | 7 (3) | Amino acid transport |
| 18 | [[4770 0511] [1984 2049]] | 3.0 | 4 (2) | Metabolism |
| 19 | [[2875 [1010 2073]] 2243] | 3.0 | 9 (2) | Metabolism |
| 20 | ([1175 0395] 1409 3839 1653) | 2.5 | 5 (2) | Carbohydrate transport |
| 21 | [(2141 0431 0600 0715) 1116] | 2.5 | 2 (2) | Inorganic ion transport |
| 22 | ([0601 1173] 0444 0444 0747) | 2.5 | 10 (1) | Amino acid transport |
| 23 | [0583 (3842 1840 1178)] | 2.0 | 1 (1) | Inorganic ion transport |
| 24 | (1464 2141 [1135 2011]) | 2.0 | 7 (3) | Amino acid transport |
| 25 | ([2009 2142] 0479 1053) | 2.0 | 2 (1) | Energy production and conversion |
| 26 | ([1622 0843] 0109 1845) | 2.0 | 1 (1) | Energy production and conversion |
| 27 | (1024 1960 4770 4799) | 1.0 | 4 (1) | Lipid transport |
| 28 | (1120 0609 0614 1629) | 1.0 | 4 (1) | Inorganic ion transport |
| 29 | (0411 0559 4177 0683 0410 1022) | 1.0 | 3 (1) | Amino acid transport |

Table S1: PQ-trees for which tree-guided rearrangements were found in plasmids. [1]Square brackets represent a Q-node; round brackets represent a P-node. Numbers indicate the respective COG IDs. [2]This column indicates the number of genomes harboring plasmid instances of the respective PQ-tree. The number in brackets indicates the number of genomes harboring a tree-guided gene rearrangement of the corresponding gene cluster.

# References

[1] Dina Svetlitsky, Tal Dagan, and Michal Ziv-Ukelson. Discovery of multi-operon colinear syntenic blocks in microbial genomes. *Bioinformatics*, 2020.
